# Using Health Extension Workers for Monitoring Child Mortality in Real-Time: Validation against Household Survey Data in Rural Ethiopia

**DOI:** 10.1371/journal.pone.0126909

**Published:** 2015-11-25

**Authors:** Agbessi Amouzou, Aklilu Kidanu, Nolawi Taddesse, Romesh Silva, Elizabeth Hazel, Jennifer Bryce, Robert E. Black

**Affiliations:** 1 UNICEF, New York, New York, United States of America; 2 Institute for International Programs, Department of International Health, Johns Hopkins Bloomberg School of Public Health, Baltimore, Maryland, United States of America; 3 Miz Hasab Research Center, Addis Ababa, Ethiopia; Tulane University School of Public Health and Tropical Medicine, UNITED STATES

## Abstract

**Background:**

Ethiopia has scaled up its community-based programs over the past decade by training and deploying health extension workers (HEWs) in rural communities throughout the country. Consequently, child mortality has declined substantially, placing Ethiopia among the few countries that have achieved the United Nations’ fourth Millennium Development Goal. As Ethiopia continues its efforts, results must be assessed regularly to provide timely feedback for improvement and to generate further support for programs. More specifically the expansion of HEWs at the community level provides a unique opportunity to build a system for real-time monitoring of births and deaths, linked to a civil registration and vital statistics system that Ethiopia is also developing. We tested the accuracy and completeness of births and deaths reported by trained HEWs for monitoring child mortality over 15 -month periods.

**Methods and Findings:**

HEWs were trained in 93 randomly selected rural kebeles in Jimma and West Hararghe zones of the Oromia region to report births and deaths over a 15-month period from January, 2012 to March, 2013. Completeness of number of births and deaths, age distribution of deaths, and accuracy of resulting under-five, infant, and neonatal mortality rates were assessed against data from a large household survey with full birth history from women aged 15–49. Although, in general HEWs, were able to accurately report events that they identified, the completeness of number of births and deaths reported over twelve-month periods was very low and variable across the two zones. Compared to household survey estimates, HEWs reported only about 30% of births and 21% of under-five deaths occurring in their communities over a twelve-month period. The under-five mortality rate was under-estimated by around 30%, infant mortality rate by 23% and neonatal mortality by 17%. HEWs reported disproportionately higher number of deaths among the very young infants than among the older children.

**Conclusion:**

Birth and death data reported by HEWs are not complete enough to support the monitoring of changes in childhood mortality. HEWs can significantly contribute to the success of a CRVS in Ethiopia, but cannot be relied upon as the sole source for identification of vital events. Further studies are needed to understand how to increase the level of completeness.

## Introduction

Ethiopia has made remarkable progress in child survival over the past three decades and is among the few countries in sub-Saharan Africa that have already achieved Millennium Development Goal number 4 for reducing child mortality [1]. However, like many low-and middle-income countries, Ethiopia does not have a strong monitoring system capable of tracking the short-term survival impact of the health services and programs implemented on a large scale in the country. In 2004, the Ethiopian government trained and deployed over 34,000 Health Extension Workers (HEWs) to each sub-district (called kebele). The kebele population is, on average, 5000 people. These paid female government workers initially provided preventive and promotive health services, and now also manage uncomplicated cases of childhood illnesses such as pneumonia, diarrhea and malaria [2].

Most have completed 10^th^ grade and some have completed high school. They recieve a 12 month training on disease prevention and health promotion before being deployed. They are necessarily recruited from the community they are assigned to work in but, once deployed, most live in the kebele assigned to them. Two HEWs are generally assigned to each health post within a kebele and are expected to spend the majority of their time visiting families in their homes and performing outreach programs in the community. They are supervised by the nearest health center. The HEWs work with a group of several volunteers in each kebele to discuss community issues and identify any vital events in the community. The HEWs generally maintain a logbook or family folders at their post to record family information, including births and deaths in each family in their catchment area.

The deployment of HEWs throughout the country provides a unique opportunity to build a solid system for identification and tracking of births and deaths within communities, linked to a functioning health information system and the Civil Registration and Vital Statistics System (CRVS) that has become a priority for the country [3–5]. While there is global growing interest in using community health workers to support the expansion of the CRVS and its linkage to the health system, very few studies have documented the feasibility of such an approach. Moreover, data reported by these community health workers, already overburdened by community health activities, must be assessed for accuracy and completeness in order to appropriately quantify the usefulness and usability of such approach [6–8]. A recent study in Malawi demonstrated the feasibility of using community health workers to report vital events but also showed limitations in the completeness of events recorded for monitoring child mortality [6]. Furthermore, currently approaches for mortality monitoring rely heavily on household surveys, which are limited in providing recent estimate of mortality. Due to limitations in their sample size, typically household surveys provide estimate of child mortality on period 2 to 5 years in the past for national level estimates and ten years for sub-national level estimates. New approaches that can produce recent annual estimates are urgently needed [9,10].

This paper describes the use of HEWs in rural Ethiopia to monitor births and deaths within their communities, and assesses the accuracy and completeness of this approach for measuring mortality rates among children under-five on recent annual periods by comparing the results against mortality rates obtained from a large household survey. The study stems from the implementation of the “real-time” measurement of under-five mortality (RMM) project aimed to develop and test methods for measuring child mortality on recent periods no longer than twelve months that can be used at country level, and by partners to assess progress toward national and global goals for child survival.

## Methods

### Ethical approval

This study received ethical approval from the Johns Hopkins Bloomberg School of Public Health in the USA, and the Oromia Regional Health Bureau in Ethiopia. Given low literacy in the study population only oral consent was obtained from each study participant during study interviews. Oral consent was also used to ensure accuracy of information collected from each participant. Data collectors read approved oral consent forms translated in local languages to the each study participant. The forms explain the object of the study, why and how the participants have been selected for the study, privacy, confidentiality, voluntary participation and the possibility to stop the interview anytime without any sanction. The contact of the local principal investigator as well as the two institutional review boards mentioned above were provided to the participants in cases they have further questions. Interviews started only after the participants provided a verbal consent. The informed consent procedures were approved by the two institutional review boards mentioned above.

### Implementation of the RMM method

The RMM project was implemented in rural districts, locally referred at as woredas, in two zones (Jimma and West Hararghe) in collaboration with a local research center based in Addis Ababa, the Miz Hasab Research Center (MHRC). According to the 2007 population census, the two zones covered a total population of about 4.4 million (2.5 million in Jimma and 1.9 million in West Hararghe). Six percent of the Jimma population and 9% of the West Hararghe population is urban [11]. The two zones included a total of 31 rural woredas. Each woreda is subdivided in to kebeles. The RMM project was embedded within a larger evaluation of the integrated community case management (iCCM) program within the two zones that used a cluster randomized design with 31 woreda randomly assigned to intervention (16 woredas) and comparison (15 woredas) areas. Three kebeles were randomly selected in each of the 31 woredas, for a total of 93 kebeles. Selection of the three kebeles within each woreda was based on a systematic random sampling with probability proportionate to the size of the kebeles. However, accessibility to the kebele was also a determinant factor in the selection and kebeles that were unreachable were replaced by randomly selected the next kebele on the list.

Health officials at the Oromia Regional Health Bureau, the two zones, and each woreda were sensitized about the project before its launch. A formative research study was conducted in the selected kebeles to learn more about the existing HEW structure; this included their duties in terms of identification and reporting of pregnancies, births and deaths, the barriers and challenges to reporting, supervision issues, and the potential for alternative means to identify vital events. Following this research, a woreda focal point (WFP) person for the RMM project was identified in each woreda to coordinate the project.

Simple data extraction forms were developed to collect the monthly pregnancy, birth, and death events from the family folders at the health posts. A form was developed for each type of event and translated into the local language. MHRC worked with the regional and zonal health offices to train the HEWs and community volunteers from the selected kebeles, and their supervisors, on the proper maintenance of the extraction forms and family folders; WFPs were trained in supervision. Limited incentives were provided to the HEWs, volunteers and WFPs, such as backpacks, stationary, transport allowances and cell phones for the WFPs.

Data collection on pregnancies, births, and deaths at the community level started in December, 2011 and continued through March, 2013. The data collection process consisted of HEWs recording vital events in their existing family folders in the Jimma Zone, or kebele folders in West Hararghe (developed temporarily for the project), as they were identified throughout the month. At the end of the month, the HEWs transferred the information regarding pregnancies, births, and deaths they had recorded onto the extraction forms. These forms were submitted to the WFPs every month for review. MHRC research assistants visited each woreda monthly to collect the extraction forms, discuss data quality issues with the WFPs, and visit selected health posts. All unresolved data issues were followed-up through phone communications between the research assistants, the WFPs and the HEWs. All data forms were double-entered into a CSPro database and reconciled [12].

Two interim assessments of the quality of data reported were conducted during the course of the implementation. We describe and report on these interim assessments in S1 Appendix, pages 17–21.

### Validation of the RMM method

We used the endline household mortality survey data collected as part of the larger evaluation to validate data reported by the HEWs over a twelve month-period. Within the endline survey, we collected full birth histories from women of reproductive age [10,13]. See S1 Appendix (pages 10–23) for a data quality assessment of the endline validation data.

We calculated that a sample size of at least 26,300 households would be required to validate a one-year mortality rate using an equivalence test with 20% tolerance margin between the mortality rate from the HEW data and that from the household survey, and 80% power. However, for the larger evaluation, a sample size of 28,000 households was required. Thus the endline survey included a sample of 28,000 households, of which 27,872 were successfully reached during the survey. In total, 26,791 women aged 15–49 were successfully interviewed during the survey. The survey was conducted between February and May of 2013, and used a stratified two-stage cluster sampling design with stratification by woreda. The 2007 population census’s frame of enumeration area (EAs) was updated in terms of the distribution of the EAs by woreda and used as the primary sampling unit. The EAs were selected using systematic random sampling with probability proportionate to size. All inhabited households in each selected cluster were listed before a sample of about 35 households was randomly selected using systematic sampling with equal probability. Quality assessment of the data collected through assessment of the distribution of births and deaths over time, sex ratio at birth, and age distribution of women suggested good data quality (see S2 Appendix, pages 12–24). Household and women’s response rate was over 99%.

### Analysis

The validation analysis involved two components: (i) an evaluation of the completeness of birth and death information reported by HEWs and the assessment of age-related patterns of deaths, and (ii) a comparison of under-five, infant and neonatal mortality rates constructed from the HEW data with those estimated from the endline survey for 12-month periods. Data on births and under-five deaths reported by HEWs in selected West Hararghe and Jimma kebeles for the period of January, 2012 to March, 2013 were included in the validation analysis. We conducted the analysis on two rolling periods of twelve months: January to December, 2012 and April, 2012 to March, 2013.

To evaluate the completeness of births and under-five deaths documented by the HEWs, we estimated the expected number of births and under-five deaths for periods of 12-months. We estimated the crude birth rate and the under-five mortality rate directly from the endline validation survey. To estimate the expected number of births, we multiplied the crude birth rate for each annual period by the total population size of the two study zones. The expected number of under-five deaths was calculated similarly, by multiplying the under-five mortality rate estimated from the validation survey by the expected number of births. We examined completeness of HEW-reported births and under-five deaths reporting by analyzing the ratio of total number of births and under-five deaths documented by HEWs to the expected number estimated from the endline validation survey.

When comparing HEW-based mortality rates to those derived from the endline validation survey, we calculated the mortality rates in the same way to ensure direct comparability. Standard errors and confidence intervals were estimated using the jackknife resampling technique [14]. We used the Delta method to estimate the standard error of the ratio of the two sets of mortality rates [15]. We used an arbitrary tolerance margin of 20% of the survey mortality rate. We rejected the hypothesis of equivalence between the two mortality rates if the upper bound of the 95% confidence interval of ratio was less than 0.80 –concluding that the HEW-based data significantly under-estimated the under-five mortality rate. Similarly, if the lower bound of the 95% confidence interval of the ratio was greater than 1.20, we concluded that the HEW-based data significantly over-estimated the under-five mortality rate. All data analyses were conducted in Stata 13.0 and R statistical package [16, 17].

## Results

### Reporting of data on births and deaths by HEWs

**Fig 1:**
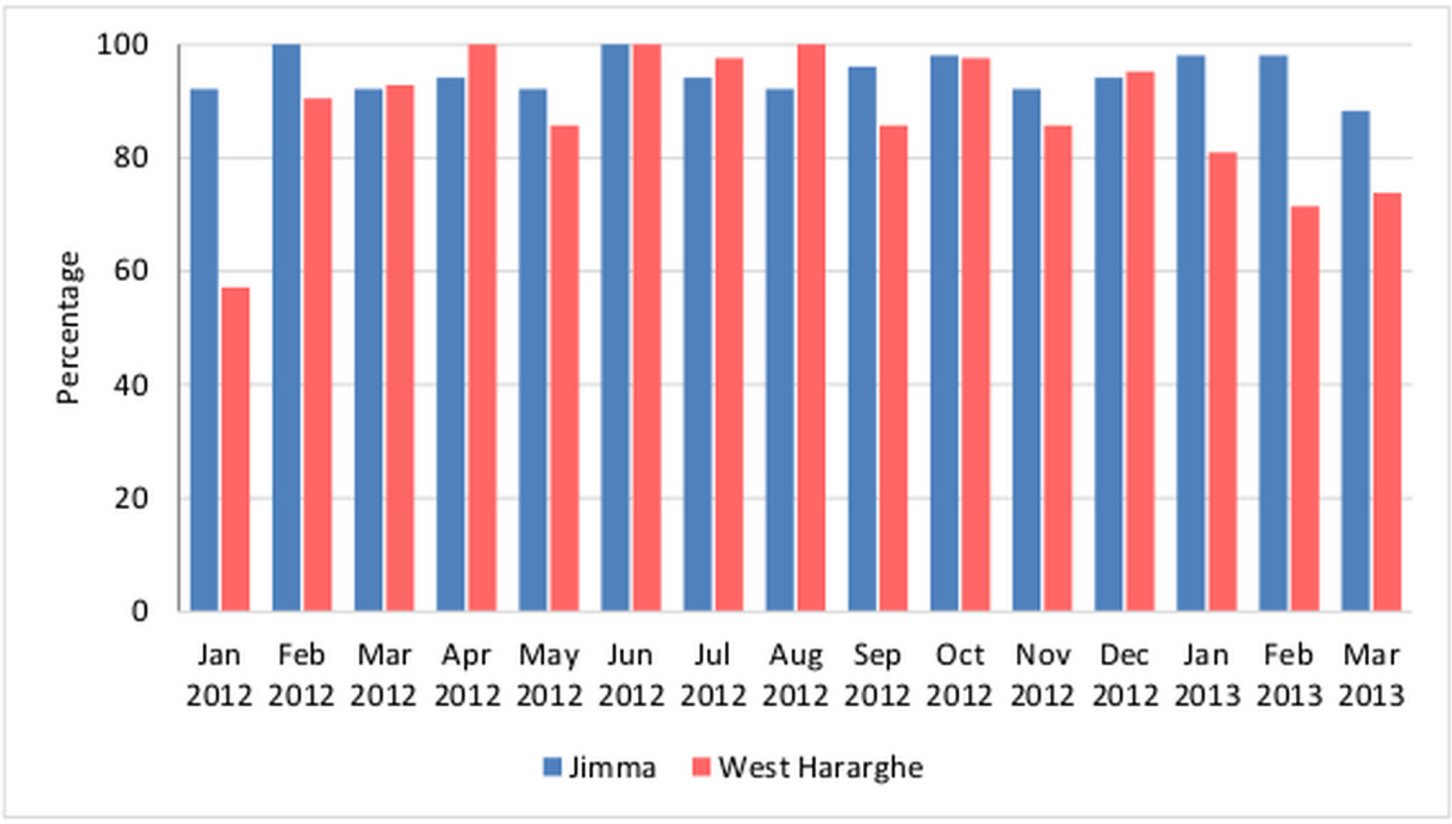
Percentage of health posts reporting data on timely basis by zone, (51 health posts in Jimma and 42 in West Hararghe), January 2012–March 2013.

Fig 1 shows health posts that have reported timely data, by month. The level of timely reporting of monthly extraction data was over 80% in both zones, except for February and March, 2013 in West Hararghe. The timely reporting in West Hararghe was more erratic than in Jimma, likely due to distance and difficult terrain. In total, the HEWs reported 5,860 live births in the 15-month validation period, 2,844 in Jimma, and 3,016 in West Hararghe. HEWs reported 285 under-five deaths in the 15-month reporting period, of which 54% (153) were neonatal. An almost equal number of under-five deaths and neonatal deaths were reported in Jimma and West Hararghe (See S2 Appendix pages 4–5).

### Completeness of births and under-five deaths reported by HEWs

In Table 1, we compare the total number of births and under-five deaths reported by HEWs to the expected number of births and under-five deaths based on the validation survey estimates and the population size estimates for Jimma and West Hararghe. The ratios of births and deaths documented by HEWs to the expected number of births and deaths, respectively, based on the survey estimates, were consistently low in both zones. The endline validation survey estimated approximately three times as many births and four to five times as many deaths as was documented by the HEW data collection process. Thus, although both events were under-reported, deaths appear more under-reported than births. Based on the household survey, HEWs reported only 30.4% of all expected births and 21% of expected under-five deaths from April, 2012 to March, 2013. Deaths were more severely under-reported than births in Jimma (30.6% versus 19.1%) compared to West Hararghe, where the difference in under-reporting between both events is small (29.5% versus 23.6%) for the period of April, 2012 to March, 2013.

**Table 1:** Comparison of expected births and deaths (based on endline survey) and reported births and deaths by HEWs, for 12-month validation periods, January, 2012 to March, 2013.

Annual Periods	Estimated Population of RMM kebeles	Estimated Crude Birth Rate (Endline Survey, per 1,000)	U5MR (Endline Survey, per 1,000)	Births			Under Five Deaths		
				Expected No. Births	No. Births Reported by HEWs	Ratio of Births reported by HEWs to Expected Births (%)	Expected No. of U5 Deaths	No. of U5 deaths reported by HEWs	Ratio of U5 reported by HEWs to Expected U5 deaths (%)
**JIMMA**									
Jan 2012—Dec 2012	285559	27.2	84.6	7767	2237	28.8	657	124	18.9
Apr 2012—Mar 2013	285559	26.4	81.2	7539	2310	30.6	612	117	19.1
**WEST HARARGHE**									
Jan 2012—Dec 2012	223836	40.9	53.9	9155	2724	29.8	493	132	26.7
Apr 2012—Mar 2013	223836	40.1	54.2	8976	2652	29.5	486	115	23.6
**TOTAL**									
Jan 2012—Dec 2012	509395	32.7	69.1	16657	4962	29.8	1151	256	22.3
Apr 2012—Mar 2013	509395	32.0	67.4	16301	4962	30.4	1099	231	21.1

### Sex and age patterns of births and deaths reported by HEWs

Table 2 presents sex-ratios at births for births reported by HEWs and those identified through the household survey for twelve month periods between January, 2012 to December, 2012 and April, 2012 to March, 2013. In most national populations, ratios of male versus female live births are expected to be between 102 and 107 males per 100 females [18]. Across the two zones and periods, the sex ratios at birth are approximately 100 male births to 100 female births and consistent between data collected by HEWs and by the household survey.

**Table 2:** Sex-ratio at birth, ratios of neonatal to infant deaths and infant to under-five deaths documented by HEWs & endline survey for 12-month validation periods, January, 2012 to March, 2013.

Annual Periods	Sex Ratio at Birth (%)		Ratio of Neonatal to Infant deaths (%)		Ratio of Neonatal to under-five deaths (%)		Ratio of Infant to under-five deaths (%)	
	HEW Reporting	Endline Survey	HEW Reporting	Endline Survey	HEW Reporting	Endline Survey	HEW Reporting	Endline Survey
**JIMMA**								
Jan 2012—Dec 2012	100.7	99.2	65.0	60.2	47.4	40.3	72.9	66.9
Apr 2012—Mar 2013	101.0	101.4	65.6	63.8	50.1	41.9	76.4	65.6
**WEST HARARGHE**								
Jan 2012—Dec 2012	100.2	102.4	66.8	56.6	53.3	38.8	79.8	68.6
Apr 2012—Mar 2013	98.6	97.9	69.1	62.5	54.5	46.4	78.9	74.3
**TOTAL**								
Jan 2012—Dec 2012	100.4	100.6	66.0	58.8	50.5	39.7	76.5	67.6
Apr 2012—Mar 2013	99.7	99.8	67.3	63.2	52.2	43.7	77.6	69.2

Table 2 also shows the proportion of infant deaths that occurred in the neonatal period, the proportion of under-five deaths that occurred in the first month of life, and the proportion of under-five deaths that occurred in the first year of life. The ratios observed based on HEW-based reporting in Jimma and West Hararghe appears consistently higher than the corresponding ratios computed from the household survey data. During 2012, half of under-five deaths (50.5%) reported by HEWs were neonatal deaths, two-third of infant deaths (66.0%) were neonatal, and over three-quarters of under-five deaths (76.5%) occurred during infancy. These proportions were estimated at 58.8%, 39.7% and 67.6%, respectively, from the household survey data.

### Accuracy of child mortality rates from data reported by HEWs

Table 3 shows that the HEW-based under-five mortality rate estimates were approximately 70% of the magnitude of those estimated by the endline survey for both time periods. The ratios of HEW-based to endline survey under-five mortality rates for West Hararghe (80–90%) were notably higher than for Jimma (approximately 66%) for both time periods. Since the under-reporting of births and deaths was of about similar magnitude for West Hararghe across the two validation periods, the HEW-based under-five mortality rate estimates are close to those estimated by the endline survey. This stems from the under-registration of birth events and the under-registration of death events by HEWs approximately cancelling each other out when calculating under-five mortality rates. This was not the case for Jimma where the degree of under-reporting of deaths by HEWs is higher than that of births, resulting in substantially higher under-estimation of the under-five mortality rate. The difference in the findings between Jimma and West Hararghe suggests non-stability in the degree of under-reporting of vital events by HEWs in different contexts. In both zones, however, the upper bound of the 95% confidence intervals of the ratio of the mortality rates suggests that the rates from the HEW-collected data are equivalent to those measured from the validation survey at a 20% tolerance level.

**Table 3:** Under-five mortality rate estimates based on HEW data & expected under-five mortality rate estimates based on endline survey for 12-month validation periods, January, 2012 to March, 2013.

Annual Periods	Under-Five Mortality Rate (per 1,000)					
	HEW data		Endline Survey		Ratio of Mortality Rates, HEW Data to Endline Survey	
	Rate	95% CI	Rate	95% CI	Ratio	95% CI
**JIMMA**						
Jan 2012—Dec 2012	55.0	(47.1, 62.9)	84.6	(78.1, 91.1)	65.7	(50.2, 80.9)
Apr 2012—Mar 2013	50.5	(43.5, 57.5)	81.2	(74.7, 87.6)	67.2	(50.0, 84.4)
**WEST HARARGHE**						
Jan 2012—Dec 2012	48.4	(41.5, 55.3)	53.9	(49.1, 58.7)	89.8	(72.8,106.8)
Apr 2012—Mar 2013	43.2	(36.7, 49.7)	54.2	(49.4, 59.0)	79.7	(61.2, 98.2)
**TOTAL**						
Jan 2012—Dec 2012	51.7	(46.4, 57.0)	69.1	(65.1, 73.1)	74.8	(52.2, 97.4)
Apr 2012—Mar 2013	46.6	(42.8, 51.4)	67.4	(63.4, 71.4)	69.1	(44.1, 94.1)

Comparison of neonatal mortality rates and infant mortality rates estimated from the HEW data with those estimated from the endline survey shows similar results to those for under-five mortality rates (see Tables 4 and 5). Despite substantial under-registration of both birth events and neonatal and infant death events by HEWs relative to the endline survey, the HEW-based neonatal and infant mortality rates appeared to also be equivalent to the rates from the endline survey in West Hararghe, judging from the 95% confidence intervals of the ratios.

**Table 4:** Infant Mortality Rate estimates based on HEW data & expected Infant Mortality rate estimates based on endline survey for 12-month validation periods, January 2012 to March, 2013.

Annual Periods	Infant Mortality Rate (per 1,000)					
	HEW data		Endline Survey		Ratio of Mortality Rates, HEW Data to Endline Survey	
	Rate	95% CI	Rate	95% CI	Ratio	95% CI
**JIMMA**						
Jan 2012—Dec 2012	40.5	(30.6, 50.4)	56.6	(51.3, 61.9)	71.6	(48.8, 79.6)
Apr 2012—Mar 2013	38.6	(31.6, 45.6)	53.3	(48.1, 58.5)	72.4	(38.7, 73.2)
**WEST HARARGHE**						
Jan 2012—Dec 2012	38.7	(31.7, 45.7)	37.0	(33.0, 40.9)	104.6	(76.2, 111.0)
Apr 2012—Mar 2013	34.1	(27.3, 59.4)	40.2	(36.1, 44.4)	84.8	(52.3, 90.0)
**TOTAL**						
Jan 2012—Dec 2012	39.5	(34.3 44.7)	46.7	(43.4, 50.0)	84.6	(52.9, 98.7)
Apr 2012—Mar 2013	36.2	(31.2, 41.2)	46.7	(43.3, 50.0)	77.5	(37.2, 87.8)

**Table 5:** Neonatal mortality rate estimates based on HEW data & expected neonatal mortality rate estimates based on endline survey for 12-month validation periods, January 2012 –March, 2013.

Annual Periods	Neonatal Mortality Rate (per 1,000)					
	HEW data		Endline Survey		Ratio of Mortality Rates, HEW Data to Endline Survey	
	Rate	95% CI	Rate	95% CI	Ratio	95% CI
**JIMMA**						
Jan 2012—Dec 2012	26.3	(19.5, 33.1)	34.0	(30.0, 38.2)	77.4	(59.3, 95.5)
Apr 2012—Mar 2013	25.3	(19.0, 31.6)	34.0	(29.8, 38.1)	74.4	(50.9, 93.9)
**WEST HARARGHE**						
Jan 2012—Dec 2012	25.8	(19.3, 31.9)	21.0	(18.0, 23.9)	122.9	(102.7, 143.1)
Apr 2012—Mar 2013	23.6	(17.9, 29.3)	25.2	(21.9, 28.5)	93.7	(71.4, 109.6)
**TOTAL**						
Jan 2012—Dec 2012	26.1	(22.5, 30.7)	27.4	(25.0, 30.0)	95.3	(68.7, 121.9)
Apr 2012—Mar 2013	24.4	(20.2, 28.6)	29.5	(26.8, 32.1)	82.7	(66.2, 111.2)

## Discussion

In the past decade, Ethiopia has expanded and strengthened its community-based health system program through training and deployment of health extension workers in each of the 17,000 kebeles in the countries. This provides a unique opportunity to not only extend health services to a large population, but also to build up a system of community monitoring of vital events, linking health system information to the Civil Registration and Vital Statistics System (CRVS) that the country has committed to build by the year 2020. Furthermore, current approaches for monitoring child mortality rely heavily on household surveys, which are limited in providing recent national estimates due to limited sample size [9]. Health facility data are generally of limited quality to support child mortality measurement [19]. The current study is the first in Ethiopia that assesses the feasibility and validity of training HEWs to identify and report on births and deaths within their communities as a strategy for monitoring changes in mortality among children under five, in real-time.

The validation results pointed to the feasibility of using HEWs to routinely report on vital events. However, completeness of number of events identified and reported was very low and variable across the two zones. Compared to household survey estimates, HEWs reported only about 30% of births and 21% of under-five deaths occurring in their communities over a twelve-month period from April, 2012 to March, 2013. The resulting mortality rates were also under-estimated due to differential under-reporting of births and deaths. The under-five mortality rate was under-estimated by around 30%, infant mortality rate by 23%, and neonatal mortality by 17%. We observed that HEWs reported disproportionately higher number of deaths among the very young infants than among the older children. Differences in the level of completeness and accuracy between Jimma and West Hararghe suggested that the approach was not stable across different contexts. These findings suggest that birth and death data reported by HEWs are not complete enough to support a system for monitoring changes in childhood mortality. These results are indicative of the considerable challenges of real-time vital events identification in remote, low population-density areas such as rural Ethiopia.

This first test indicated a need to understand and improve the coverage of vital events reporting, given the considerable under-reporting of births and child deaths by HEWs. It is important to understand whether HEW-based vital events reporting is subject to considerable selection biases relative to full birth history reporting via retrospective surveys. For example, do HEWs tend to only document events close to their home villages or health posts? Or is the under-reporting by HEWs approximately randomly distributed across time and space or at least predictable based on proxy indicators such as population density, seasonality, and other observable traits that can be used to reliably adjust HEW-based events reporting for under-registration?

With regards to distribution of births reported by sex, the sex-ratios were around 100 males per 100 males and did not indicate substantial under-report of a particular sex. In most national populations, ratios of male to female live births are expected to be between 102 and 107 males for every 100 females [8]. The differences here were not large enough to suspect any sex selective abortion or substantial under-reporting of male births.

Interestingly however, HEWs appeared to have picked-up a disproportionally higher number of early deaths (neonatal and infant) relative to later deaths compared to full birth history-based mortality rates. Although the distribution pattern of deaths by age appeared consistent with distribution observed in typical populations, the ratios observed from the HEW-reported data were higher than observed in other rural populations in low-resource settings. This pattern suggested that HEWs were reporting disproportionately more deaths among the very young than the older children. In fact, the ratio of early neonatal deaths to neonatal deaths observed from HEW-reported data was 86%, much higher than the typical range of 70–80% observed in low-resource settings [20]. Further studies should also focus on understanding this pattern of reporting.

Our findings are subject to some limitations. First, our validation of vital events reporting by HEWs and the resulting child mortality rate measurements were limited to two zones of Oromia. Thus, it is difficult to generalize these findings to other regions of Ethiopia with substantially different context and culture. Second, our analysis and validation did not take into account the possible effect of migration on HEW reporting of vital events or validity of the full birth history. Third, the full birth histories that we collected during the endline survey may have been affected by recall errors. However, we expect such error to be small given the short recall and reference period. Finally, there may be potential conflict interest when community health workers, who are responsible for preventing adverse outcomes such as deaths, are requested to report on these outcomes. However, we do not believe that, if the conflict was real, it was the cause of the high level of under-report of deaths observed. This is because births were also highly under-reported. Studies exploring the possibility of this potential conflict would inform the setup of monitoring systems based on health worker reporting, including the reporting of adverse outcomes in health management information systems.

The modest nature of the validation results of this community-based method, especially for events, are likely due to a number of factors, including the considerable workload (which often includes non health-related activities) of HEWs, the challenges of supervising HEWs in remote areas, and the challenges of covering wide areas where transportation and the communications infrastructure are weak. It is critical to also understand the functional relationship between the HEWs and community volunteers where identification of vital events are concerned. However, our interim data quality assessments of the HEW-based reporting indicated high levels of content accuracy in the vital events that were reported. This indicates that the basic foundation of HEW-based vital events reporting is strong, and suggests that as the government of Ethiopia scales up the number of HEWs in rural areas of the country, efforts to strengthen and extend this solid foundation will be important. As Ethiopia strives to develop and expand a CRVS, with a goal of reaching a reasonable coverage by 2020, community health workers and community volunteers constitute key actors that connect with communities and will be critical in its success. The current draft plan presented by Ethiopia for CRVS does not seem to implicate HEWs, possibly because of their already full job description. However community-based health workers are essential for building a strongly linked functioning health information system and CRVS.

## Supporting Information

S1 Appendix Project report: community-based real-time mortality monitoring in Ethiopia. (PDF)

S2 Appendix Appendices to RMM project report in Ethiopia. (PDF)
