# Supplementary material for: Using Health Extension Workers for Monitoring Child Mortality in Real-Time: Validation against Household Survey Data in Rural Ethiopia
Source: PLoS One. 2015 Nov 25;10(11):e0126909. doi: 10.1371/journal.pone.0126909 (PMC4659609; doi:10.1371/journal.pone.0126909)
Supplement: S1 Appendix — (PDF) [file pone.0126909.s001.pdf]

S1 APPENDIX

PROJECT REPORT

COMMUNITY-BASED REAL-TIME MORTALITY MONITORING IN ETHIOPIA.

TABLE OF CONTENTS

I. INTRODUCTION AND BACKGROUND 1
1.1. Introduction to the overall RMM project 1
1.1.1. *Global context, rationale and objectives of the RMM Project* 1
1.1.2. *RMM design and implementation* 2
1.2. Background and context for RMM in Ethiopia 3
1.2.1. *Background and country setting* 3
1.2.2. *The Health Extension Program (HEP) and Health Extension Workers (HEWs)* 4
1.2.3. *Design of the evaluation of Integrated Community Case Management (ICCM) in Ethiopia* 5
2. COMMUNITY-BASED RMM METHOD 6
2.1. Objectives 6
2.2. Design 7
2.3. Formative research 7
2.3.1. *Objectives of the formative research* 7
2.3.1 *Formative research methods* 7
2.3.2. *Formative research results* 8
2.3.3. *Formative research conclusions* 8
2.4 Ethical clearance 9
2.5 Implementation of the community-based RMM method 9
2.5.1 *Selection and training of the Miz-Hasab RMM team* 9
2.5.2. *Sampling* 10
2.5.3. *Implementation* 11
2.6 Interim data quality assessment I (DQA I) 17
2.6.1. *Background and objectives* 17
2.6.2. *Methods* 17
2.6.3. *Findings* 18
2.6.4. *Conclusions* 19
2.7 Interim data quality assessment II (DQA II) 19
2.7.1. *Background and objectives* 19
2.7.2. *Methods* 19
2.7.3. *Findings* 20
2.7.4. *Conclusions* 21
2.8. Endline validation survey 22
2.8.1. *Background and objectives* 22
2.8.2. *Sampling* 22
2.8.3. *Survey response rate* 23
2.9. Validation Analysis 23

2.9.1. *Community-based RMM Method* 23
2.9.2. *Endline validation survey* 23
2.9.3. *Validation analysis* 24
2.10. Results 24
2.10.1. *RMM community-based method* 24
2.10.2. *RMM community-based validation* 31
References 36

List of Acronyms

BMGF	Bill and Melinda Gates Foundation
CBR	Crude Birth Rate
CBRHA	Community Based Reproductive Health Associate
CHW	Community Health Workers
CIDA	Canadian International Development Agency
CV	Community volunteer
DA	District Assembly
DFATD	Department of Foreign Affairs, Trade, and Development
DHS	Demographic and Health Survey
DMHT	District Management Health Team
HMIS	Health Management Information System
HEW	Health Extension Worker
IET	Independent Evaluation Team
IIP-JHU	Institute for International Programs- Johns Hopkins University
IHSS	Integrated Health System Strengthening, UNICEF
IMCI	Integrated management of childhood illness
IRB	Institutional Review Board
IT	Information technology
JHSPH	Johns Hopkins Bloomberg School of Public Health
LiST	Lives Saved Tool
MDG	Millennium Development Goals
MICS	Multiple Indicator Cluster Survey
MHRC	Miz-Hasab Research Center
MNCH	Maternal, Newborn, and Child Health
MOH	Ministry of Health
NORAD	Norwegian Agency for Development Cooperation
NSO	National Statistical Office
PMNCH	Partnership for Maternal, Neonatal and Child Health
PSU	Primary Sampling Unit
QoC	Quality of Care
RMM	Real-Time Mortality Monitoring
SPSS	Statistical Package for the Social Sciences
SMS	Short Message Service
SSU	Secondary Sampling Unit
TA	Traditional Authority
TBA	Traditional birth attendant
TRAction	Translating Research into Action
UNICEF	United Nations Children's Education Fund
UNFPA	United Nations Population Fund
USAID	United States Agency for International Development
VHC	Village Health Committee
VHR	Village Health Register
WFP	Woreda focal person
WHO	World Health Organization

I. INTRODUCTION AND BACKGROUND

1.1. Introduction to the overall RMM project

The "Real-time" Monitoring of Under-Five Mortality" (RMM) project aims to develop and test methods for measuring child deaths that can be used at country level and by partners to assess progress toward national and global goals for child survival. The work is supported by the Canadian Department of Foreign Affairs, Trade and Development (DFATD)[*] and implemented by the Institute for International Programs of the Johns Hopkins University (IIP-JHU) working in collaboration with research institutions in Ethiopia, Ghana, Malawi, Mali and Niger. RMM is one component of the Catalytic Initiative to Save a Million Lives (CI),[1] a ten-year, multi-donor initiative led by Canada as part of its Africa Health Systems Initiative that aims to accelerate progress in child survival.

In this chapter we provide a brief review of the overall project, sufficient to allow readers who are interested only in the Ethiopian experience to understand the larger context. A full description of the origins, design and methods of the RMM project is available in the Overview chapter of this report. An in-depth assessment of the implications of the project findings is available in the final chapter, targeted to national and global policymakers interested in strengthening vital registration systems in low- and middle-income countries.

1.1.1. Global context, rationale and objectives of the RMM Project

The Millennium Development Goals (MDGs) were signed in 2000 by 189 countries.[2] The fourth MDG calls for a two-thirds reduction in under-five mortality between 1990 and 2015. Countries and their development partners are responsible not only for achieving this target, but for measuring their progress at regular intervals. Most low- and middle-income countries rely on nationally representative surveys conducted by the USAID-supported Demographic and Health Survey program[3] or the UNICEF-supported Multiple Indicator Cluster Survey program.[4] Both DHS and MICS surveys produce national estimates of under-five mortality for periods three to five years in the past. Estimates for smaller administrative units, such as regions, provinces, or zones, must rely on even longer recall periods and can therefore produce estimates for up to 10 years in the past.

Beginning in 2007, Canada responded to these challenges by inviting UNICEF, WHO, the World Bank and major partners in child survival including the United States Agency for International Development (USAID) and the Bill & Melinda Gates Foundation to join them as part of the CI, with the aim of accelerating coverage of proven child survival interventions – especially through integrated community-based delivery of case management for childhood pneumonia, diarrhea and malaria, or CCM -- in selected countries in sub-Saharan Africa. This led to a set of CI projects, including support from Canada to UNICEF to implement the Integrated Health Systems Strengthening project (CI/IHSS) in Ethiopia, Ghana, Malawi, Mali, Mozambique and Niger, and support from the Gates Foundation to the Partnership for Maternal, Newborn and Child Health to implement the Rapid Scale-Up for MNCH project (CI/RSU) in Burkina Faso, Malawi and Mozambique.[†]

---

[*] Prior to a restructuring in 2013, the project was supported by the Canadian International Development Agency (CIDA). We have used DFATD throughout the report.

[†] Lead responsibility for implementation of this project shifted in 2009 to WHO.

A founding principle of the CI was a commitment to produce regular estimates of under-five mortality that were based on measurement rather than modeling. Canada, in particular, anticipated and in many ways stimulated the current accountability agenda[5] by calling for the exploration of potential methods for estimating under-five mortality in "real-time", i.e. in recent periods of 12 months or less. The RMM project was developed to respond to this need, and where feasible to provide interim measurements of the CI-IHSS contributions to declines in under-five mortality in CI/IHSS countries.

The overall objectives of the RMM project are:

1. To develop, implement and assess alternative methods for producing and interpreting measurements of under-five mortality in large geographic areas at intervals no longer than 12 months, i.e. "in real-time".
2. Where feasible, to use proven RMM methods to produce evidence of the effectiveness of CI/IHSS activities and interventions in producing accelerations in mortality reduction over the course of the project.

We are also documenting the costs of implementing the community-based method in each setting.

The countries participating in RMM are Ethiopia, Ghana, Malawi, Mali and Niger. All project activities were conducted in ways that build sustainable capacity for program evaluation and/or the monitoring of vital events in local research institutions in each participating African country.

1.1.2. RMM design and implementation

We began the project with a consultative process to identify feasible, innovative approaches to RMM, involving international experts in mortality measurement and the tracking of vital events at community level.[6] Four categories of methods were defined: (1) tracking vital events at community level; (2) calibrating child deaths recorded in health facilities to all deaths; (3) rapid survey methods based on summary birth histories; and (4) strengthening birth and death registration systems. We then consulted with country stakeholders, including Ministries of Health and other partners, and assessed existing opportunities to develop and test these innovative approaches. In each country, we identified at least two promising approaches and implemented each for a period of at least twelve months. The resulting mortality data were then compared to results produced by a current best standard household survey or census, to determine the accuracy of the approach. Start-up and continuation costs were tracked over time for community-based methods. Figure 1 lists the potential RMM methods tested in each of the five country settings.

Table 1. Potential RMM methods tested in each participating country

Country	Vital event tracking at community level		Calibrating health facility data	Rapid HH surveys		Strengthened birth & death registration
	Paid worker	Unpaid worker		Cohort Change	Imputed	
Ethiopia	✓			✓	✓	
Ghana		✓			✓	✓
Malawi	✓		✓	✓	✓	
Mali		✓			✓	
Niger					✓	

1.2. Background and context for RMM in Ethiopia

1.2.1. Background and country setting

Ethiopia is a large East African country with about 1.1 million square kilometers, bordered by Djibouti, Eritrea, Sudan, South Sudan, Kenya and Somalia. With an estimated population of over 87 million[‡], the country is the second populous country in sub-Saharan Africa, behind Nigeria.[7] The country is mainly rural with over 80% of the population living in rural area making Ethiopia one of the least urbanized countries in the world. The country is administratively divided into eleven regions but over 80% of the population is concentrated in the Oromia, Amhara, and SNNP regions.

About 30% of Ethiopians live under the poverty level, but the country has made some recent economic progress with reported 9% growth in GPD in 2012 and a GDP per capita of 455.[8] There have also been Improvements in key demographic and health indicators. For example, total fertility rate stalled at around 5.5 children during the periods 1995-2000 and 2000-2005, before declining to 4.8 over the period 2006-2011. Fertility remains higher in rural than in urban areas (5.5 versus 2.6 children). Modern contraceptive prevalence among currently married women has doubled over the past five years, from 14% in 2005 to 27% in 2011.

Table 2. Demographic and economic characteristics of Ethiopia

Characteristics	Value	Source
Total population (in thousand)	87,952	CSA population projection (www.csa.gov.et)
Urban	16,676	
Rural	71,277	
Total Fertility Rate (TFR)	4.8	DHS 2011
Urban	2.6	
Rural	5.5	
Modern CPR	27	DHS 2011
GDP growth in 2012	8.7%	WDI
GDP per capita in 2012	455	WDI
Poverty headcount at national poverty line (% population living under poverty line) in 2011	29.6%	WDI
U5MR (2012)	68.3	IGME, 2013
Annual Rate of Reduction (ARR) in U5MR (1990-2012)	5.0%	IGME, 2013
Number of under-five deaths (2012)	205,000	IGME, 2013
Neonatal mortality rate (2012)	29	IGME, 2013
HIV prevalence rate among adults 15-49 (2011)	1.5%	DHS 2011

In terms of child health, Ethiopia faces a high burden of communicable infectious diseases and malnutrition. Sixty-six percent of deaths among children under-five are attributed to pneumonia, diarrhea, malaria and neonatal conditions (WHO/CHERG 2010). Child mortality declined steadily from 204 to 68 deaths per thousand live births between 1990 and 2013, with an estimated average annual rate of reduction of 5.0%, making Ethiopia one of the few African countries that are on track to achieve MDG4.[9] Neonatal mortality has also declined by 46%, from 54 to 29 deaths per thousand live births, over the same period. Stunting, an indicator of chronic malnutrition, has declined steadily from 58% in 2000 to 44% in 2011.

These improvements reflect the commitment of the country to improve the well-being of the Ethiopia population through concrete policies and programs. Faced with these poor health conditions in the early 1990s, Ethiopia adopted a national health policy and launched a twenty-year health sector development plan (HSDP) in 1997, organized into five-year strategic plans, to accelerate increases in access to health care and improve the health and sanitary conditions of the population. Ethiopia has participated in global initiatives including the Millennium Development Goals, the Global Fund, PEPFAR, etc. A key hallmark of the HSDP has been the bold strategic decision by the government in 2004 to achieve universal access to

[‡] Projection of the 2007 population county by Central Statistical Agency (CSA). The 2007 Population census estimated the total population at 73.8 million. www.csa.gov.et

health services through the posting of trained health extension workers (HEWs) in every rural kebele. The Government trained and deployed over 34,000 HEWs, initially to provide preventive and promotive health services, and later to also manage uncomplicated cases of childhood illnesses such as diarrhea and malaria. With the goal of reaching one health extension worker per 2500 population, drastic increases in construction of health posts has followed and by the end of the HSDP III in 2010, close to 15,000 health posts and 3000 health centers had been constructed.[10,11] Progress has also been achieved in financing for health, with an increase in per capita health expenditure from USD 7.14 in 2004/05 to 16.09 in 2007/08.

With support from partners, Ethiopia upgraded its existing health extension program (HEP) in 2010 to include treatment of pneumonia at community level and launched a new integrated community case management program with additional training of HEWs, an enhancement of supervision and monitoring and support in terms of essential drugs and commodities. The ICCM program was rolled out initially in 2010 in the four biggest regions of the country (Amhara, Oromia, SNNP and Tigray) before being expanded to other regions.[12] The implementation was planned to move rapidly to cover the entire regions except in Oromia, where it was phased in.

The Institute for International Programs (IIP-JHU) at Johns Hopkins Bloomberg School of Public Health was commissioned by the Canadian government and UNICEF to conduct an evaluation of the ICCM in Ethiopia. IIP-JHU, in collaboration with the Federal Ministry of Health (FMOH) and UNICEF, focused the evaluation in Oromia because the phased roll-out of ICCM implementation a rigorous design that included the areas with later ICCM roll-out as a comparison area. Within the Oromia region, the evaluation was conducted in Jimma and West Hararghe zones and within each of these zones, intervention and comparison woredas were randomly identified. The RMM approaches were implemented in these same areas.

IIP-JHU partnered with a local Ethiopian research institution (Miz Hasab Research Center, MHRC) for the implementation of RMM. MHRC was responsible for conducting a formative research to inform the design of the RMM, implementing the RMM method and assessing the quality of data generated.

1.2.2. The Health Extension Program (HEP) and Health Extension Workers (HEWs)

The health system structure in Ethiopia requires a health post in every kebele, covering approximately a population of 5,000 people. To date, the FMOH has trained and deployed 34,000 HEWs in 16,000 health posts serving a rural population of approximately seven million.[13] From 2010-2013, the FMOH retrained approximately 27,000 to manage uncomplicated cases of malaria (with ACT), diarrhea (with ORS and zinc), pneumonia (with cotrimoxazole), and severe acute malnutrition with ready-to-use therapeutic food (RUTF) as part of the ICCM program.[13]

The HEWs are paid, female government health workers who recieve a 12 month training on disease prevention and health promotion. There is no age limit for the recruitment of HEWs. Most have completed 10th grade and some have completed high school. Two HEWs are generally assigned to each health post, where they provide case management of childhood illnesses and conduct frequent visits in their communities to identify and train model families, provide preventive and safe environment messages. HEWs are expected to spend the majority of their time visiting families in their homes and performing outreach programs in the community.

The HEWs work with a group of several volunteers in each kebele. Within each kebele, households are organized into teams called “garees”. Each “garee” includes about 30 households and appoints a leader, who in most cases is also a volunteer in the community. HEWs meet regularly with volunteers in their

community (frequency of meetings is generally every two or three weeks) to discuss community issues and identify any vital events in the community. The HEWs generally maintain a logbook at their post that record pregnancies, births and deaths of children.

The FMOH leads the health sector including ICCM programs through policymaking and coordination while supporting health post construction, training and deploying of HEWs and covering salary and supervision costs. Implementation partner include Save the Children international, John Snow Inc. /Last 10 Kilometers (JSI/L10K), International Refugee Committee (IRC), Merlin (Emergency), African Medical and Research Foundation (AMREF), and United States Agency for International Development/Integrated Family Health Program IFHP (USAID/IFHP) and United Nations Children Fund (UNICEF). (REF: Institute for International Programs at Johns Hopkins University and Save the Children. Translating Research into Action (TRAction) Implementation Research Embedded in Integrated Community Case Management (CCM) Program: Ethiopia desk review 2014).

1.2.3. Design of the evaluation of Integrated Community Case Management (ICCM) in Ethiopia

We describe this design here because it is closely linked with the activities of the RMM project. The prospective evaluation used a cluster randomized design with stratification by zone, within which woredas were randomly assigned to intervention and comparison arms using an adapted, restricted randomization process, balanced by the presence of malaria, food security and geographic location. The Oromia Regional Health Bureau (ORHB) had committed to at least an 18-month phase-in between the identified intervention and comparison areas in these two areas. Intervention woredas implemented ICCM while the comparison woredas continued to offer routine HEP services. Urban areas were excluded because the urban HEP is a recent addition and implemented differently. A total of 16 woredas were assigned to intervention area and 15 to comparison area.

<u>Figure 1.</u> Map of evaluation zones in Oromia region, Ethiopia

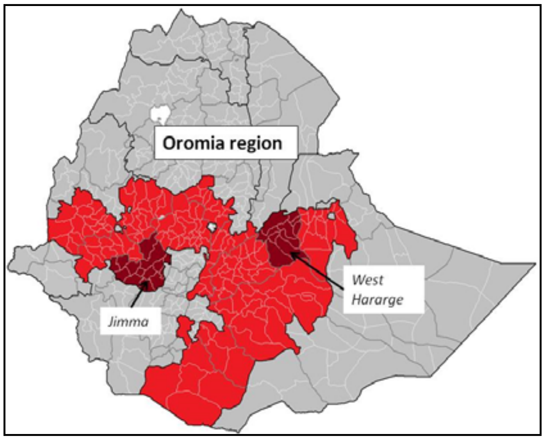

<u>The evaluation area.</u> The evaluation was conducted in Jimma and West Hararghe zones (figure 1). These two zones were selected based on the following criteria:

- Population size: Both are large zones with an adequate population size and a large enough number of woredas to designate woreda-level intervention and comparison areas.
- Partner strength: Strong child health partners that were expected to accelerate the scale-up of HEP+ICCM. The Last 10 Kilometers (L10K) is a Gates Foundation-supported organization working in CCM in Jimma zone, while the Integrated Family Health Program (IFHP) is a USAID-supported organization implementing in West Hararghe.
- Geographic location: Jimma is in western Oromia, while West Hararghe is in the eastern part of the region. Both zones are approximately the same distance from Addis Ababa.

Table 3. Evaluation zones and interventions and comparison woredas

Jimma Zone	West Hararghe Zone
INTERVENTION WOREDAS	
Chora Botor	Boke
Gera	Chiro
Goma	Doba
Kersa	Gemechis
Limu Kosa	Guba Koricha
Mana	Hawi Gudina
Shebe Senbo	Oda Bultum
Omo Nada	
Setema	
COMPARISON WOREDAS	
Dedo	Anchar
Gumay	Burka dimtu
Limu Seka	Daro Labu
Nono Benja	Habro
Seka Chokorsa	Mesela
Sokoru	Mieso
Tiro Afeta	Tulo
Sigamo	

Building on the evaluation, RMM-Ethiopia team targeted the two zones and woredas where the evaluation was conducted. We focused the RMM in the two zones in order to use the endline mortality survey that was planned for the independent evaluation for validation of the RMM mortality results. According to the 2007 population census, the two zones cover a total population of about 4.4 million (2.5 million in Jimma and 1.9 million in West Hararghe). Six percent of the Jimma population and 9% of the West Hararghe population is urban.[14]

2. COMMUNITY-BASED RMM METHOD

2.1. Objectives

The overall objective of the community-based RMM method in Ethiopia is to test whether HEW records of births and deaths can be used to accurately monitor changes in under-five mortality in real-time. More specifically, the following activities were implemented as part of the RMM project:

1. Formative research;
2. Implementation of an RMM approach on HEWs' recording of births and deaths in their communities;
3. Assessments of the implementation and consistency, accuracy, and completeness of the RMM data at three and six months after start-up; and

4. Validation of the accuracy of the RMM method in measuring annual under-five mortality against mortality rates produced by an endline survey with full birth history carried out jointly with the ICCM evaluation.

2.2. Design

HEWs are stationed at health posts in each kebele, and are required by the Government to maintain "family folders" in which they record information on each family in their communities, including a listing of all family members. Most HEWs receive help in these tasks and in the identification of other health issues and pregnancies from community volunteers (CVs). These volunteers are usually "garee" (team) leaders in their communities, with each garee including about thirty households.

The community-based RMM method in Ethiopia relies on this existing system of recording of data at community level, working with randomly selected health posts. Rural kebeles were randomly selected from each woreda in intervention and comparison areas within the two evaluation zones (see below number of kebele selected in each woreda). A woreda focal point person (WFP) was identified at the Woreda Health Office level and trained to supervise the selected HEWs and collect the monthly records of vital events. Simple data extraction forms were developed to collect the monthly events from the health posts. HEWs were trained to record data on these simple forms and store them in the family folders. At the end of the month, the HEWs utilized a data extraction form to extract the overall reported pregnancy, birth and death data and report them to the WFP. MHRC worked with the regional and zonal office to train the selected HEWs and their supervisors on the proper maintenance of the forms and family folders, especially the recording of births and deaths in their communities and proper extraction of pregnancies, births and deaths from these folders onto the extraction forms.

2.3. Formative research

2.3.1. Objectives of the formative research

Prior to implementing the community-based RMM method, formative research was conducted to:

1. Learn about the current status of HEWs' roles in identifying and reporting vital events within their communities;

2. Generate the information needed to develop clear and effective procedures for identifying and recording of vital events (pregnancies, births and child deaths) by HEWs in their communities; and

3. Identify other possible alternatives for collecting data on vital events at community level.

In this section we provide a summary of the methods and key results of the formative research study. A full report is available upon request.

2.3.1 Formative research methods

The study was conducted in Jimma and West Hararghe zones. Two woreda were purposively selected in each zone and within each woreda, two kebele were purposively selected, making a total of 8 kebeles included in the study. Availability of health posts, health extension workers (HEWs) and accessibility were factors that determined the selection of woreda and kebeles.

Data were collected in July and August 2011 by two teams. Each team consisted of two trained research assistants and a supervisor. In total, 47 individual in-depth interviews and 24 FGDs were conducted in the two zones. The in-depth interviews were conducted with public health officials, community leaders, and health extension workers at the zonal, woreda and kebele levels. These were followed by the Focus Group Discussion (FGDs) in each selected kebele involving groups of parents with children under five years of age and community volunteers. Each FDG consisted of 8 to 10 participants. Kebele officials and HEWs helped identify the study participants. Interviews and discussions were conducted in the local language (Oromifa). Interview guides were used, discussions and interviews were taped recorded, and notes were taken of key points. Recorded interviews were transcribed analysis.

2.3.2. Formative research results

Roles of HEWs: Most respondents in both the interviews and FGDs were well acquainted with the HEWs and agreed that they had contributed to improving the health status of their community. Specific examples of HEWs' activities cited by respondents included distribution of mosquito nets, public education on prevention of standing water, family planning counseling, environmental hygiene including the importance of building latrines, handwashing, vaccination, nutritional supplement for malnutrition in children, and follow-up with patients in their homes.

Status of recording of vital events: Many respondents knew that HEWs collected vital statistics data in their rural communities. However, the interviews with zonal officials showed that there was no specific system in place for collecting vital events. Respondents reported that some information on pregnant women and newborns were collected on an *ad-hoc* manner when HEWs went to households to provide other services.

The responses indicated that no standard system was in place for collecting vital events at community level. However, some information on pregnant women and children were collected as part of the HEP program requirement for HEWs to provide services to pregnant women throughout their term- at least 3 or 4 times during the year. As a result, they were able to obtain data on births when providing vaccination services to mothers and children. Most deaths were not recorded.

Current status of the "family folders": The study indicated that family folders were being used Jimma zone but had not yet been implemented in West Hararghe zone. In Jimma, many respondents and especially the woreda and zonal health officers, knew about the family folders, but indicated that their use was not being strictly enforced and monitored.

Current difficulties faced by HEWs in identifying recording vital events within their communities: The formative research revealed that HEWs face a number of challenges associated with monitoring vital events within their communities. These challenges included heavy workloads, the absence of a recording structure and necessary supplies and supervision, the lack of transportation especially given the large geographic size of most catchment areas, and delays in the distribution of the family folders. The suggestions offered to address these challenges included providing additional training, supplies, transport and per diems to HEWs for their work in recording vital events, and increasing awareness of the importance of recording vital events at population through newspapers and other media.

2.3.3. Formative research conclusions

The formative study concluded that reinforcing the existing Government system of having HEWs record vital events in family folders was the best option for RMM, but that the reasons why it had not been functioning should be addressed. Recommendations were to develop a clear system for reporting, to

provide adequate initial and refresher training and supervision, to ensure that continuous supplies of the materials necessary to do the work are available, and to strengthen incentives and means of transportation. Other recommendations from the formative research included mobilizing community member organizations and stakeholders to work with HEWs and community volunteers on recording vital events, introducing computer technology for the transfer and exchange of information, and stimulating stronger support for the HEWs' and CVs' work in this area from their supervisors and zonal leadership.

2.4 Ethical clearance

The project received ethical clearance from the Johns Hopkins Bloomberg School of Public Health's Institutional Review Board and from the Oromia Regional Health Bureau. Informed consent was obtained for respondents during primary data collection involving interviews with the HEWs or the population.

2.5 Implementation of the community-based RMM method

2.5.1 Selection and training of the Miz-Hasab RMM team

MHRC identified and recruited eight research assistants for the RMM project: 2 supervisors, 2 data collectors, 1 data managers, 1 data editor and 2 data entry clerks. This team was based at MHRC. The roles of the supervisors and data collectors were to conduct monthly field missions to collect data and provide additional data extraction tools and needed support to woreda RMM teams.

Most research assistants had been involved in various studies at the Center for an average of 10 years, and had 'good' to 'very good' abilities in understanding Amharic and Affan Oromo. A resident advisor was recruited by IIP-JHU and based in Addis to provide technical and managerial support to the project. The entire team was supervised and coordinated by the local Principal Investigator, the Director of MHRC.

Although most of the staff mentioned above were already familiar with the concepts and objectives of RMM, we conducted a one-week formal training to ensure clear understanding of the data collection procedures and quality assurance protocols. The content of the training addressed:

- The concept and objectives as well as the terms and terminologies of RMM;
- The purpose of the RMM instruments (e.g., family folders, filing boxes, extraction forms, cell-phones, backpacks, etc.);
- The procedures for collection and transfer of vital statistics data from households to health posts and to woreda health offices;
- Plans and materials for training WFPs, HEWs and CVs on their respective roles in RMM;
- Monthly collection of data from Woreda health offices;
- Plans for the three- and six-month assessments of the data; and
- Data entry and analysis.

In addition, the training included the usual issues of caution and discretion in dealing with 'incentives' and payments to public health workers. A hallmark of the health system in Ethiopia, the government of Ethiopia

strictly proscribes provision of large incentives to the HEWs to avoid detracting them from their assigned job. The training was provided by the local PI for the study and the project officer from IIP/JHU as well as the two supervisors.

2.5.2. Sampling

We determined the number of HEWs required to obtain an annual mortality rate that falls within ±20% of the rate calculated from the endline household survey with full birth history. We used a test of equivalence between the mortality rate generated from the HEW data (p1) and the rate produced by the endline household survey (p2) to estimate the sample size required if p1 is within 20% of p2, the acceptable margin of equivalence. More precisely, we assumed that the two methods are equivalent if p1-p2 falls within a set confidence interval. Assuming an under-five mortality rate of 0.092,[§] this implies that p1-p2 must fall within a 95% confidence interval of (-0.0184; 0.0184) with 80% power to reject the hypothesis of non-equivalence between the two methods. We used the Statistical Analysis System (SAS) to compute the corresponding sample size using simulations with 100,000 replications. The design effect reported for the under-five mortality rate in the past 10 years preceding the DHS 2005 for rural Ethiopia is 1.5. (see DHS 2005 report). We estimated from the DHS 2005 data that there was an average of 54 births per cluster in the rural area. Each kebele has about 5,000 population, corresponding to 1,000 households. With a rural crude birth rate of 40 per 1,000, it is expected that there will be 200 births annually in each cluster. Assuming the same intra-class correlation as for the kebele, a cluster design effect of 1.5 will correspond to a design effect of 2.9 at the kebele level.[**]

Table 4 indicates that a sample size of 80,000 households corresponding to 80 kebeles was required to validate one-year mortality. The large sample size of households was due to the larger design effect stemming from the clustering at kebele level. By adjusting the sample size to account for possible attrition among HEWs – set at 10% – the total number of kebeles needed be 89, corresponding to 100 HEWs for validation of one-year mortality. With 31 woredas in the evaluation zones, this implied that three kebeles needed to be randomly selected from each woreda for a total of 93 kebeles. For the endline household survey, a sample size of at least 26,300 households was required to validate a one-year mortality rate.

[§] This mortality rate is estimated by projecting the rural mortality rate estimated in the 2005 DHS from year 2000 to 2010 using an annual rate of change of 3.8%. The annual rate of change is obtained from mortality data reported by the UN Inter-Agency Group on Mortality Estimation (IGME). See full proposal for more details.

[**] Using the formula deff=r*(m-1)+1; with r=intra-class correlation and m the number of units by cluster

Table 4. Sample size for the RMM method based of HEW recording of births and deaths

Parameters	HEW Recording method
**Mortality rate**	0.092
**Relative difference to detect**	± 20% of MR
**Confidence level**	0.95
**Power**	0.8
**Cluster size (number of births)**	200
**Design effect**	2.9
**Crude birth rate**	0.04
**Average household size**	5
**Non-response rate**	0
**Number of births required**	16000
**Validation of one-year mortality rate**	
Number of households	93,000
Number of kebeles	80
Adjustment for 10% attrition	89
Adjustment for 3 kebeles per woreda	93
Total number of HEWs	178

Random selection of RMM kebeles. We worked with zonal and woreda health offices to obtain the complete updated list of all kebeles and population sizes. We then randomly selected three kebele within each of the 31 woreda with probability proportionate to the population size of the kebele. Selected kebeles were then assessed for accessibility and those found to be very remote and inaccessible either by vehicle or foot were replaced by selecting the next random kebele.

Figure 2. Map of Jimma and West Hararghe showing selected RMM kebeles

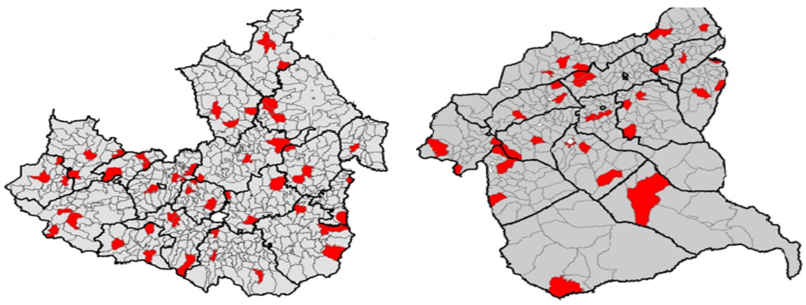

Note: Seven newly created kebeles could not be located on the West Hararghe map

2.5.3. Implementation

The implementation of the RMM method based on HEWs' recording of births and deaths within their communities was guided by findings from the formative research. We tried to address at the outset some of the issues raised during the formative study, while avoiding any serious disruption to the existing system.

The implementation of the RMM method therefore consisted of the following set of activities, listed in sequential order:

- Informational meeting with regional, zonal and woreda health offices;
- Identification of HEWs in the selected RMM kebeles;
- Finalization of data collection instruments and their translation into local languages;
- Recruitment and training of research assistants to serve for field data collection and data entry;
- Training of HEWs, community volunteers and woreda focal point persons;
- Provision of data collection tools, equipment and incentives;
- Deployment and start-up of recording and reporting;
- Ongoing vital events data collection; and
- Three- and six-month assessments of the data for consistency, accuracy and completeness.

The formative research showed that government family folders were not yet implemented in West Hararghe zone and therefore were not known by the HEWs in that zone. The RMM team decided to use temporary folders, referred to as "kebele folders" (KF). These folders were designed and printed by MHRC and provided to RMM HEWs in West Hararghe zone. HEWs were instructed to record information on families in which an event was identified on a short form that was filed in the kebele folders. Similar to HEWs in the Jimma zone, they extracted vital events information from these folders onto supplied extraction forms and send them to the woreda focal point person.

Further details on these activities are presented in the sections that follow.

Sensitization and engagement of the Oromia Regional Health Bureau (ORHB) and Woreda Health Bureaus (WHBs). Two MHRC research teams (each with two researchers and a vehicle) travelled to Jimma and West Hararghe zones on Sunday (Oct 23, 2011) primarily to discuss the RMM project with zonal and woreda officials, to assess the status of family folders (FFs) and assess the feasibility of establishing a kebele folder in health posts where family folders were not yet implemented. The teams returned to Addis on Friday November 4, 2011.

This informational and assessment visit suggested that it would be possible to use existing FFs in Jimma zone, but that it would be necessary to develop and implement KFs in West Hararghe zone.

The zonal and woreda health officers welcomed the project and agreed to its relevance and implementation in the selected zones and kebeles. The MHRC research teams, with the help of the woreda health office, proceeded to identify the appropriate person within the WHB that could serve as focal point person for the project.

Selection of HEWs. Following the random selection of the kebeles for participation in RMM (see section II.2.3 above), HEWs serving in the selected kebeles were approached and invited to participate in the project.

Data collection instruments. The MHRC, in collaboration with IIP-JHU developed piloted and finalized the kebele folders and the data extraction forms. As per the findings of the initial visits to the zonal and woreda towns, these forms were translated into Amharic and Affan Oromo, the local language of the study areas. Annex 4 provides samples of the data extraction forms in both English and Affan Oromo languages.

Training of HEWS, woreda focal points (WFPs) and community volunteers (CVs). Training manuals and posters were developed by MHRC for the training of HEWs, WFPs and CVs. The WFPs appointed in each woreda were contacted to invite HEWs under their supervision and selected community volunteers to the training. Four teams of trainers traveled to each of the 31 woreda to conduct a one-day training of the HEWs, WFPs and CVs, which took place December 11-27, 2011. The in-country PI and JHU/IIP project officer travelled to few training locations to ensure consistency and accuracy of training being provided by trainers. Large canvas posters showing the data collection and extraction forms were posted on the walls of the training rooms for use in explaining the types of data to be collected and the correct data formats. The one-day training program included a description of the RMM and instructions on how to complete the RMM forms for recording vital events. Trainees participated in exercises to practice filling out the forms in hypothetical situations.

The trainees were invited from the two zones in Oromia region. A total of 31 WFPs, 186 HEWs and 93 CVs were invited to come to woreda towns for the training, including one WFP from each woreda town, 3 HEWs and 1 CV from each of the 93 selected kebeles.

Table 5 provides a summary of training attendance. All WFPs who were invited attended. Only 87.1% of the HEWs and 75.3% of the CVs who were invited attended, with better attendance in Jimma zone than in West Hararghe zone. Further details on the trainees are available in Annex 1

Table 5. Summary of attendance of trainees at the training

Zone	Trainee's Position	Percent attended
Jimma zone	WFP	100% (17 out of 17)
	HEW	94.2% (96 out of 102)
	CV	88.2% (45 out of 51)
West Hararghe	WFP	100% (14 out of 14)
	HEW	78.6% (66 out of 84)
	CV	59.5% (25 out of 42)
Total	WFP	100% (31 out of 31)
	HEW	87.1% (162 out of 186)
	CV	75.3% (70 out of 93)

Incentive structures to the RMM. Limited incentives were provided to the RMM HEWs and the WFPs for their participation in RMM. At the end of the initial training, all HEWs were provided with backpacks and stationary, including extractions tools and pens. They also received transportation allowances for every month that they submitted a report to the WFP after a trip from their health post to the woreda town to meet with the WFP. Kebeles in West Hararghe, (where family folders were not yet implemented), were provided with a kebele folder in which to record vital events. The WFPs were provided backpacks, cellphones, and airtime at the outset of the RMM implementation period. The cellphones were provided to facilitate communication with the MHRC team. A pool of community volunteers were also identified in each kebele and provided with a small bag as an incentive.

Data collection and supervision. The HEWs were deployed in December 2011, and asked to start reporting data immediately. Data reported for the month of December were not included in the assessment.

The implementation period for the community based method in Ethiopia was from January 2012 through April 2013.

The data collection process consisted of HEWs recording vital events in their family folders or kebele folders as they were identified throughout the month. At the end of the month, the HEWs extracted the pregnancies, births and deaths they had recorded onto the extraction forms. These forms were picked up by or sent tothe WFPs every month. The WFPs were in constant communication with the HEWs, and together they would decide on the best way to transfer the forms – either by having the HEWs take them to the woreda town during their routine trips, or by having the WFP travel to the health post. The WFPs first reviewed the data and then handed them over to the MHRC research assistants during their monthly visit to the woreda. Four teams of two MHRC research assistants visited each woreda monthly to collect the extraction forms, discuss data quality issues with the WFPs, and visit selected health posts. All unresolved data issues were followed up through phone communications between the research assistants and the HEWs. All data forms collected were delivered to the MHRC offices in Addis where they were compiled, edited and entered into a CSPro database developed for that purpose.

Figure 3. RMM structure including data flow and supervision

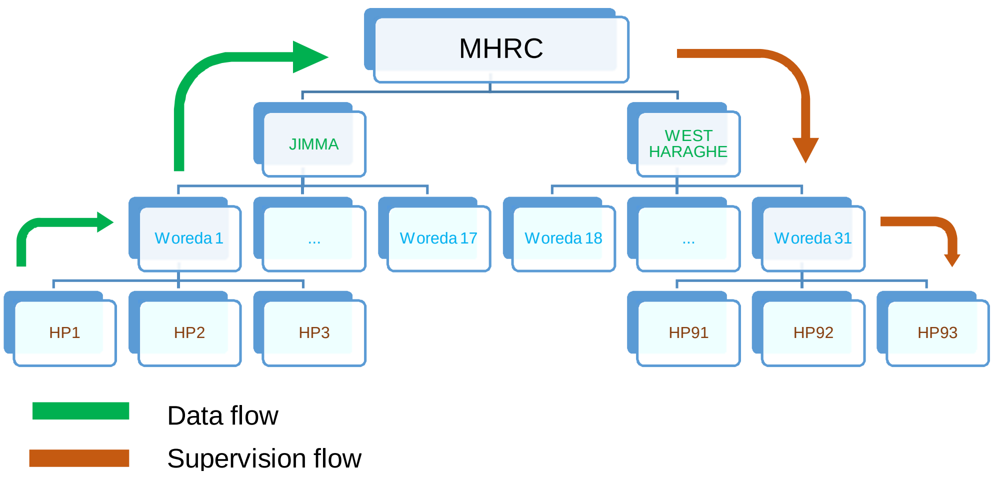

Challenges. In this section we describe the challenges encountered in delivering the community-based RMM method in Ethiopia, and the steps we took to address them.

Untrained HEWs and CVs: As indicated above, there were a few HEWs and CVs, especially in West Hararghe zone, who missed the training for various reasons. This was a problem during data collection. In order to correct this, our short-term solution was to ask the WFPs or trained HEWs to train their colleagues who missed the training. In addition, because MHRC researchers were scheduled to visit the woredas in early January 2012, we arranged for them to repeat the training at that time and invited all HEWs and CVs who missed the original training to attend.

First month pregnancy data collection: In all the woredas, RMM data collection in December 2011 required the identification and listing of all pregnant women. This required tremendous amount of work.

Different calendars: The Ethiopian calendar (the working calendar in the country) is different from the Gregorian calendar. For instance, the equivalent of December 15, 2011 (12/15/2011) in the Ethiopian calendar would be Tahisas 5, 2004 (04/05/2004). In order to ensure that we collected data for a full 12 months, we converted the Ethiopian calendar to its Gregorian calendar using a software application titled The Amharic software 'Power Geez' during data entry in CSPro.

Absence of family folders in West Hararghe zone: The absence of family folders in the entire zone posed a challenge to our tasks. First, without the family folders, HEWs/CVs were not familiar with recording vital events. This meant our training and follow-up efforts had to be intensified. Second, we had to supply them with temporary folders used only for the purposes of the RMM project, and devised a numbering system to produce unique identifiers. We were concerned that this might conflict with the later introduction of the Government family folders and we have worked closely with zonal and regional authorities to reduce any potential impact this may have had.

Woreda level meetings, trainings, campaigns: One of the challenges was how to handle the regular, and sometimes unscheduled, meetings and trainings of WFPs and HEWs at the woreda level. We witnessed the potential problems this might raise early on, when we were told that there would be a training of all HEWs in the two zones (in fact in all of Oromia) during the last week of December 2011 and the first week of January 2012. Such events, including health campaigns, happened regularly, posing a problem to the continuous recording vital events. We tried to address this by maintaining frequent communication with the HEWs and WFPs to anticipate when such an event would happen and to work with them to develop strategies for capturing any events that were missed.

Other challenges: Additional challenges that needed to be addressed in the course of implementing this RMM approach are summarized briefly below.

- In some kebeles we were concerned about the capacity of the HEWs to complete the data collection and extraction forms accurately and consistently. We tried to address this by being in touch with the HEWs frequently by cell phones to identify and address any issues or questions that arose during the completion of the forms. The MHRC team also provided feedback and worked to solve problems during their field visits.

- In our opinion, the incentives offered to WFPs and HEWs for their participation in RMM were an important motivation. We made special efforts to ensure that these incentives were distributed fairly and maintained constantly to ensure continuous support for project activities.

- Some woreda health officials expressed concern that RMM tasks might detract from the other responsibilities of participating HEWs. During field visits we offered frequent reminders that tracking vital events is a part of the standard job description for these workers.

- We observed that when distance between kebeles and woreda towns was an issue, WFPs were reluctant to travel long distances to collect the monthly data from HEWs. It was in response to this issue that the option of having HEWs bring the data to the WFPs on a monthly basis was developed.

Opportunities. In spite of the challenges and concerns mentioned above, there were a number of opportunities that we exploited in order to make RMM successful. We highlight several examples below.

- Adequate and dedicated MHRC staff: MHRC made the decision to have a dedicated team of staff responsible full time for RMM activities. The initial group of four full-time staff members was increased to eight over the course of the project. These eight full-time staff were responsible for the training of WFPs, HEWs and CVs; conducting regular follow-up to make sure that HEWs and CVs were recording vital events accurately and consistently; travelling to the woreda towns (and sometimes to the kebeles) to monitor data collection and to re-supply collection forms. MHRC had also put in place a permanent data entry team of three persons who were responsible for editing, coding and double entering all the data on a monthly basis. We believe that having full-time staff rather than a larger number of part-time staff who divided their time between RMM and other responsibilities was a good strategy and led to better performance.

- Support from IIP/JHU: MRHC reports that the technical assistance provided by IIP/JHU was essential for the successful completion of the project, and point particularly to IIP-JHU help in developing and finalizing the data collection and extraction forms, the training of WFPs, HEWs and CVs, and planning the follow-up of data collection activities.

- High levels of commitment from WFPs, and HEWs: We had noted from our training experience that the WFPs showed dedication to help on recording vital events by HEWs and CVs. They had all attended the training and shown interest to fully participate, barring some unforeseen circumstances. Similarly, HEWs who attended the trainings, particularly from Jimma zone, had shown the capacity and willingness to carry out their tasks.

We believe that the ability of the local RMM team to adjust to circumstances in the field was crucial to the successful implementation of RMM. This suggests that future, similar approaches should be sufficiently decentralized to allow for local problem solving and response.

Timeline of implementation and data collection. Figure 4 below presents the timeline for RMM implementation and analysis. In addition to the monthly data collection, two interim assessments were conducted. The first at three months of implementation assessed data consistency and reporting barriers. The second assessment at six months of implementation assessed accuracy and completeness in data reporting.

Figure 4. Timeline of RMM implementation in Jimma and West Hararghe

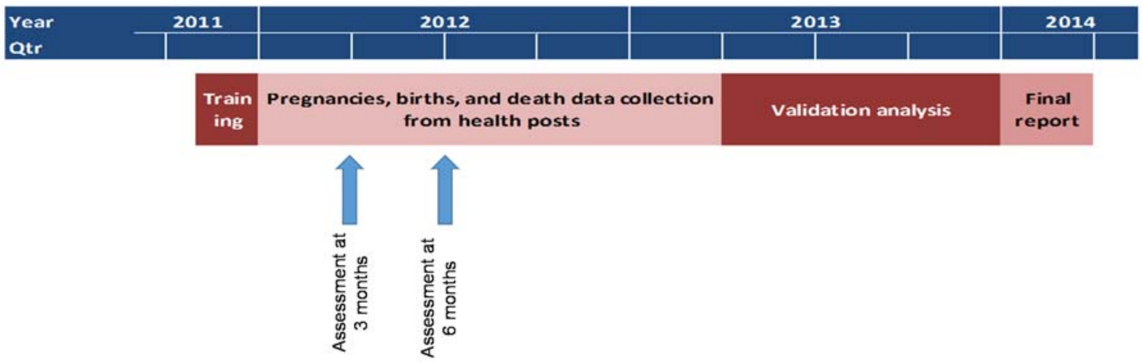

Data entry and processing. A CSPro database was developed for entry of all pregnancy, birth and death extraction forms. Data forms were first reviewed by the IIP-JHU RMM program officer, then edited and

double-entered into the database by two separate data entry clerks. The two entries were then reconciled before being exported into SPSS for analysis. MRHC sent monthly progress reports IIP-JHU. The MHRC RMM team conducted the basic analysis.

2.6 Interim data quality assessment I (DQA I)

2.6.1. Background and objectives

Between mid-December 2011 and early May 2012, 1053 births and 239 deaths were recorded by HEWs in the 93 RMM kebeles through the community-based RMM method (Table 6). The proportion of kebeles reporting data was high in Jimma zones between December and March, and moderate reporting levels improved in West Hararghe over the 4-month period (Table 7).

After three months of RMM implementation, we conducted an assessment of data consistency and reporting barriers. The objectives of the three-month assessment were to document the quality of implementation of data collection procedures and the quality of data collected in terms of its internal consistency and external consistency with health post records.

Table 6. Summary of RMM data, December 2011 to early-May 2012

Zone	Pregnancies	Births	Deaths
Jimma	1,796	530	165
West Hararghe	1,645	523	74
Total	3,441	1,053	239

Table 7. Proportion of kebeles reporting RMM by month

Zone	Dec/Jan	Feb/Mar
Jimma	49 (out of 51) (96%)	50 (out of 51) (98%)
West Hararghe	27 (out of 42) (64.3%)	39 (out of 42) (92.8%)

2.6.2. Methods

The three-month assessment was conducted between April 20th and May 8th 2012 in one randomly selected RMM kebele in each woreda (31 total). All selected kebeles were contacted directly or via WFPs to schedule visits to each kebele health post to conduct the assessment. HEWs were absent in two selected kebeles, so only 29 kebeles were included in the full assessment. A total of 55 HEWs, 31 WFPs, and 23 CVs were included in the assessment. Data extraction forms were developed to verify previously reported vital events against those in kebele folders and check the labeling of kebele folders. HEWs were interviewed about challenges they had encountered collecting and reporting data, and about the involvement of CVs. WFPs were interviewed about supervision of HEWs and perceived barriers to data reporting. CVs were interviewed to assess involvement in data collection. Data were entered and analyzed in SPSS.

2.6.3. Findings

Characteristics of HEWs. In an average kebele, 2 HEWs and 20 CVs (range: 2 – 58) served a population of approximately 5000 individuals (range: 1550 – 10169). HEWs had typically served in their role for less than 2 years (range: 4 months – 6 years) and 25% of HEWs did not live in the kebele where they worked. A majority (72%) of HEWs reported meeting with their CVs at least once every 2 weeks, although these whether meetings were specific to RMM was not ascertained. In West Hararghe (relative to Jimma), more kebeles had only one acting HEW, HEWs on average had less experience in their roles, and HEWs reportedly meet with CVs less frequently.

Implementation and utilization of kebele and family folders. In West Hararghe, kebele folders were created explicitly for use in RMM data collection. In Jimma zone, folders existed prior to RMM and were used for other activities. Kebele and family folders were used in all selected kebeles. All folders in West Hararghe, and a majority of randomly sampled folders in Jimma, were correctly labeled with household identification numbers. In Jimma, some family folders were not found within their folders, suggesting that HEWs may be inconsistently returning folders when they are used for non-RMM activities.

Consistency of data. Vital events reported to us through the community-based RMM method were compared to the births and deaths recorded in family folders at health posts during the first three months of data collection. Among the 24 kebeles reporting births, 92% of births reported through RMM had been recorded in their respective family folders, with a higher proportion of births recorded in West Hararghe (99%) compared to Jimma (81%) zones. Among the 14 kebeles reporting at least on death, 91% of deaths reported through RMM had been recorded in their respective family folders, with a higher proportion of births recorded in West Hararghe (100%) compared to Jimma (88%) zones. High proportions of information on date of birth (96%) and age of death (94%) was consistent between the reported RMM and health post data.

Barriers to data reporting. WFPs were interviewed about supervision of HEWs and perceived barriers to correct and consistent data reporting. All WFPs reported travelling to all three of their kebeles to supervise HEWs at least once in the previous three months, and 68% reported supervising HEWs monthly. Only 16% reported travelling to kebeles to collect RMM data as instructed through the original reporting strategy. This finding was consistent with our previous reports of inconsistent data collection, which necessitated a change in the strategy of RMM data collection from being collected by WFPs to be delivered by HEWs on a monthly basis. Under the new strategy, WFPs reported that HEWs and occasionally other individuals brought data to WFPs. Issues with HEWs' poor understanding of family folder numbering were commonly cited in both zones. Additional training, "encouragement" of HEWs through monetary incentives, and more frequent meetings between WFPs and HEWs were the main suggestions by WFPs for improving HEW recording and reporting.

Community volunteer assistance in reporting vital events. Among 21 CVs interviewed, 20 reported meeting with an HEW at least once and 64% reported meeting at least 3 times in the past 3 months to "discuss health issues." All interviewed CVs reported informing HEWs of pregnancies and births within their community. Only a quarter reported informing HEWs of deaths, though this may be because there were no deaths in their community during this period.

2.6.4. Conclusions

We found high overall consistency in vital events reported to us through the community-based RMM method compared to vital events recorded in health post family folders, although these findings do not speak to the completeness of event reporting beyond those identified in RMM data. The somewhat poorer consistency observed in Jimma zones may be related to the use of family folders for non-RMM activities and their occasional misplacement in these zones.

Lack of consistent RMM task completion by WFPs suggests precaution is needed when interpreting WFP opinions on HEW barriers to recording and reporting data. Issues with poor understanding of family folder numbering were cited by WFPs in both zones, although our team did not see this as a major problem especially in areas were folders were created as a vital event were observed. The WFP suggestion of additional training of HEWs was taken into account in consideration of a 6-month refresher training of HEWs. The hesitation of WFPs to travel to kebeles to collect data from HEWs was reflected in the low proportion of WFPs who reported doing so, supporting the development of a new option for the reporting of RMM data whereby the HEW brings the data to the WFP monthly. Distance, lack of transportation, and frequent meetings and campaigns are barriers to HEW data delivery and meetings with WFPs. Additional incentives to encourage and support HEWs to bring data to WFPs could be considered in light of reduced costs to the central research team.

Although the data suggest that CVs are numerous and that they do engage regularly with the HEW, we learned little about the motivation of these volunteers or the quality and reliability of the information they generate.

2.7 Interim data quality assessment II (DQA II)

2.7.1. Background and objectives

To learn the degree of completeness of vital events reported by HEWs, we planned an accuracy and completeness assessment at six months of data collection. However, due to delays in obtaining ethical approval, actual data collection for the assessment occurred in September 2012, nine months in data collection.

At nine months of implementation, we conducted an assessment at kebele and household levels to estimate the accuracy and completeness of data reported by HEWs. The objectives of the six-month assessment were to determine: (1) the accuracy of reported vital events through household verification; and (2) the level of completeness/underreporting of data reported by HEWs.

2.7.2. Methods

The assessment was conducted in one randomly selected RMM kebele in each woreda, for a total of 28 kebeles, after exclusion of inaccessible kebeles. Within each selected kebele, all births and deaths that occurred up to May 30, 2012 were listed from the RMM database. Within each kebele, a sample of seven births (194 total) and all under-five deaths (34 total) were selected and verified through home visits. Data consistency was further assessed by checking if all births and under-five deaths (and key identifying variables) reported through RMM in the selected kebeles were also recorded in health post level family folders. Data completeness was assessed through key-informant interviews (KIIs) to identify births and under-five deaths that may have occurred between January and September 2012 and cross-checked

whether these events had been reported to us through the community-based RMM method. Within each selected kebele, we identified up to six key informants with the assistance of kebele officials and HEWs (223 total). Key informants generally included kebele managers, traditional birth attendants, teachers, religious leaders, and village elders. Each key informant was solicited to provide information on births and child deaths that may have occurred in the kebele over the period of the assessment. If the initial 6 key informants did not have much information, up to an additional five key informants were identified and interviewed. A key informant interview guide was used to solicit information on births and under-five deaths. Data were entered and analyzed in SPSS.

2.7.3. Findings

Data consistency and accuracy. The six-month data consistency assessment showed that, in both zones a large majority of reported birth (97%) and deaths (88%) reported through RMM were also found in the family/kebele folders at the health posts (Table 8). The majority of births (94%) and deaths (91%) were also confirmed through household visits, and those unconfirmed events were isolated to households that had moved away from the kebele.

Table 8. Birth and under-five death data consistency and accuracy, January – September 2012

Zone	RMM births verified by households	RMM births matched to health post data	RMM deaths verified by households	RMM deaths matched to health post data
Jimma	102/110 (92.7%)	105/110 (95.4%)	13/14 (92.8%)	12/14 (85.7%)
West Hararghe	81/84 (96.4%)	83/84 (98.8%)	18/20 (90.0%)	18/20 (90.0%)
Total	183/194 (94.3%)	188/194 (96.9%)	31/34 (91.2%)	30/34 (88.2%)

Data completeness. Data completeness was assessed through identification of events through KIIs. In total, 895 births and 122 under-five deaths were identified by key informants. A random sample of births and all under-five deaths were verified with households and compared to health post family folder data. All sampled KII identified events were verified by household members (Table 9). Birth data completeness was low, as only 46% of randomly sampled and verified births had been recorded at health posts. Underreporting of under-five deaths was greater than underreporting of births. Only 22% of under-five deaths identified through KIIs had been recorded at health posts. Data completeness was slightly higher in Jimma zones, compared to West Hararghe, for both births and under-five deaths. Many households identified by key informants as having an event (and confirmed at the household level) did not have kebele/family folders at the health posts or events were not filed in folders.

Table 9. Birth and under-five death data completeness, January – September 2012

Zone	KII births verified by households	KII births matched to health post data	KII deaths verified by households	KII deaths matched to health post data
Jimma	160/160 (100%)	75/160 (46.9%)	56/56 (100%)	14/56 (25%)
West Hararghe	120/120 (100%)	53/120 (44.1%)	49/49 (100%)	9/49 (18.4%)
Total	280/280 (100%)	128/280(45.7%)	105/105 (100%)	23/105 (21.9%)

2.7.4. Conclusions

Although data accuracy and consistency were high, data completeness was poor. Almost all events that were identified by the key-informants were confirmed at the household level, indicating a high quality of the data collected from informants. There appears to have been substantial under-reporting in both births and under-five deaths by the HEW. Observations and feedback from HEWs and kebele leaders suggest the main reasons for this under-reporting may include:

- Many kebeles only had one HEW;
- HEWs were overburdened with multiple tasks;
- Distance and transportation issues made it difficult to reach remote areas or cover large kebeles to register events;
- HEWs were sometimes absent for extended periods due to pregnancy/childbirth, sickness, or stays in woreda towns;
- HEWs were often members of kebele administration or spouses of local politicians, making them difficult to supervise and vulnerable to underreporting events for political reasons;
- Coordination between HEWs and CVs was poor;
- Supervision by WFPs was low; and
- Cultural taboos around infant mortality discouraged public disclosure of deaths.

This feedback suggests completeness of data reporting could be improved with provision of transportation support, a reduction in the scope of HEW responsibilities, an increase in the number of HEWs, or recruitment of HEWs who reside in their catchment areas.

2.8. Endline validation survey

2.8.1. Background and objectives

The endline validation survey was used to assess the accuracy and reliability of data collected through the community-based method relative to full pregnancy histories collected using current best practice household survey methods. The survey was conducted primarily as the endline survey for the larger evaluation of the ICCM strategy in Oromia. The RMM objectives of the endline validation survey were to:

- Assess the level of completeness of births and deaths reported to MHRC by HEWs;
- Determine the patterns of births and deaths that are likely to have been missed by HEWs; and
- Compare neonatal and under-five mortality rates from data reported by HEWs and the endline survey

2.8.2. Sampling

Sample size. As previously stated in section 2.5.2 and presented in Table 4, we calculated that a sample size at least 26,300 households would be required to validate a one-year mortality rate. However for the larger evaluation, a sample size of 28,000 households was required. Thus the endline survey included a sample of 28,000 households, of which 27,872 were successfully reached during the survey. In total, 26,791 women aged 15-49 participated in the women's interview.

Sampling frame and sample selection. The endline household survey used a stratified two-stage cluster sampling design with stratification by woreda. The 2007 population census's frame of enumeration area (EAs) was updated in terms of the distribution of the EAs by woreda and used for the primary unit sampling. Thus, at the first stage, EAs were randomly sampled by the Ethiopia Central Statistical Authority (CSA), using systematic random sampling with probability proportionate to size of the enumeration areas.

A map of each selected EA was printed and provided to data collectors. A complete listing of households in each EA was conducted prior to data collection to establish the sampling frame of households from which 34-36 households were selected. EAs were selected for segmentation if they were above the 75th percentile in either population or geographic size (greater than approximately 200 households or 10 km^2) for each of the two zones. The EAs identified for segmentation were divided into either two or three segments and a segment was randomly selected. The segmentation was mainly done in office prior to deployment of enumerators. However, when the enumerators encountered very large EAs in the field, they also proceeded to segmentation in the field using similar procedures. Households in EAs or segments retained for interviews were listed completely to generate a sampling frame of households. Each team supervisor then conducted a random sampling of 34-36 households using systematic random sampling with equal probabilities.

The heads of households in the randomly selected households were interviewed and asked for a listing of all household residents along with the age, gender and relationship to the head of household. All women of childbearing age (15-49 years) residing in the selected households were identified and asked information on their background characteristics and their full birth history. Within each household, the head of household (or a comparable adult) was interviewed using the Household Questionnaire and women aged 15-49 were interviewed using the Women's Questionnaire.
Three EAs selected for interview could not be surveyed due to on-going conflict in two of them. For the third, the population had completely relocated.

2.8.3. Survey response rate

This survey was conducted from February 26, 2013 to May 31, 2013 in both zones. The survey report includes details of the survey implementation and is available on request. Data quality assessment of the mortality module of the survey is presented in Annex 2. We present in Table 10 below the response rate of the survey. The response rate exceeded the minimum requirement of 26,300 participants for the household and women's interviews

Table 10. Number sampled and interviewed households and women 15-49

Sample	Study Arm		Zone		Total
	Intervention	Comparison	Jimma	West Hararghe	
Sampled number of households	14,003	13,999	15,330	12,671	28,002
Number of households interviewed	13,996	13,876	15,316	12,556	27,872
Household interview response rate (%)	99.9	99.1	99.9	99.1	99.5
Number of eligible women (15-49)	13,460	13,455	14,994	11,921	26,915
Number of eligible women interviewed	13,424	13,367	14,946	11,845	26,791
Women 15-49 response rate (%)	99.7	99.3	99.7	99.4	99.5

2.9. Validation Analysis

Data on births and under-five deaths reported by HEWs in selected West Hararghe and Jimma kebeles for the period of January 2012 to March 2013 were included in the validation analysis. We calculated the number of births and neonatal, infant, and under-five deaths for 2 rolling periods of twelve months. The first period covered January 2012 to December 2012 and the second covered April 2012 to March 2013. Our validation analysis compares reported birth counts, death counts and associated mortality rates with those estimated from a full birth history collected in the endline survey.

2.9.1. Community-based RMM Method

The data collected through RMM were maintained and analyzed using Stata 13. Descriptive statistics were generated on a regular basis following monthly data cleaning procedures described in the previous methods section. These descriptive statistics were tracked on an Excel spreadsheet maintained and updated by the RMM resident advisor at MHRC. Descriptive statistics were used to track the completeness of HEW monthly reporting and numbers of births and deaths (specifically under-five, infant, and neonatal) reported by HEWs by month. The data were also used to assess overall quality of the collected data through the generation of sex ratios, ratios of reported infant to under-five deaths and neonatal to infant deaths, and various distributions of death by time. Figures were created in Excel 2013.

2.9.2. Endline validation survey

The overall household and eligible women response rates were estimated in West Hararghe and Jimma, respectively. The *de facto* household population by five-year age groups, according to sex and zone was also presented. A preliminary internal quality assessment of the data on live births based on complete counts of live births was conducted by examining date of birth, and sex ratio at birth, age at death, and the age patterns of deaths, i.e.: ratio of early neonatal to neonatal deaths and neonatal deaths to infant deaths.

The neonatal (NNMR), post-neonatal (PNNMR), infant (IMR), child (CMR) and under-five (U5MR) mortality rates were calculated by year from January 2012 to March 2013 for West Hararghe and Jimma. All data analyses were conducted in Stata 13.0.

2.9.3. Validation analysis

We calculated neonatal, infant and under-five mortality rates by dividing the number of these deaths documented by HEWs in a given 12-month period by the total births documented by HEWs in that particular period.

Our validation analysis involved two components: (i) an evaluation of the completeness of births and deaths reporting by HEWs, and (ii) a comparison of under-five, infant and neonatal mortality rates constructed from the HEW data with those estimated from the endline survey for the respective 12-month periods.

To evaluate the completeness of births and under-five deaths documented by the HEWs, we estimated the expected number of births and under-five deaths for periods of 12-month. We estimated the crude birth rate and the under-five mortality rate directly from the endline validation survey. To estimate the expected number of births, we multiplied the crude birth rate for each annual period by the total population size of the two study zones. The expected number of under-five deaths was calculated similarly, by multiplying the under-five mortality rate estimated from the validation survey by the expected number of births. We examined completeness of HEW-reported births and under-five deaths reporting by analyzing the ratio of total number of births and under-five deaths documented by HEWs to the expected number estimated from the endline validation survey.

When comparing HEW-based mortality rates to those derived from the endline validation survey, we calculated the mortality rates in the same way to ensure direct comparability. Standard errors and confidence intervals were estimated using the jackknife resampling technique.

We used the Delta method to examine the equivalence of the mortality rates based on the HEW data and those based on the endline validation survey. We used a tolerance range of 20% of the survey mortality rate. We rejected the hypothesis of equivalence between the two mortality rates if the upper bound of the 95% confidence interval of ratio was less than 0.80 – concluding that the HEW-based data significantly under-estimated the under-five mortality rate. Similarly, if the lower bound of the 95% confidence interval of the ratio was greater than 1.20, we concluded that the HEW-based data significantly over-estimated the under-five mortality rate.

2.10. Results

2.10.1. RMM community-based method

The endline validation covers community-based data collected between January 2012 and March 2013. We report on the numbers of births and childhood deaths and the associated under-five, infant, and neonatal mortality rates. Whenever possible, we present the results in graphs; corresponding Tables are available in Annex 2.

Reporting of data on births and deaths by HEWs. The level of timely reporting of monthly extraction data was over 80% in both zones, except for February and March 2013 in West Hararghe. The timely reporting in West Hararghe was more erratic than in Jimma, a result of difficult access to health posts due

to rough topography and long distances in that zone. Figure 5 shows health posts that have reported timely data by month. HEWs that did not report data in a particular month generally catch up in the following months.

Figure 5. Percentage of health posts reporting data on timely basis by zone, (51 health posts in Jimma and 42 in West Hararghe), January 2012- March 2013

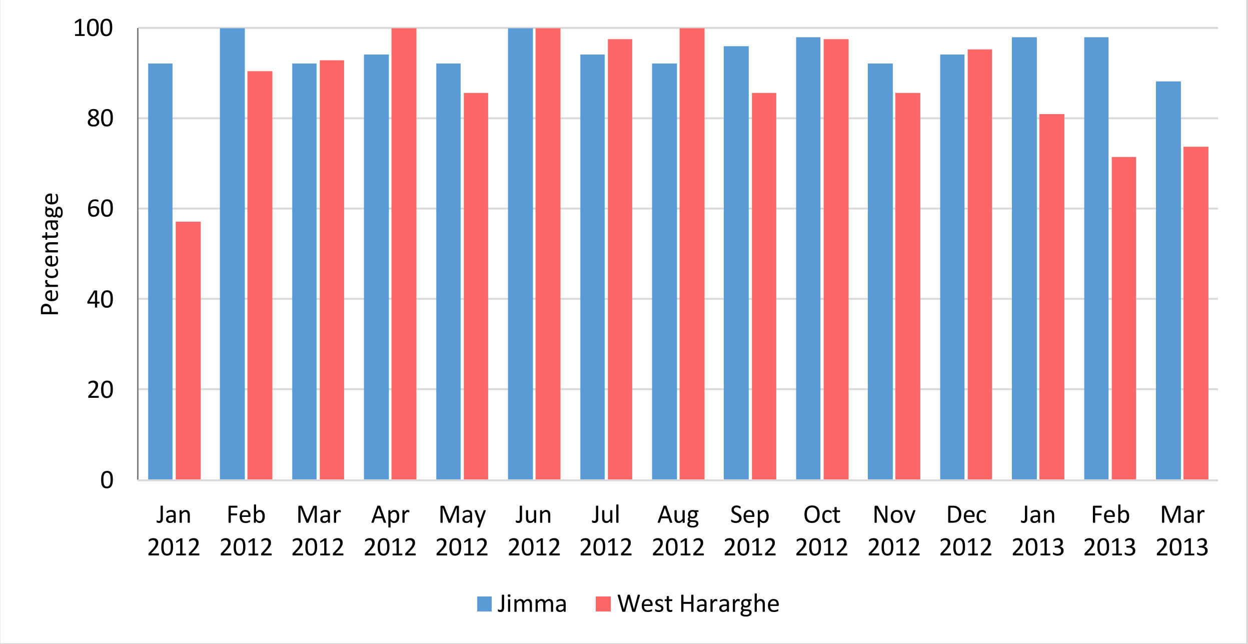

Reporting of data on births by HSAs. In total, the HEWs reported 5,860 live births in the 15-month validation period, 2,844 in Jimma and 3,016 in West Hararghe. Figure 6 presents data on the number of births reported by HEWs on a monthly basis between January 2012 and March 2013 in each of the two zones. In both Jimma and West Hararghe, the number of births generally increased for the first five months of the validation period. In Jimma, births then steadily declined for the next five months, whereas in West Hararghe births peaked in the months of May-July, 2012 before declining steadily for the remainder of the calendar year. We observe notable variation in both zones in documented births for the first three months of 2013.

Figure 6. Number of births reported by HEWs by month in Jimma and West Hararghe, January 2012- March 2013

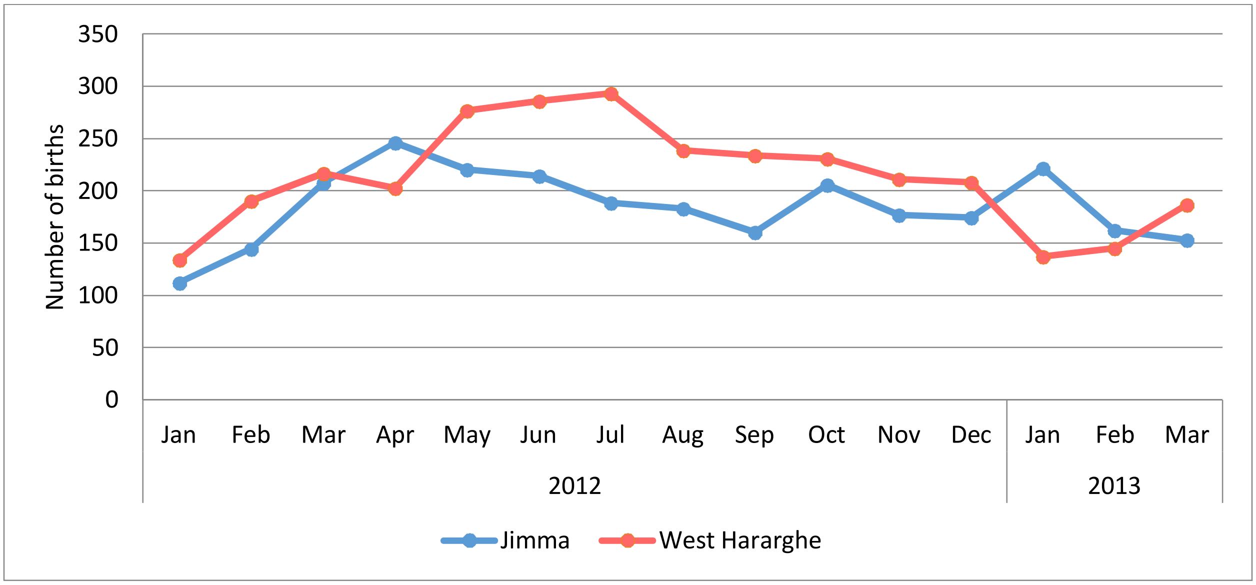

Reporting of data on deaths by HSAs: Under-five deaths. Figure 7 presents the number of under-five deaths reported by HEWs in the two zones by month between January 2012 and March 2013. The HEWs reported a total of 285 under-five deaths in the 15 month validation period, 143 in Jimma and 142 in West Hararghe. In West Hararghe the number of reported under-five deaths peaked during the rainy season around May-July, whereas in Jimma reported under-five deaths peaked earlier in April-May. The temporal trend in under-five deaths between August 2012 through to March 2013 is very similar for both zones with about 6-10 deaths being reported each month in each zone.

Figure 7. Under-five deaths reported by HSAs in Jimma and West Hararghe, 2012-2013

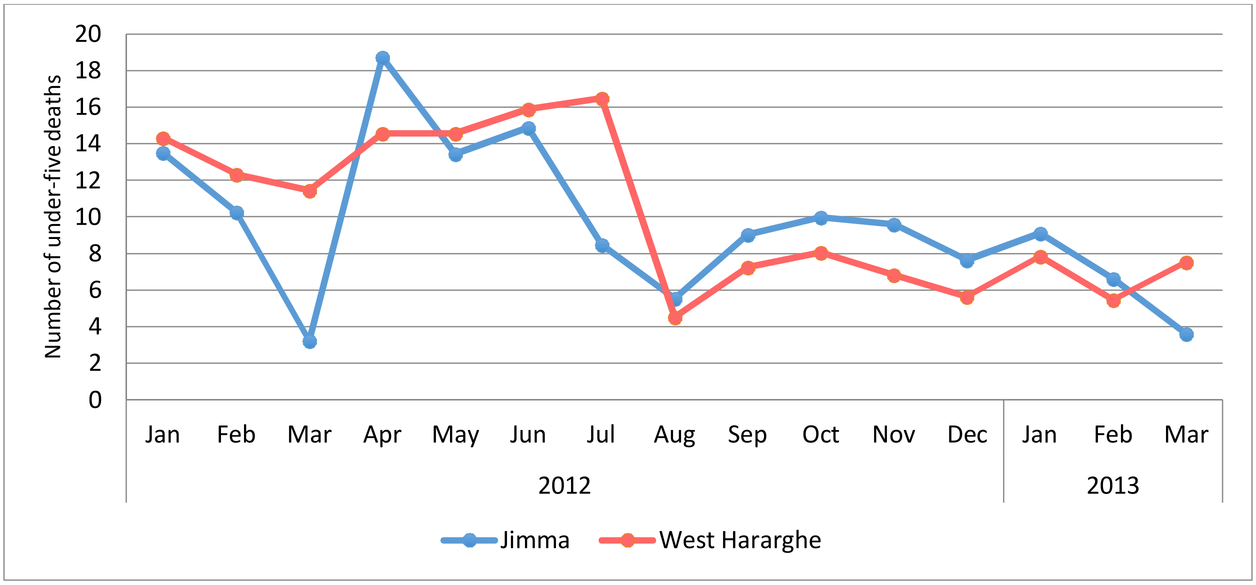

Reporting of data on deaths by HEWs: Infant Deaths. HEWs reported 229 infant deaths (i.e. deaths occurring before the child's first birthday) during the validation period, 114 in Jimma zone and 115 in West Hararghe. Figure 8 presents the number of infant deaths reported by HEWs in the two zones, by month between January 2012 and March 2013. The number of infant deaths is small in both zones; too small to draw any general conclusions about patterns. However, the patterns generally appear to mirror those reported for reported under-five deaths given that 78% of reported under five deaths were infant deaths.

Figure 8. Infant deaths reported by HEWs in Jimma and West Hararghe, January 2012- March 2013

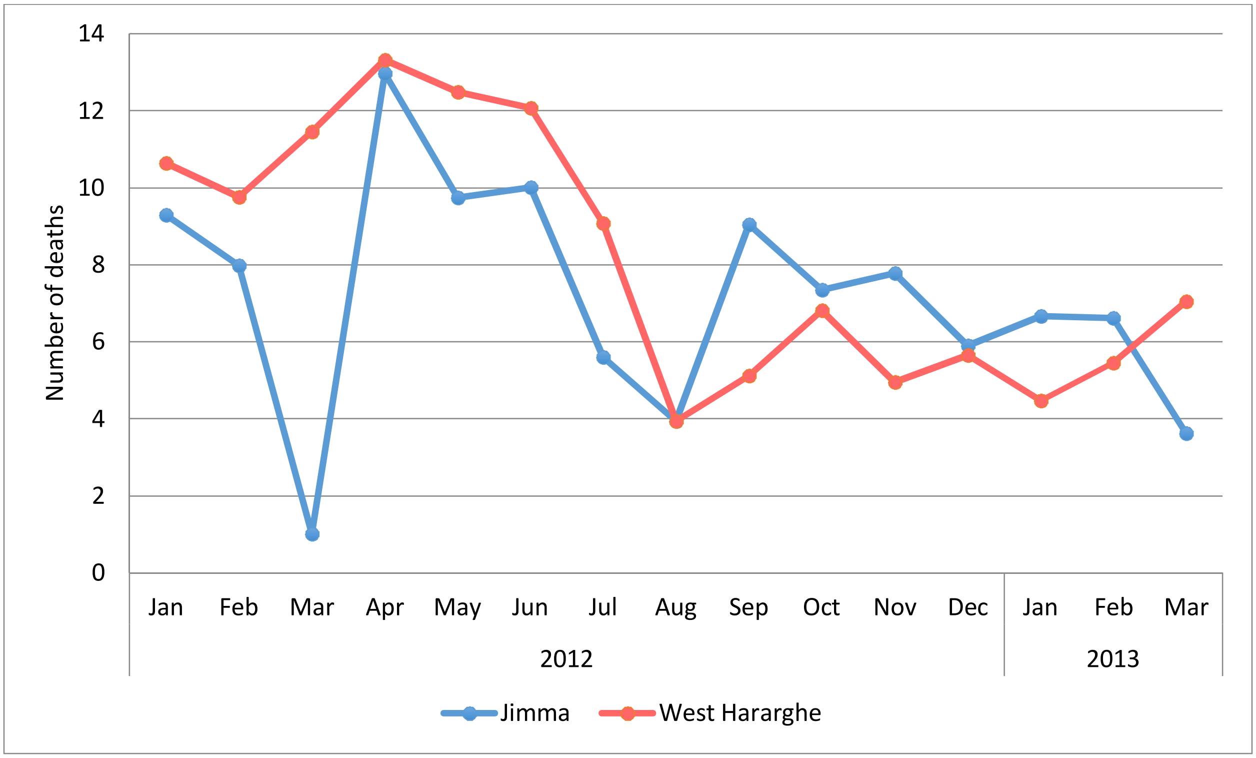

Reporting of data on deaths by HEWs: Neonatal deaths. The HEWs reported 153 neonatal deaths (i.e. deaths that occurred before the 28th day of life) during the validation period, 76 neonatal deaths in Jimma and 77 in West Hararghe. Figure 9 shows the reported deaths among infants in the two zones during the validation period. Similar to neonatal deaths, the number of infant deaths reported by month is subject to considerable month-to-month variability, which makes drawing conclusions about trends difficult. However, the general pattern of deaths being concentrated in the May-June period in Jimma and the April-July period for West Hararghe is similar to that for infant and under-five deaths.

Figure 9. **Neonatal deaths reported by HEWs in Jimma and West Hararghe, January 2012- March 2013**

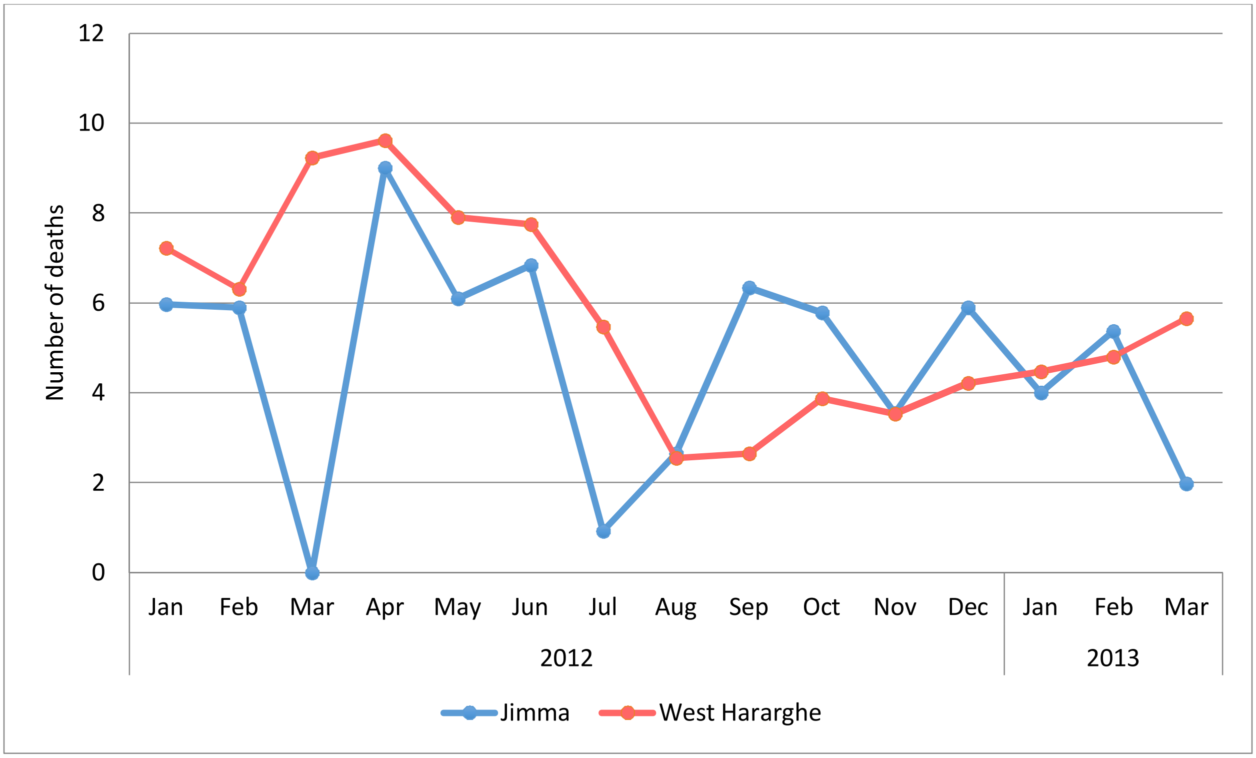

Internal data quality assessment: Sex ratio at birth. In most national populations, ratios of male versus female live births are expected to be between 102 and 107 males per 100 females.[15] Ratios that deviate from this standard can serve as an indicator of under-reporting of births for a particular gender. Figure 10 presents the sex ratio (males per 100 females) at birth by month in Jimma and West Hararghe. The monthly sex ratio at birth is subject to considerable month-to-month variability, as is expected given the small sample size. However, the overall sex ratio at birth was 100.2 males per 100 females for the combined population of both zones, 100.8 for Jimma zone and 100.5 for West Hararghe. Thus there may have been some slight under-reporting of male births by the HEWs, but the observed sex ratios at birth for the validation period do not indicate severe differential under-reporting of births by sex.

Figure 10. **Sex ratio at birth as reported by HEWs in Jimma and West Hararghe, January 2012- March 2013**

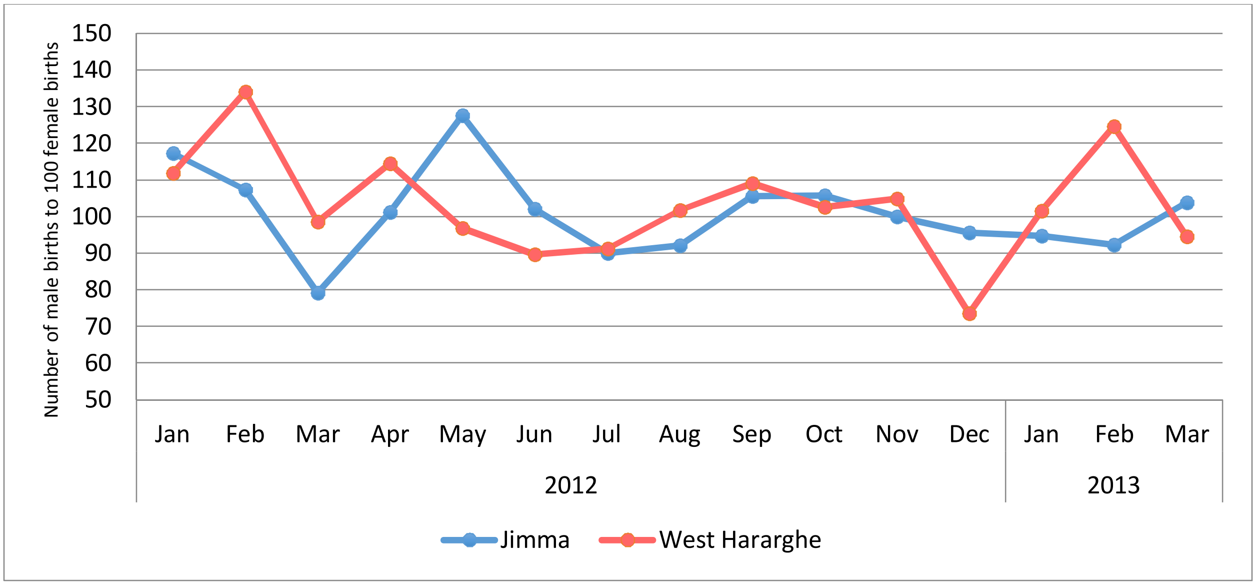

Internal data quality assessment: Age at death. Figure 11 and Figure 12 present the distribution of deaths by age for neonatal deaths and for children aged 1-23 months at death. As expected, the number of neonatal deaths is heavily concentrated around the first day of life and decline rapidly with increasing age. This pattern is consistent with what is observed in most human populations.

Figure 11. **Distribution of total HEW-reported neonatal deaths by age in days, January 2012- March 2013**

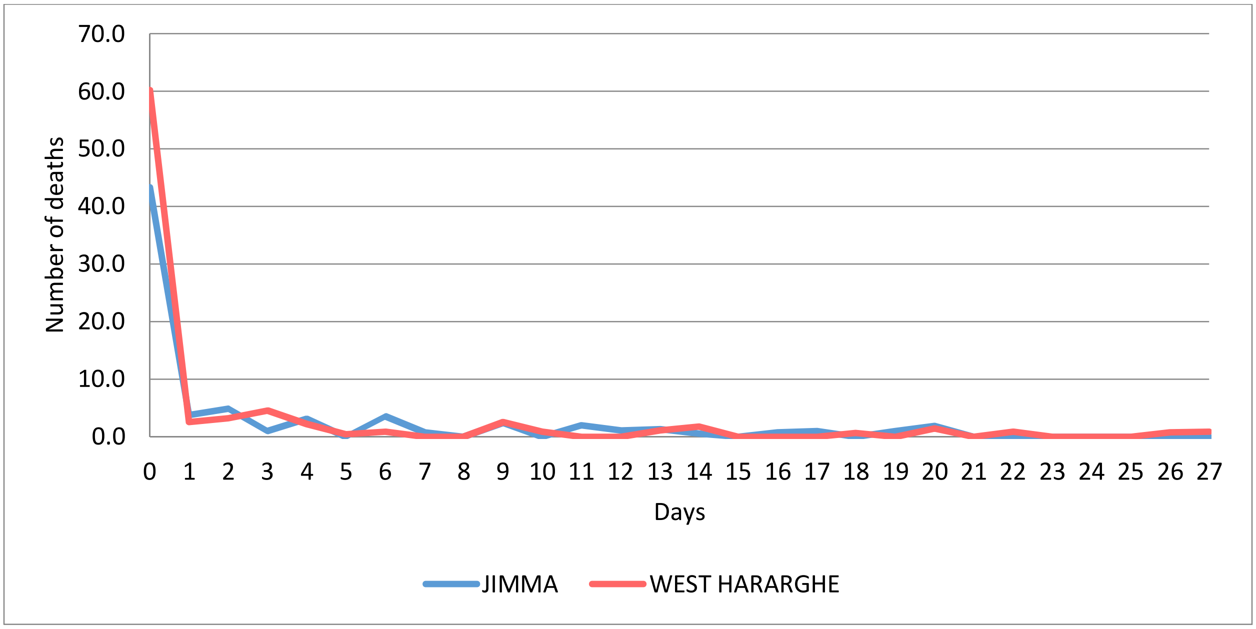

Figure 12 presents the age distribution of children who died between ages of 1 and 23 months for the HEW-based reporting for the validation period. For both Jimma and West Hararghe, the distribution is generally consistent with what is expected. Although the number of deaths is too small to make strong conclusions, there appear evidence of "age heaping," or rounding of the age at death, at ages of 6, 9, 18 and 21 months. However, even if this age heaping represents approximate reporting of ages at death, it is unlikely that the

imprecision in this age reporting will bias the estimation of infant mortality and mortality occurring after the first birthday. Age heaping at 12 months is usually the key indicator of such potential bias, and we do not observe any such age heaping at 12 months for HEW-based reporting from Jimma or West Hararghe.

Figure 12. Distribution of total HEW-reported children who died between ages of 1 month and 23 months by age at death in months, January 2012- March 2013

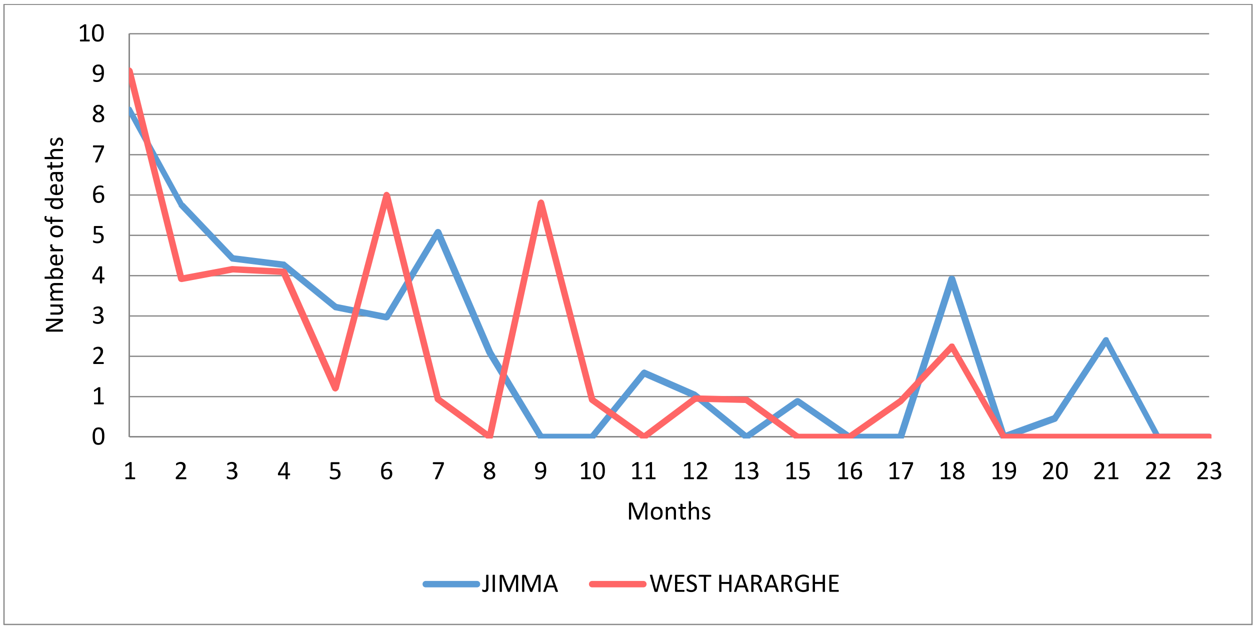

Table 11 shows the proportion of neonatal deaths that occurred in the first week of life, the proportion of infant deaths that occurred in the neonatal period, and the proportion of under-five deaths that occurred in the first year of life. The ratios observed based on HEW-based reporting in Jimma and West Hararghe appear slightly higher than typically observed of rural populations in low-resource settings. The ratio of early neonatal deaths (deaths between 0 days and 6 days) to neonatal deaths is an important indicator of data quality. The expected value is in the range of 70% to 80%. In West Hararghe, 86% of neonatal deaths reported occurred during the first week of life. This suggests that HEWs may be picking up proportionately more deaths during the first week of life. We observe a neonatal to infant death ratio of 0.677 for Jimma and West Hararghe based on HEW data, which is also slightly higher than the ratio of 0.627 reported in the 2011 Ethiopian DHS. The ratio of infant to under-five deaths in Jimma and West Hararghe reported by HEWs of 0.776 is about 15% higher than the ratio documented by the 2011 Ethiopian DHS. The patterns observed suggest that HEWs are reporting more deaths among the very young than the older children.

Table 11. Ratios of early neonatal to neonatal, neonatal to infant and infant to under-five deaths by zone, January 2012- March 2013

	Zone		
**Ratio**	**Jimma**	**West Hararghe**	**Total**
Early neonatal to neonatal deaths	0.7700	0.8606	0.8182
Neonatal to infant deaths	0.6535	0.6972	0.6767
Infant to under-5 deaths	0.7484	0.8006	0.7753

2.10.2. RMM community-based validation

In this section we present validation results by comparing HEW collected data to data captured in the endline validation survey. We consider the validation survey as a current best practice, and evaluate whether the community-based method produces results that are equivalent to the validation survey results.

HEW reporting validation: Total births and total deaths. Table 12 presents the total number of births and neonatal, infant and under-five deaths reported by HEWs for the two twelve-month validation periods. Tables and graphs in Annex 1 report the vital events reporting by HEWs by individual calendar months. The annual number of births in Jimma and West Hararghe documented by HEWs was fairly consistent across the two validation periods. Similarly, the number of neonatal, infant and under-five deaths documented by HEWs is relatively stable across the two validation periods for both zones.

Table 12. Number of births and deaths reported by HEWs for 12-month validation periods, Jan 2012 – Mar 2013

Annual Periods	No. of Births Reported by HEWs			No. of Deaths Reported by HEWs		
	Total	Males	Females	Neonatal	Infant	Under-five
**JIMMA**						
Jan 2012 - Dec 2012	2237	1122	1115	59	91	124
Apr 2012 - Mar 2013	2310	1161	1149	58	89	117
**WEST HARARGHE**						
Jan 2012 - Dec 2012	2724	1364	1360	70	105	132
Apr 2012 - Mar 2013	2652	1317	1335	63	90	115
**TOTAL**						
Jan 2012 - Dec 2012	4961	2486	2476	129	196	256
Apr 2012 - Mar 2013	4962	2477	2484	121	180	231

HEW reporting validation: Sex ratio at birth. In Table 13, we observe that the sex ratio at birth documented in both zones was around 100 males per female live births. These sex ratios at birth are fairly consistent with the range of 102-107 male births per 100 female births that would be expected for populations that do not practice sex selective abortion.[15] The ratios from HEWs collected data are also very close to those calculated from the endline survey.

Table 13. Sex-ratio at birth documented by HEWs and endline survey for 12-month validation periods, Jan 2012 – Mar 2013

Annual Periods	Sex Ratio at Birth (%)	
	HEW Reporting	Endline Survey
**JIMMA**		
Jan 2012 - Dec 2012	100.7	99.2
Apr 2012 - Mar 2013	101.0	101.4
**WEST HARARGHE**		
Jan 2012 - Dec 2012	100.2	102.4
Apr 2012 - Mar 2013	98.6	97.9
**TOTAL**		
Jan 2012 - Dec 2012	100.4	100.6
Apr 2012 - Mar 2013	99.7	99.8

HEW reporting validation: Age at death. Table 14 presents the ratio of neonatal to infant deaths and the ratio of infant to under-five deaths documented by the HEWs and those computed from the endline survey. These ratios are useful in assessing the age patterns of child deaths and identifying potential under-reporting of deaths within particular segments of the age range. We observe similar patterns for Jimma and West Hararghe. In both zones, HEWs documented around 66 neonatal deaths for every 100 hundred infant deaths documented. This was generally 6% higher than the ratio of neonatal to infant deaths estimated from the endline survey, which was approximately 60 per 100. This suggests that the HEWs reported proportionately more neonatal deaths than post-neonatal deaths as compared to the endline survey.

Similarly, for both zones, we observed a higher ratio of infant to under-five deaths documented by the HEWs compared with those reported in the endline survey. On average, the ratio of infant to under-five deaths documented by the HEWs was approximately 8% larger than what was estimated from the endline survey. The patterns indicate that HEWs have tendency to identify and report proportionately more deaths among younger than older children, compared to the endline survey.

Table 14. Ratios of Neonatal to Infant Deaths and Infant to Under-Five Deaths documented by HEWs & endline survey for 12-month validation periods, Jan 2012 – Mar 2013

Annual Periods	Ratio of Neonatal to Infant deaths (%)		Ratio of Infant to under-five deaths (%)	
	HEW Reporting	Endline Survey	HEW Reporting	Endline Survey
**JIMMA**				
Jan 2012 - Dec 2012	65.0	60.2	72.9	66.9
Apr 2012 - Mar 2013	65.6	63.8	76.4	65.6
**WEST HARARGHE**				
Jan 2012 - Dec 2012	66.8	56.6	79.8	68.6
Apr 2012 - Mar 2013	69.1	62.5	78.9	74.3
**TOTAL**				
Jan 2012 - Dec 2012	66.0	58.8	76.5	67.6
Apr 2012 - Mar 2013	67.3	63.2	77.6	69.2

HEW reporting validation: Expected versus reported events. In Table 15, we compare the total number of births and under-five deaths reported by HEWs to the expected number of births and under-five deaths based on the validation survey estimates and the census-based population size estimates for Jimma and West Hararghe. The ratios of birth and death events documented by HEWs to the expected number of births and deaths respectively based on the survey estimates were consistently low in both zones. The endline validation survey estimated approximately three times as many births and 4-5 times as many deaths as was documented by the HEW data collection process. These results are consistent with findings of the data quality assessment of the completeness of the HEW reporting presented in section 2.7 above. They also suggest notable limitations in the ability of HEWs to comprehensively report vital events routinely.

Both Jimma and West Hararghe are large rural areas with low population density. Jimma zone has a population density of 160 person per square kilometer and West Hararghe has a population density of 124 persons per square kilometer.[14] Thus HEWs, on average, need to cover relatively large local areas. As discussed in Section 2.7 above, HEWs were often overburdened with multiple tasks, sometimes absent from their woredas for extended periods, and were difficult to supervise. These factors are likely to have been key contributors to the HEW's modest coverage of vital events relative to that expected from the endline survey.

Table 15. Comparison of expected births and deaths (based on endline survey) and reported births and deaths by HEWs, for 12-month validation periods, Jan 2012 – Mar 2013

Annual Periods	Estimated Population of RMM Area	Estimated Crude Birth Rate (Endline Survey, per 1,000)	U5MR (Endline Survey, per 1,000)	Births			Under Five Deaths		
				Expected No. Births	No. Births Reported by HEWs	Ratio of Births reported by HEWs to Expected Births (%)	Expected No. of U5 Deaths	No. of U5 deaths reported by HEWs	Ratio of U5 reported by HEWs to Expected U5 deaths (%)
**JIMMA**									
Jan 2012 - Dec 2012	285559	27.2	84.6	7767	2237	28.8	657	124	18.9
Apr 2012 - Mar 2013	285559	26.4	81.2	7539	2310	30.6	612	117	19.1
**WEST HARARGHE**									
Jan 2012 - Dec 2012	223836	40.9	53.9	9155	2724	29.8	493	132	26.7
Apr 2012 - Mar 2013	223836	40.1	54.2	8976	2652	29.5	486	115	23.6
**TOTAL**									
Jan 2012 - Dec 2012	509395	32.7	69.1	16657	4962	29.8	1151	256	22.3
Apr 2012 - Mar 2013	509395	32.0	67.4	16301	4962	30.4	1099	231	21.1

HEW reporting validation: Mortality rates. Tables 16, 17, and 18 compare the under-five, infant and neonatal mortality rates respectively estimated using the HEW data and the endline validation survey.

When comparing the under-five mortality rate estimated from HEW data to that estimated from the endline survey data in Table 16, we observed that the HEW-based under-five mortality rate estimates were approximately 70% of the magnitude of those estimated by the endline survey. The ratios of HEW-based to endline survey under-five mortality rates for West Hararghe were notably higher (80-90%) than for Jimma (around 66%). Since the under-report of birth events and death events were of about similar magnitude for West Hararghe across the two validation periods, the HEW-based under-five mortality rate estimates appear equivalent with those estimated by the endline survey. This stems from the under-registration of birth events and the under-registration of death events by HEWs approximately cancelling each other out when calculating under-five mortality rates. This was not the case for Jimma where the degree of under-report of deaths by HEWs is higher than that of births, resulting in substantially higher under-estimation of the under-five mortality rate. The difference in the findings between Jimma and West Hararghe suggests non-stability in the degree of under-reporting of vital events by HEWs in different contexts. In both zones, the upper bound of the 95% confidence intervals of the ratio of the mortality rates suggests that the rates from HEWs collected data are equivalent to those measured from the validation survey at a 20% tolerance level.

Table 16. Under-five Mortality Rate estimates based on HEW data & expected Under-five Mortality rate estimates based on endline survey for 12-month validation periods, Jan 2012 – Mar 2013

Annual Periods	Under-Five Mortality Rate (per 1,000)					
	HEW data		Endline Survey		Ratio of Mortality Rates, HEW Data to Endline Survey	
	Rate	95% CI	Rate	95% CI	Ratio	95% CI
**JIMMA**						
Jan 2012 - Dec 2012	55.0	(47.1, 62.9)	84.6	(78.1, 91.1)	65.7	(50.2, 80.9)
Apr 2012 - Mar 2013	50.5	(43.5, 57.5)	81.2	(74.7, 87.6)	67.2	(50.0, 84.4)
**WEST HARARGHE**						
Jan 2012 - Dec 2012	48.4	(41.5, 55.3)	53.9	(49.1, 58.7)	89.8	(72.8,106.8)
Apr 2012 - Mar 2013	43.2	(36.7, 49.7)	54.2	(49.4, 59.0)	79.7	(61.2, 98.2)
**TOTAL**						
Jan 2012 - Dec 2012	51.7	(46.4, 57.0)	69.1	(65.1, 73.1)	74.8	(52.2, 97.4)
Apr 2012 - Mar 2013	46.6	(42.8, 51.4)	67.4	(63.4, 71.4)	69.1	(44.1, 94.1)

When we compared neonatal mortality rates and infant mortality rates estimated from the HEW data with those estimated from the endline survey, we observed similar results to those for under-five mortality rates. These results are shown in Table 17 and Table 18. Despite substantial under-registration of both birth events and neonatal and infant death events by HEWs relative to the endline survey, the HEW-based neonatal and infant mortality rates appeared equivalent to the rates from the endline survey in West Hararghe, judging from the 95% confidence intervals of the ratios.

Table 17. Infant Mortality Rate estimates based on HEW data & expected Infant Mortality rate estimates based on endline survey for 12-month validation periods, Jan 2012 – Mar 2013

Annual Periods	Infant Mortality Rate (per 1,000)					
	HEW data		Endline Survey		Ratio of Mortality Rates, HEW Data to Endline Survey	
	Rate	95% CI	Rate	95% CI	Ratio	95% CI
**JIMMA**						
Jan 2012 - Dec 2012	40.5	(30.6, 50.4)	56.6	(51.3, 61.9)	71.6	(48.8, 79.6)
Apr 2012 - Mar 2013	38.6	(31.6, 45.6)	53.3	(48.1, 58.5)	72.4	(38.7, 73.2)
**WEST HARARGHE**						
Jan 2012 - Dec 2012	38.7	(31.7, 45.7)	37.0	(33.0, 40.9)	104.6	(76.2, 111.0)
Apr 2012 - Mar 2013	34.1	(27.3, 59.4)	40.2	(36.1, 44.4)	84.8	(52.3, 90.0)
**TOTAL**						
Jan 2012 - Dec 2012	39.5	(34.3 44.7)	46.7	(43.4, 50.0)	84.6	(52.9, 98.7)
Apr 2012 - Mar 2013	36.2	(31.2, 41.2)	46.7	(43.3, 50.0)	77.5	(37.2, 87.8)

Our validation results indicate that community-based reporting has the potential to provide neonatal, infant and under-five mortality rate estimates. However these rates are systematically under-estimated compared to survey-based rates and the level of under-estimation is not consistent across the two zones. The HEW-based reporting substantially under-reported birth events and early childhood death events relative to retrospective reporting obtained via full birth histories. Although the level of vital events under-registration

was more severe in Jimma than West Hararghe, the level of under-registration was stable across the two validation periods.

Additional research is required to better understand the nature of vital events under-registration in Oromia region. In particular, additional data are required to assess whether the pattern of under-registration is relatively stable over time, or whether it is subject to considerable variability. In addition, record-level matching to compare the nature of events and individuals documented by in the full birth history but unobserved by the HEWs would help answer questions about the efficacy of HEW scale-up to improve vital events monitoring in rural Ethiopia.

Table 18. Neonatal Mortality Rate estimates based on HEW data & expected Neonatal Mortality rate estimates based on endline survey for 12-month validation periods, Jan 2012 – Mar 2013

Annual Periods	Neonatal Mortality Rate (per 1,000)					
	HEW data		Endline Survey		Ratio of Mortality Rates, HEW Data to Endline Survey	
	Rate	95% CI	Rate	95% CI	Ratio	95% CI
**JIMMA**						
Jan 2012 - Dec 2012	26.3	(19.5, 33.1)	34.0	(30.0, 38.2)	77.4	(59.3, 95.5)
Apr 2012 - Mar 2013	25.3	(19.0, 31.6)	34.0	(29.8, 38.1)	74.4	(50.9, 93.9)
**WEST HARARGHE**						
Jan 2012 - Dec 2012	25.8	(19.3, 31.9)	21.0	(18.0, 23.9)	122.9	(102.7, 143.1)
Apr 2012 - Mar 2013	23.6	(17.9, 29.3)	25.2	(21.9, 28.5)	93.7	(71.4, 109.6)
**TOTAL**						
Jan 2012 - Dec 2012	26.1	(22.5, 30.7)	27.4	(25.0, 30.0)	95.3	(68.7, 121.9)
Apr 2012 - Mar 2013	24.4	(20.2, 28.6)	29.5	(26.8, 32.1)	82.7	(66.2, 111.2)

In summary, these preliminary validation results from Ethiopia point to the feasibility of using HEWs to report on vital events. However, completeness of events identified and reported is very low and variable across zones. The resulting mortality rates are also under-estimated due to differential under-report of births and deaths. This first test indicated a need to understand and improve the coverage of vital events reporting – given the considerable under-registration of births and child deaths by HEWs. Given the low population density throughout much of rural Ethiopia, it is important to understand whether HEW-based vital events reporting is subject to considerable selection biases relative to full pregnancy history reporting via retrospective surveys. For example, do HEWs tend to only document events close to their home villages? Or is the under-reporting by HEWs approximately randomly distributed across time and space or at least predictable based on proxy indicators such as population density, seasonality, and other observable traits that can be used to reliably adjust HEW-based events reporting for under-registration? Interestingly, HEWs appears to pick-up proportionately more early deaths (neonatal and infant) relative to older deaths compared to full birth history-based mortality rates. Further studies should also focus on understanding this pattern of reporting.

REFERENCES

1 DFATD, Canada. The Initiative to Save a Million Lives. Available: http://www.pm.gc.ca/eng/news/2007/11/26/initiative-save-million-lives. Accessed 3 May 2014.

2 United Nations. End Poverty: Millennium Development Goals and Beyond 2015. Available: http://www.un.org/millenniumgoals/bkgd.shtml. Accessed 2 May 2013.

3 The DHS Program. Demographic and Health Surveys. Available: http://dhsprogram.com/. Accessed 3 May 2014.

4 UNICEF. Statistics and Monitoring: Multiple Indicator Cluster Survey. Available: http://www.unicef.org/statistics/index_24302.html. Accessed 3 may 2014.

5 WHO. Accountability for Women's and Children's Health. Available: http://www.who.int/woman_child_accountability/about/coia/en/. Accessed 3 May 2014.

6 The Institute for International Programs. (2007) Expert consultation on methodological alternatives for monitoring child mortality. Consultation Report. Baltimore, Maryland, USA: Johns Hopkins Bloomberg School of Public Health.

7 Federal Ministry of Health. 2011. Health and health related indicators. Policy Planning Directorate. Addis Ababa, Ethiopia.

8 World Bank. World Development Indicators. Available: http://data.worldbank.org/indicator. Accessed 3 May 2014.

9 The UN Inter-agency Group for Child Mortality Estimation, 2012; provided by SMS/DPS/UNICEF

10 Federal Ministry of Health. 2005. Health Sector Strategic Plan (HSDP III) 2005/6-2009/10. Planning and Programming Department, 2005

11 Federal Ministry of Health. 2010. Health Sector Development Plan IV 2010/11-2014/15. Federal Democratic Republic of Ethiopia. November 2010

12 FMOH, National Implementation Plan for Community-based Case Management of Common Childhood Illnesses. Draft 12 April 2010.

13 Legesse H, Degefie T, Hiluf M, et al. National scale-up of integrated community case Mmanagement in rural Ethiopia: Implementation and lessons learned. *Eth Med J* 2014; In press.

14 Federal Democratic Republic of Ethiopia. Population Census Commission. Summary and statistical report of the 2007 Population and Housing Census. December 2008. Addis Ababa, Ethiopia.

15 Hobbs FB. Age and sex composition. In Siegel JS and Swanson DA. The Methods and Materials of Demography. Second Edition. Elsevier Academic Press. 2004; Chapter 7: 125-174.
