# Supplementary material for: Using Health Extension Workers for Monitoring Child Mortality in Real-Time: Validation against Household Survey Data in Rural Ethiopia
Source: PLoS One. 2015 Nov 25;10(11):e0126909. doi: 10.1371/journal.pone.0126909 (PMC4659609; doi:10.1371/journal.pone.0126909)
Supplement: S2 Appendix — (PDF) [file pone.0126909.s002.pdf]

S2 APPENDIX

Appendices to RMM project report in Ethiopia

Appendix 2.1: Community-based (HEW) RMM method
Table 1.1. HEW reported births per month by gender and zone
Table 1.2. Sex ratio at birth by zone
Table 1.3. Number of under-5 deaths by month of death and zone
Table 1.4. Number of infant deaths by month of death and zone
Table 1.5. Number of neonatal deaths by month of death and zone
Table 1.6. U5MR by month by zone and gender
Table 1.7. Infant mortality rate by month by zone
Table 1.8. Neonatal mortality rate by month by zone and gender
Table 1.9. Age at death: 0-27 days
Table 1.10. Age at death: 1-23 months
Table 1.11. Age at death: 1-4 years
Appendix 2.2: Brief summary of endline validation survey data quality assessment
Table 2.1. Endline survey response rates for Households and Eligible Women by Zone
Table 2.2. Sex of the head of the household, number of under-five children and household size by zone
Table 2.3. Percentage distribution of women aged 15-49 years by number of children ever born by zone
Table 2.4. Reporting completeness of date of birth, age at death and child survival status by zone
Table 2.5. Age and Sex distribution of the household population by zone
Table 2.6. Number of births by survival status of child, year of birth, and zone
Figure 2.1. Population age pyramid for Jimma and West Hararghe
Figure 2.2. Distribution of females in sampled households by age and by zones
Table 2.7. Percentage distribution of interviewed women 15-49 by selected demographic characteristics and zone
Figure 2.3 Distribution of births by month and year of birth in West Hararghe zone
Figure 2.4. Distribution of births by month and year of birth in Jimma zone
Figure 2.5. Distribution of births by month and year of birth in both zones
Table 2.8. Distribution of births by calendar month in Jimma zone
Table 2.9. Distribution of births by calendar month in West Hararghe zone
Table 2.10. Distribution of births by calendar month in both zones
Figure 2.6. Sex Ratio at birth for births 2003-2012
Table 2.11. Sex ratio at birth by year and zone
Figure 2.7. Distribution of neonatal deaths by age at death in days (births 2003 – 2012)
Figure 2.8. Distribution of children who died between ages of 1 month and 23 months by age at death in month (births 2003 – 2012)
Table 2.12. Births in five years preceding past five years (2008-2012: Gregorian calendar): age at death 0-30 days
Table 2.13. Births in five years preceding past five years (2008-2012: Gregorian calendar): age at death 0-23 months
Table 2.14. Births in five years preceding past five years (2003-2007: Gregorian calendar): age at death 0-30 days
Table 2.15. Births in five years preceding past five years (2003-2007: Gregorian calendar): age at death 0-23 months
Table 2.16. Comparison of the ratio of early neonatal to neonatal deaths, neonatal to infant deaths, and infant to under-five deaths from the endline survey in Jimma and West Hararghe, January 2012 – March 2013
Appendix 2.3: RMM community-based validation

Table 3.1. Number of HEWs reporting by month and zone
Appendix 2.4: RMM Ethiopia questionnaires and forms
Form 4.1. Family Folder
Form 4.2. HEW Data Extraction Forms
Form 4.3. HEW Data Extraction Forms: English
Form 4.4. HEW Data Extraction Forms: Amharic

Real Time MORTALITY MONITORING
MONTHLY HEW RECORDING OF VITAL EVENTS DATA COLLECTION FORM
JOHNS HOPKINS UNIVERSITY – MIZ HASAB RESERCH CENTER

መረጃ መሰብሰቢያ ቅጽ
Guca odeeffannoon ittiin sasaabamu

ዞን ………………………… ወረዳ ………………………… Godina Aanaa	የቀበሌዉ ስም………………………… Maqaa gandaa
የጤና ተቋሙ ስም…………………………………………………… Maqaa dhaabata fayyaa	የጤና ተቋሙ መለያ ቁጥር ………………………… Koodii dhaabbata fayyaa
የጤና ኤክስቴንሽን ስም 1. ………………………… Maqaa hojjettu ekisteenshinii fayyaa 2. …………………………	ወር እና ዓ.ም. ………………………… Ji'aa fii bara

ክፍል ሀ: እርግዝና
Kutaa A: Ulfa

ተ.ቁ Lakk	የነፍሰ ጡሯ የቤተሰብ መለያ ቁ. ከቤተሰብ ማህደር Koodii maatii dubartii ulfaa galmmee maatii keessaa	የነፍሰ ጡሯ መለያ ቁ. ከቤተሰብ ማህደር Koodii dubartii ulfaa galmmee matii keessaa	ዕድሜ Umurii	የመጨረሻ የወር አበባ የታየበት ወር እና ዓ.ም. Guyyaa dhumaa adafiin itti mul'ate yoomi?	አሁን የስንት ወር ነፍሰ ጡር ነሽ Amma ulfa ji'a meeqati	*□እርግዝና ዉጤት * Bu'aa ulfichaa	አስተያየት ካለ Yaada (yoojiraate)
(ሀ 01)	(ሀ 02)	(ሀ 03)	(ሀ 04)	(ሀ 05)	(ሀ 06)	(ሀ 07)	(ሀ 08)
01				_ _ /_ _ _ _			

*** □እርግዝናዉ ዉጤት 1. እርግዝናዉ የተቋረጠ(Abortion) 2. የጨነገፈ (Miscarriage) 3. ሞተዉ የተወለዱ (Stillbirth) ከሆነ ይገለጽ::**

*** Bu'aa ulfichaa 1. Ulfichi kan addaan cite 2. Kan irraa ba'e 3. Du'anii kan dhalatan yoota'e ibsi.**

Appendix 2.1: Community-based (HEW) RMM method

Table 1.1. HEW reported births per month by gender and zone

	MALE BIRTHS REPORTED BY MONTH			FEMALE BIRTHS REPORTED BY MONTH		
**Month**	**Jimma**	**West Hararghe**	**Total**	**Jimma**	**West Hararghe**	**Total**
Jan_2012	60.6	70.7	131.3	51.7	63.2	114.9
Feb_2012	74.8	109.1	183.9	69.8	81.4	151.1
Mar_2012	90.7	107.1	197.8	114.7	108.7	223.4
Apr_2012	123.9	108.0	231.9	122.5	94.3	216.8
May_2012	122.6	136.1	258.7	96.1	140.6	236.7
Jun_2012	107.9	134.7	242.6	105.7	150.3	256.0
Jul_2012	89.2	139.8	229.0	99.2	153.3	252.5
Aug_2012	87.3	120.4	207.7	94.7	118.3	213.0
Sept_2012	82.5	122.2	204.7	78.2	112.0	190.2
Oct_2012	105.4	116.5	221.9	99.7	113.6	213.3
Nov_2012	87.9	104.6	192.5	87.9	99.8	187.7
Dec_2012	85.3	87.3	172.6	89.3	118.6	208.0
Jan_2013	106.9	67.1	173.9	112.8	66.1	178.9
Feb_2013	78.1	79.2	157.3	84.6	63.6	148.1
Mar_2013	77.1	89.9	167.0	74.3	95.2	169.5
Don't Know	3.1		3.1	0.0	0.5	0.5

Table 1.2. Sex ratio at birth by zone

Month	Zone		
	Jimma	West Hararghe	Total
Jan_2012	117.2	111.8	114.2
Feb_2012	107.2	134.1	121.7
Mar_2012	79.1	98.5	88.5
Apr_2012	101.2	114.5	107.0
May_2012	127.6	96.8	109.3
Jun_2012	102.1	89.6	94.8
Jul_2012	89.9	91.2	90.7
Aug_2012	92.1	101.8	97.5
Sept_2012	105.5	109.1	107.6
Oct_2012	105.8	102.6	104.1
Nov_2012	100.0	104.9	102.6
Dec_2012	95.5	73.6	83.0
Jan_2013	94.7	101.5	97.2
Feb_2013	92.3	124.6	106.2
Mar_2013	103.8	94.5	98.5

Table 1.3. Number of under-5 deaths by month of death and zone

Month	Zone		
	Jimma	West Hararghe	Total
Jan_2012	13.5	14.3	27.8
Feb_2012	10.2	12.3	22.6
Mar_2012	3.2	11.5	14.7
Apr_2012	18.7	14.5	33.3
May_2012	13.5	14.6	28.0
Jun_2012	14.9	15.9	30.8
Jul_2012	8.5	16.5	25.0
Aug_2012	5.6	4.5	10.1
Sept_2012	9.0	7.3	16.3
Oct_2012	10.0	8.1	18.0
Nov_2012	9.6	6.8	16.4
Dec_2012	7.6	5.7	13.3
Jan_2013	9.1	7.8	16.9
Feb_2013	6.6	5.5	12.1
Mar_2013	3.6	7.5	11.1
Total	143.7	152.7	296.4

Table 1.4. Number of infant deaths by month of death and zone

Month	Zone: Jimma	West Hararghe	Total
Jan_2012	9.3	10.6	19.9
Feb_2012	8.0	9.8	17.7
Mar_2012	1.0	11.5	12.5
Apr_2012	13.0	13.3	26.3
May_2012	9.7	12.5	22.2
Jun_2012	10.0	12.1	22.1
Jul_2012	5.6	9.1	14.7
Aug_2012	3.9	3.9	7.9
Sept_2012	9.0	5.1	14.2
Oct_2012	7.3	6.8	14.2
Nov_2012	7.8	4.9	12.7
Dec_2012	5.9	5.7	11.6
Jan_2013	6.7	4.5	11.1
Feb_2013	6.6	5.5	12.1
Mar_2013	3.6	7.0	10.7
Total	107.5	122.3	229.8

Table 1.5. Number of neonatal deaths by month of death and zone

Month	Zone: Jimma	West Hararghe	Total
Jan_2012	6.0	7.2	13.2
Feb_2012	5.9	6.3	12.2
Mar_2012	0.0	9.2	9.2
Apr_2012	9.0	9.6	18.6
May_2012	6.1	7.9	14.0
Jun_2012	6.8	7.7	14.6
Jul_2012	0.9	5.5	6.4
Aug_2012	2.6	2.5	5.2
Sept_2012	6.3	2.6	9.0
Oct_2012	5.8	3.9	9.6
Nov_2012	3.5	3.5	7.1
Dec_2012	5.9	4.2	10.1
Jan_2013	4.0	4.5	8.5
Feb_2013	5.4	4.8	10.2
Mar_2013	2.0	5.7	7.6
Total	70.3	85.3	155.5

Table 1.6. U5MR by month by zone and gender

Month	MONTHLY UNDER-FIVE MORTALITY RATE			MALE MONTHLY UNDER-FIVE MORTALITY RATE			FEMALE MONTHLY UNDER-FIVE MORTALITY RATE		
	Jimma	West Hararghe	Total	Jimma	West Hararghe	Total	Jimma	West Hararghe	Total
Jan_2012	120.1	106.9	112.9	113.7	123.1	118.7	61.3	60.1	60.6
Feb_2012	70.9	64.8	67.4	89.2	44.4	62.6	56.7	36.8	46.0
Mar_2012	15.6	52.8	34.7	81.0	92.0	87.0	59.0	45.0	52.2
Apr_2012	76.0	71.7	74.0	29.3	75.1	50.6	47.5	43.9	45.9
May_2012	60.9	52.6	56.3	54.8	76.4	66.2	20.3	16.2	17.9
Jun_2012	69.3	55.5	61.4	72.8	72.1	72.4	31.4	33.7	32.7
Jul_2012	45.0	56.2	51.9	19.6	46.2	35.9	9.3	15.7	13.2
Aug_2012	30.3	19.0	23.9	49.1	13.9	28.7	5.1	26.6	17.0
Sept_2012	56.3	31.0	41.3	71.2	63.3	66.5	65.2	40.2	50.5
Oct_2012	48.4	34.9	41.3	104.9	67.1	85.1	60.7	38.5	48.9
Nov_2012	54.2	32.3	42.3	102.9	69.9	84.9	0.0	0.0	0.0
Dec_2012	43.8	27.1	34.7	101.4	41.7	71.2	35.4	31.1	32.9
Jan_2013	41.1	57.0	47.2	34.1	7.1	23.7	13.4	36.9	22.1
Feb_2013	40.7	37.6	39.2	43.8	11.5	27.5	49.6	85.8	65.2
Mar_2013	23.7	40.2	32.8	10.4	36.9	24.7	15.9	22.7	19.7

Table 1.7. Infant mortality rate by month by zone

Month	MONTHLY INFANT MORTALITY RATE		
	Jimma	West Hararghe	Total
Jan_2012	82.8	79.5	81.0
Feb_2012	55.2	51.2	52.9
Mar_2012	4.8	52.8	29.4
Apr_2012	52.6	65.6	58.5
May_2012	44.1	45.1	44.7
Jun_2012	46.7	42.2	44.1
Jul_2012	29.7	31.0	30.5
Aug_2012	21.5	16.5	18.7
Sept_2012	56.3	21.9	35.9
Oct_2012	35.6	29.5	32.4
Nov_2012	44.0	23.4	32.8
Dec_2012	33.8	27.1	30.2
Jan_2013	30.1	32.5	31.1
Feb_2013	40.7	37.6	39.2
Mar_2013	23.7	37.7	31.4

Table 1.8. Neonatal mortality rate by month by zone and gender

	MONTHLY INFANT MORTALITY RATE		
**Month**	**Jimma**	**West Hararghe**	**Total**
Jan_2012	82.8	79.5	81.0
Feb_2012	55.2	51.2	52.9
Mar_2012	4.8	52.8	29.4
Apr_2012	52.6	65.6	58.5
May_2012	44.1	45.1	44.7
Jun_2012	46.7	42.2	44.1
Jul_2012	29.7	31.0	30.5
Aug_2012	21.5	16.5	18.7
Sept_2012	56.3	21.9	35.9
Oct_2012	35.6	29.5	32.4
Nov_2012	44.0	23.4	32.8
Dec_2012	33.8	27.1	30.2
Jan_2013	30.1	32.5	31.1
Feb_2013	40.7	37.6	39.2
Mar_2013	23.7	37.7	31.4

Table 1.9. Age at death: 0-27 days

Age in Days	Zone		
	Jimma	West Hararghe	Total
0	43.3	60.2	100.2
1	3.8	2.5	6.3
2	4.9	3.2	8.1
3	1.0	4.6	5.6
4	3.1	2.3	5.4
5	0.0	0.5	0.5
6	3.6	0.9	4.5
7	0.8	0.0	0.8
8	0.0	0.0	0.0
9	2.5	2.5	5.0
10	0.0	0.9	0.9
11	2.0	0.0	2.0
12	1.1	0.0	0.6
13	1.4	1.1	2.4
14	0.6	1.7	2.3
15	0.0	0.0	0.0
16	0.7	0.0	0.7
17	1.0	0.0	1.0
18	0.0	0.7	0.7
19	1.0	0.0	1.0
20	1.9	1.5	3.4
21	0.0	0.0	0.0
22	0.0	0.9	0.9
23	0.0	0.0	0.0
24	0.0	0.0	0.0
25	0.0	0.0	0.0
26	0.0	0.7	0.7
27	0.0	0.9	0.9

Table 1.10. Age at death: 1-23 months

	Zone		
Age in months	Jimma	West Hararghe	Total
1	8.1	9.1	15.5
2	5.8	3.9	9.7
3	4.4	4.2	8.6
4	4.3	4.1	8.3
5	3.2	1.2	4.4
6	3.0	6.0	8.9
7	5.1	0.9	5.5
8	2.1	0.0	2.1
9	0.0	5.8	5.8
10	0.0	0.9	0.9
11	1.6	0.0	1.6
12	1.0	0.9	2.0
13	0.0	0.9	0.9
15	0.9	0.0	0.9
16	0.0	0.0	0.0
17	0.0	0.9	0.9
18	3.9	2.2	6.1
19	0.0	0.0	0.0
20	0.5	0.0	0.5
21	2.4	0.0	2.4
22	0.0	0.0	0.0
23	0.0	0.0	0.0

Table 1.11. Age at death: 1-4 years

	Zozne		
Age in years	Jimma	West Hararghe	Total
1	2.8	3.8	11.2
2	1.9	3.8	13.8
3	3.5	2.8	7.9
4	1.6	1.9	13.9

Appendix 2.2: Brief summary of endline validation survey data quality assessment

This section presents the overall results and data quality measures from the endline validation survey with full birth history conducted from February-May, 2013.

Response Rate. For the household survey, we calculated a required sample size of 28,001 households, which included allowance for a non-response rate of 10%. Among these 28,001 sampled households, our interview field teams interviewed 27,782. In total, the response rate was 99.5%, with minimal differences between the two zones. For the birth history, 99.5% of all eligible women within the selected households completed that module of the survey instrument. Hence, as shown in Table 2.1, the level of non-response was negligible both in terms of selection of households and in terms of participation of eligible women.

Table 2.1. Endline survey response rates for Households and Eligible Women by Zone

	ZONE		
**Results**	**Jimma**	**West Hararghe**	**TOTAL**
Households selected	15,330	12,671	28,001
Interviewed households	15,316	12,556	27,872
**Household response rate**	99.9%	99.1%	99.5%
Eligible women in interviewed households	14,994	11,921	26,915
Women interviewed	14,946	11,845	26,791
**Women's response rate**	99.7%	99.4%	99.5%

Description of Sampled Households: Table 2.2 presents the distribution of head of household, household size, and number of under-five children per household by zone. Of the households sampled, 85.6% are headed by a male, have an average household size of 5.1 persons, and on average have 0.8 children aged under 5-years of age.

Table 2.2. Sex of the head of the household, number of under-five children and household size by zone

**Characteristics**	**Zone**		
	**Jimma**	**West Hararghe**	**Total**
**Head of Household**			
Male	85.1	86.2	85.6
Female	14.9	13.8	14.4
Total	100	100	100
**Household size**			
1	2.7	3.8	3.2
2	9.5	10.5	9.9
3	14.1	14.2	14.2
4	16.5	15.1	15.9
5	16.5	14.8	15.7
6	15	14.3	14.7
7	11.6	11.6	11.6
8	8	8.4	8.2

9	3.7	4.4	4
10 and above	2.4	2.9	2.7
Total	100	100	100
Mean household size	5.1	5.1	5.1
**Number of under-five children per household**			
0	46.3	42.3	44.5
1	32.7	30.7	31.8
2	19.1	23.4	21
3	1.9	3.4	2.6
4	0.1	0.2	0.1
5	*	*	*
Mean number of children under-five per household	0.8	0.9	0.8
**Number of households**	15,260	12,474	27,734

** The total households here are less than the one in Table 1 as there were 128 HH with "no consent"*

Table 2.3 presents the distribution of women aged 15-49 years for the two zones by their number of children ever born. In the two zones, women had 3.1 children on average. Of the sampled women aged 15-49 years old, 26.1% had not had any children.

Table 2.3. Percentage distribution of women aged 15-49 years by number of children ever born by zone

	**Zone**		
	**Jimma**	**West Hararghe**	**Total**
**Number of children**			
0	27.9	23.8	26.1
1	9.6	9.9	9.7
2	9.7	10.5	10
3	10.4	10.4	10.4
4	10.4	9.9	10.2
5	9.5	9.5	9.5
6	8.4	8.3	8.3
7	6	6.7	6.3
8	4.2	4.8	4.5
9	2.3	3.1	2.6
>=10	1.6	3.2	2.3
Missing	0	0	0
Total	100	100	100
Mean number of children ever born	3.1	3.4	3.3
**Number of households**	14,945	11861	26,806

Table 2.4 documents the reporting completeness of date of birth, age at death and child survival status information in the full pregnancy history disaggregated by zone. We observe that in both zones, the variable with the most amount of either unknown or missing information is the date of birth. Of the births reported in the survey in the two zones, for 19.5% of those in Jimma and 14.9% of those in West Hararghe the respondent did not report the exact month that the birth occurred. In contrast, we note that only a negligible number of reported births were missing the year of birth, both the year and month of birth, the survival status of the child or the age at death. This suggests that in both zones, the most notable reporting challenge for survey respondents was recalling of the timing of birth events.

Table 2.4. Reporting completeness of date of birth, age at death and child survival status by zone

Completeness of Date/Age information	Jimma		West Hararghe		Total	
	n	%	n	%	n	%
Unknown/missing month of birth	9,119	19.53	6,128	14.94	15,247	17.38
Unknown/missing year of birth	4	0.01	0	0	4	0
Missing both month and year of birth	4	0.01	0	0	4	0
Survival status unknown	1	0	3	0.01	4	0
Missing age at death	0	0	0	0	0	0

Table 2.5. Age and Sex distribution of the household population by zone

Age group	Jimma			West Hararge		
	Male	Female	Total	Male	Female	Total
0-4	15.2	15.2	15.2	17.3	17.8	17.5
5-9	18	18.5	18.2	19.9	20.3	20.1
10-14	15.5	15.9	15.7	14.6	14.8	14.7
15-19	9.7	8.5	9.1	9.4	8.7	9.1
20-24	6	6.5	6.3	6.3	6.9	6.6
25-29	5.9	7.5	6.7	6.4	8.2	7.3
30-34	5	5.6	5.3	5.6	5.8	5.7
35-39	5.2	5.4	5.3	5.3	4.7	5
40-44	4	3.3	3.7	4.2	2.6	3.4
45-49	3.2	2.3	2.7	2.9	1.7	2.3
50-54	2.3	4	3.1	1.9	2.7	2.3
55-59	2.3	2.9	2.6	1.4	2.2	1.8
60-64	2.5	2	2.3	1.6	1.6	1.6
65-69	1.9	1	1.5	1.2	0.9	1.1
70-74	1.5	0.7	1.1	1	0.5	0.7
75-80	0.8	0.3	0.6	0.4	0.2	0.3
80+	0.9	0.4	0.6	0.5	0.3	0.4
Missing	0	0	0	0	0	0
Total	100	100	100	100	100	100

Table 2.6. Number of births by survival status of child, year of birth, and zone

Year of birth	Jimma			West Hararghe			Total		
	Deaths	Living	Total	Deaths	Living	Total	Deaths	Living	Total
2004	286	2365	2651	245	2181	2426	531	4546	5077
2005	279	2662	2941	218	2415	2633	497	5077	5574
2006	293	2562	2855	220	2419	2639	513	4981	5494
2007	299	2761	3060	258	2402	2660	557	5163	5720
2008	243	2518	2761	202	2422	2624	445	4940	5385
2009	183	2444	2627	180	2277	2457	363	4721	5084
2010	181	2447	2628	161	2163	2324	342	4610	4952
2011	155	2025	2180	121	1938	2059	276	3963	4239
2012	118	2254	2372	105	2185	2290	223	4439	4662
2013	19	531	550	27	639	666	46	1170	1216
Total	5774	40918	46692	4990	36021	41011	10764	76939	87703

Population pyramid. Figure 2.1 displays the age-sex distribution of sampled households for the two study zones. We observe that the age-sex distribution of households in Jimma and West Hararghe are similar, and are typical of high fertility/high mortality populations in low-income countries. Both populations have very young populations in which approximately half of the current population is under 15 years old, around 16% of the population is under 5 years of age and only 6% of the population are 55 years or older.

Figure 2.1. Population age pyramid for Jimma and West Hararghe

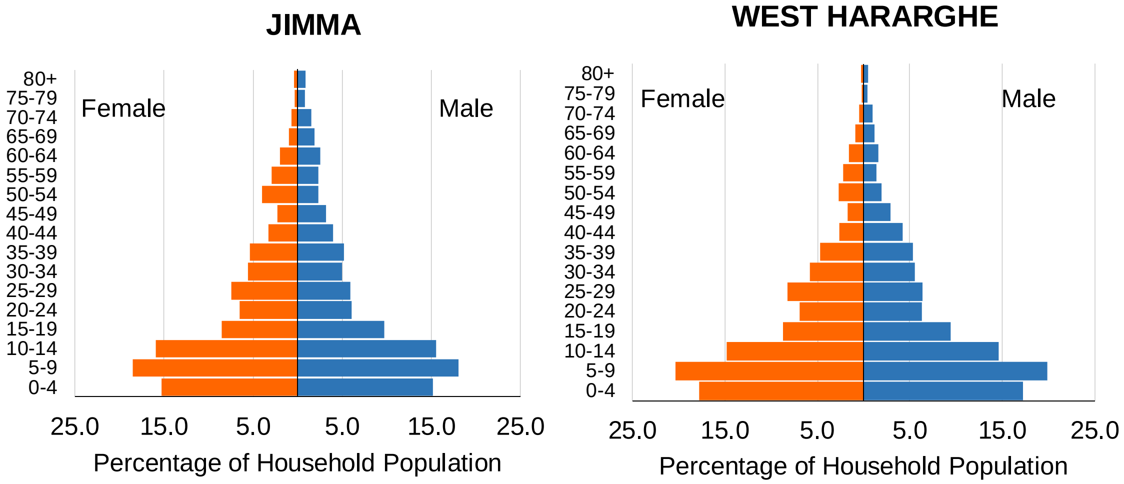

Figure 2.2 shows the age distribution of females aged 15 to 49 years in the sampled households who, by definition, were eligible to respond to the women's questionnaire. In both zones, approximately 60% of eligible women were between the ages of 15 and 29 years. The women who were interviewed had little formal education, with approximately 70% not having received any formal education and 26.5% having a primary level education. Of the sampled women aged 15-49, 80% were or had been married.

Figure 2.2. Distribution of females in sampled households by age and by zones

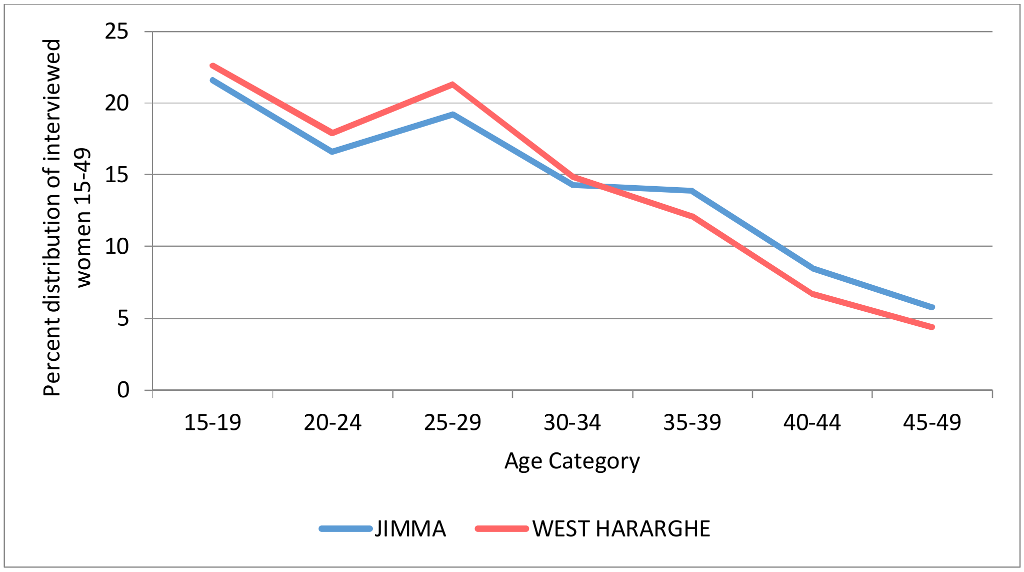

Table 2.7. Percentage distribution of interviewed women 15-49 by selected demographic characteristics and zone

		Zone	
	**Jimma**	**West Hararghe**	**Total**
**Age**			
15-19	21.6	22.6	22
20-24	16.6	17.9	17.2
25-29	19.2	21.3	20.2
30-34	14.3	14.9	14.6
35-39	13.9	12.1	13.1
40-44	8.5	6.7	7.7
45-49	5.8	4.4	5.2
Missing	0	0	0
Total	100	100	100
**Education**			
No education	65.3	76.1	70.1
Primary	30.6	21.4	26.5
Secondary	3	1.3	2.3
Secondary or more	0.8	0.6	0.7
Missing	0.3	0.6	0.5
Total	100	100	100
**Religion**			
Orthodox	9.9	8	9.1
Catholic	0.5	1.2	0.8
Protestant	4.1	0.2	2.4
Muslim	85.2	89.8	87.2
Traditional/animist	0	0	0
Other	0	0	0
Missing	0.3	0.6	0.5
Total	100	100	100
**Marital status**			
Married Once	67.5	78.6	72.4
Married More than once	9.8	4.9	7.6
Not married	22.3	15.9	19.5
Missing	0.3	0.6	0.5
Total	100	100	100
**Number of women 15-49**	**14,994**	**11,921**	**26,915**

__Data on births from the validation survey.__ Figures 2.4, 2.5 and 2.6 show the percent distribution of live births by month for Jimma, West Hararghe and both study zones, respectively for calendar years 2003-2012. We observe a notable amount of birth seasonality in the pattern of estimated births by month. In particular, over the course of the calendar year we generally observe a downward trend in estimated births whereby the magnitude of births tends to be higher for earlier months in the Gregorian calendar. However, the monthly trend by year shows no systematic outlying trends.

Figure 2.3 Distribution of births by month and year of birth in West Hararghe zone

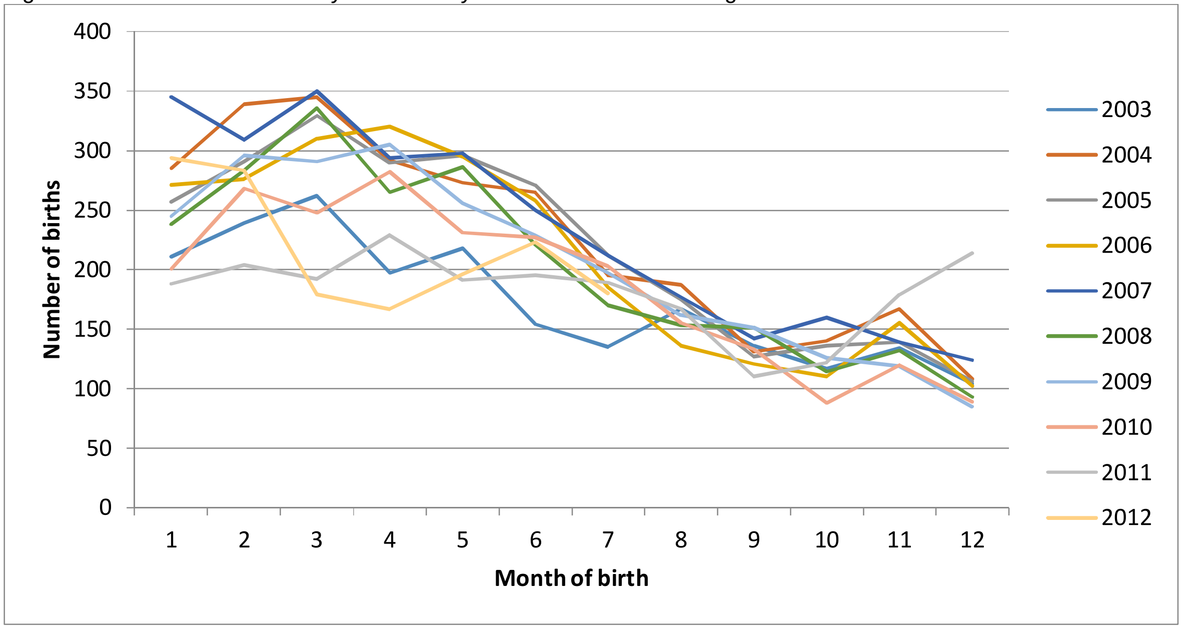

Figure 2.4. Distribution of births by month and year of birth in Jimma zone

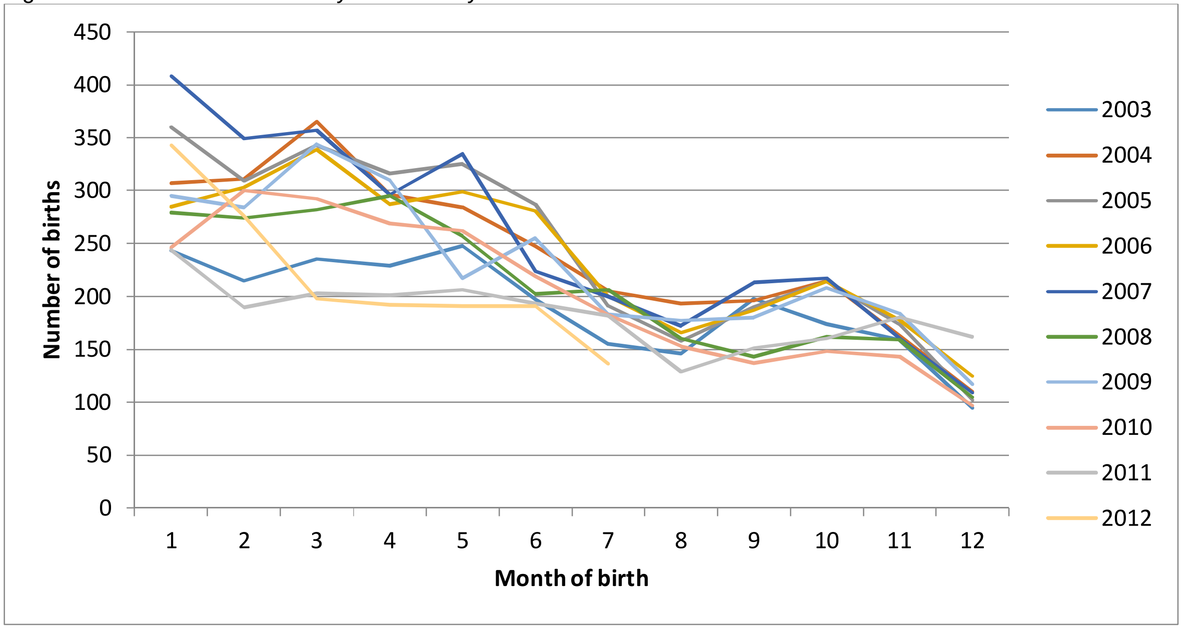

Figure 2.5. Distribution of births by month and year of birth in both zones

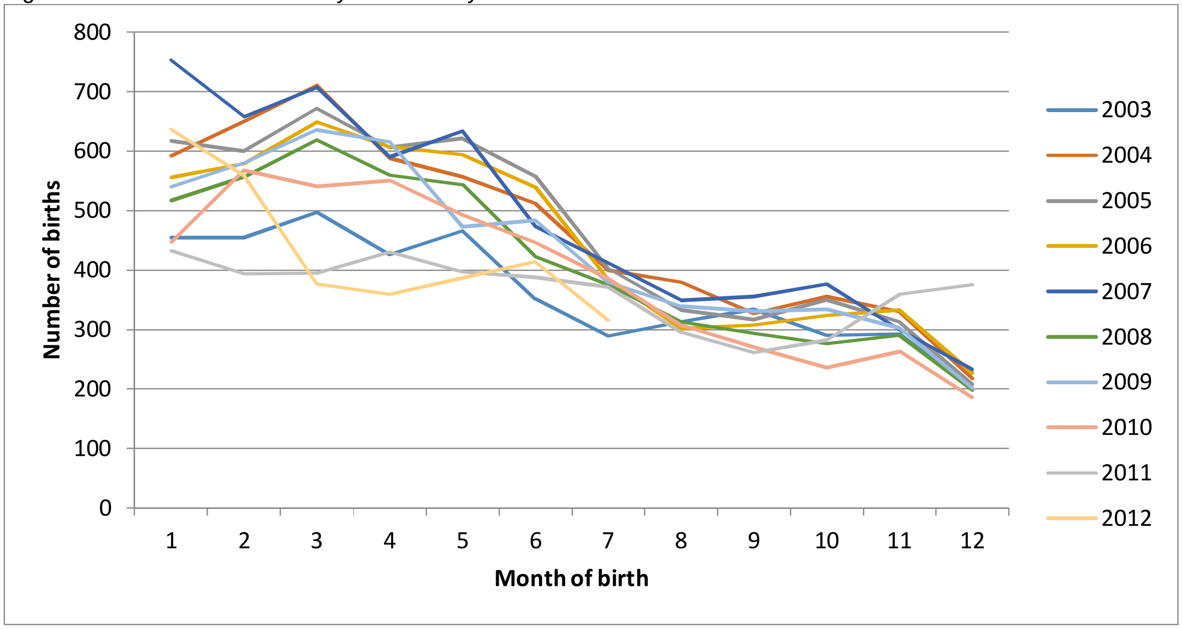

Table 2.8. Distribution of births by calendar month in Jimma zone

Year of birth	Month of birth												
	1	2	3	4	5	6	7	8	9	10	11	12	Total
2003	243	215	235	229	248	197	155	146	198	174	159	95	2294
2004	307	311	365	296	284	247	205	193	196	215	163	110	2892
2005	360	309	343	316	325	287	191	158	190	214	174	102	2969
2006	285	303	339	287	299	281	200	166	187	214	178	125	2864
2007	408	349	357	296	335	224	200	172	213	217	161	109	3041
2008	279	274	282	295	257	202	206	160	143	162	159	105	2524
2009	295	284	344	310	217	255	183	177	180	208	184	117	2754
2010	246	300	292	269	262	219	183	153	137	148	143	97	2449
2011	244	190	203	201	206	193	182	129	151	160	180	162	2201
2012	343	276	198	192	191	191	136						1559
Total	3010	2811	2958	2691	2624	2296	1841	1484	1596	1712	1501	1023	25547

Table 2.9. Distribution of births by calendar month in West Hararghe zone

Year of birth	Month of birth												
	1	2	3	4	5	6	7	8	9	10	11	12	Total
2003	211	239	262	197	218	154	135	167	136	117	134	104	2074
2004	285	339	345	292	273	265	195	187	131	140	167	108	2727
2005	257	291	329	290	296	271	212	175	127	136	139	106	2629
2006	271	276	310	320	295	258	185	136	121	110	155	102	2539
2007	345	309	350	294	298	250	212	177	142	160	139	124	2800
2008	238	283	336	265	286	221	170	153	151	114	132	93	2442
2009	245	296	291	305	256	229	197	162	151	126	119	85	2462
2010	201	268	248	282	231	227	203	155	133	88	120	89	2245
2011	188	204	192	229	191	195	189	167	110	122	179	214	2180
2012	294	283	179	167	196	223	180						1589

Total	2535	2788	2842	2641	2540	2293	1878	1545	1203	1113	1284	1025	23687

Table 2.10. Distribution of births by calendar month in both zones

Year of birth	Month of birth												
	1	2	3	4	5	6	7	8	9	10	11	12	Total
2003	454	454	497	426	466	351	290	313	334	291	293	199	4368
2004	592	650	710	588	557	512	400	380	327	355	330	218	5619
2005	617	600	672	606	621	558	403	333	317	350	313	208	5598
2006	556	579	649	607	594	539	385	302	308	324	333	227	5403
2007	753	658	707	590	633	474	412	349	355	377	300	233	5841
2008	517	557	618	560	543	423	376	313	294	276	291	198	4966
2009	540	580	635	615	473	484	380	339	331	334	303	202	5216
2010	447	568	540	551	493	446	386	308	270	236	263	186	4694
2011	432	394	395	430	397	388	371	296	261	282	359	376	4381
2012	637	559	377	359	387	414	316						
Total	5545	5599	5800	5332	5164	4589	3719	3029	2799	2825	2785	2048	49234

__Internal data quality assessment: Sex ratio at birth.__ The sex ratio at birth in normal populations is generally between 102 to 107 males per 100 females. Large departures from this range may suggest differential omission of births by sex. Figure 2.6 shows sex ratios at birth calculated as the number of males per 100 females. We observe that that for the 10 years preceding the survey, the sex ratio at birth is generally between 100 and 110 males per female. This suggests that there are no irregularities in the actual sex ratio at birth or its reporting in the two study zones. This observation is consistent with the estimated sex ratios at birth documented in the 2011 Ethiopia DHS.

Figure 2.6. Sex Ratio at birth for births 2003-2012

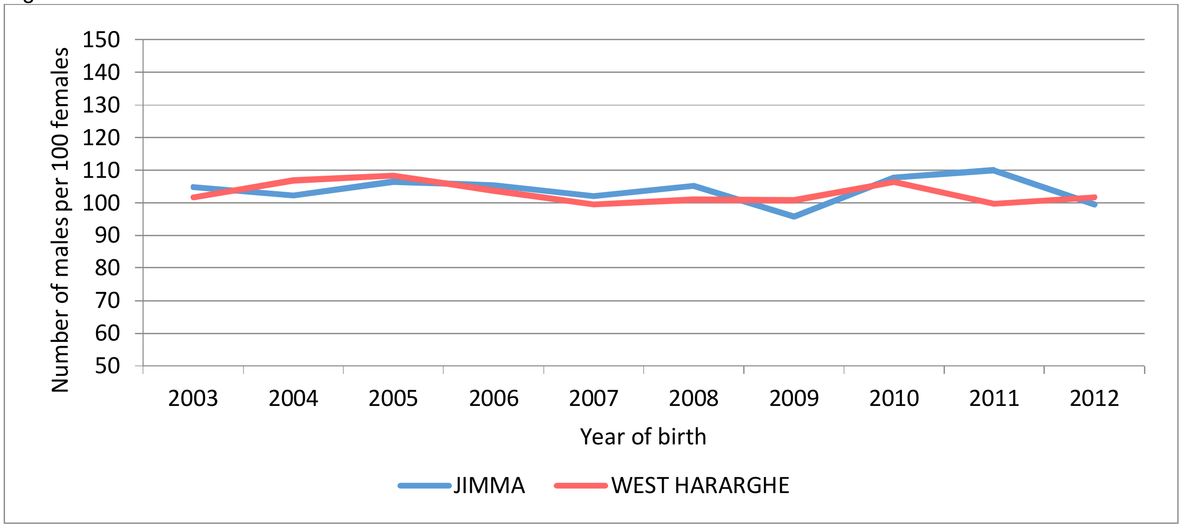

Table 2.11. Sex ratio at birth by year and zone

Year	Jimma			West Hararghe			Total		
	Males	Females	Total	Males	Females	Total	Males	Females	Total
1997	897	777	1674	804	655	1459	1701	1432	3133
1998	829	815	1644	647	628	1275	1476	1443	2919
1999	970	947	1917	803	807	1610	1773	1754	3527
2000	1138	1088	2226	944	879	1823	2082	1967	4049
2001	960	909	1869	844	771	1615	1804	1680	3484
2002	1431	1326	2757	1275	1238	2513	2706	2564	5270
2003	1174	1120	2294	1046	1028	2074	2220	2148	4368
2004	1462	1430	2892	1409	1318	2727	2871	2748	5619
2005	1531	1438	2969	1366	1262	2628	2897	2700	5597
2006	1469	1394	2863	1292	1247	2539	2761	2641	5402
2007	1536	1504	3040	1396	1404	2800	2932	2908	5840
2008	1294	1230	2524	1227	1215	2442	2521	2445	4966
2009	1346	1406	2752	1236	1226	2462	2582	2632	5214
2010	1269	1178	2447	1157	1088	2245	2426	2266	4692
2011	1151	1046	2197	1088	1091	2179	2239	2137	4376
2012	775	779	1554	801	788	1589	1576	1567	3143

Internal data quality assessment: Age at death. Another important indicator of the quality of data on under-five mortality is the distribution of age at death of reported neonatal and under-five deaths. Deaths among children under-five decline rapidly with increasing age, with largest number of deaths reported during the first few days of life. Errors in age reporting can affect mortality estimation. Figures 2.7 and 2.8 show the distribution of neonatal deaths by age in days and the per month distribution of children who died between the ages of one and 23 months by age at death. The observed distributions are typical of empirical surveys and the tendency for deaths to "heap" at certain ages. For age at death for neonates, these data show heaping at ages 5, 7, 10, 14, 15, 20, and 21 days, in addition to the expected heaping at ages 0 and 1 days. For children who died between the ages of one and 23 months, the distribution shows notable heaping at age 6, 12 and 18 months of age for both zones. If respondents round up their reporting of age in months, then this particular pattern would lead to an under-estimation of the infant mortality rate and an over-estimation of the child mortality rate. The under-five mortality rate will not be affected.

Figure 2.7. Distribution of neonatal deaths by age at death in days (births 2003 – 2012)

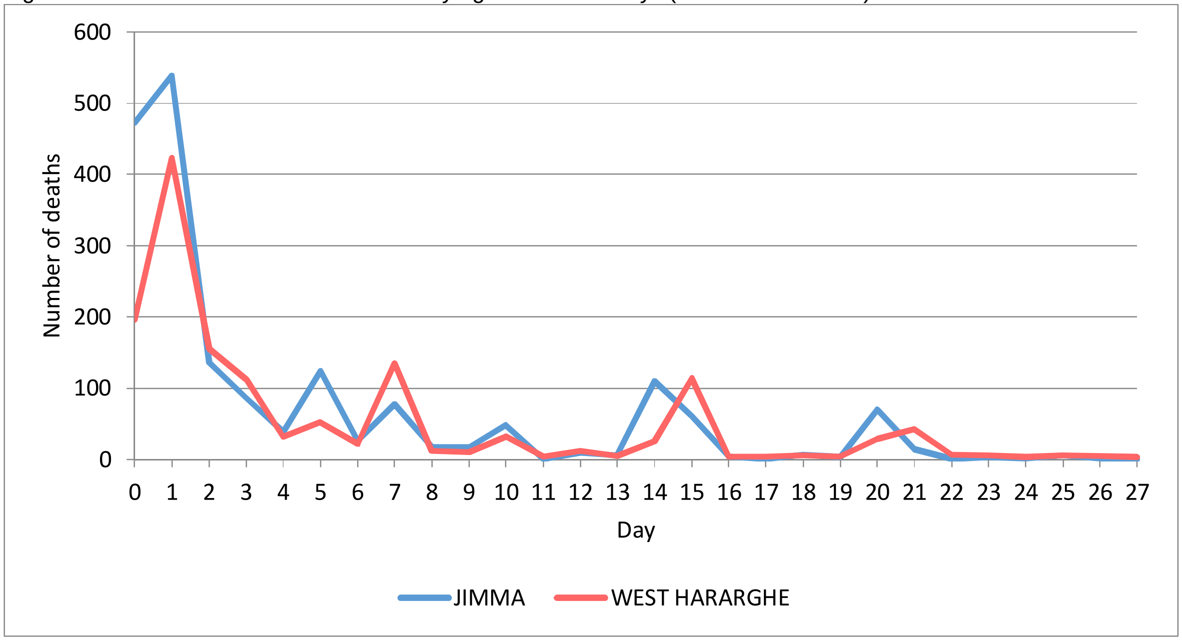

Figure 2.8. Distribution of children who died between ages of 1 month and 23 months by age at death in month (births 2003 – 2012)

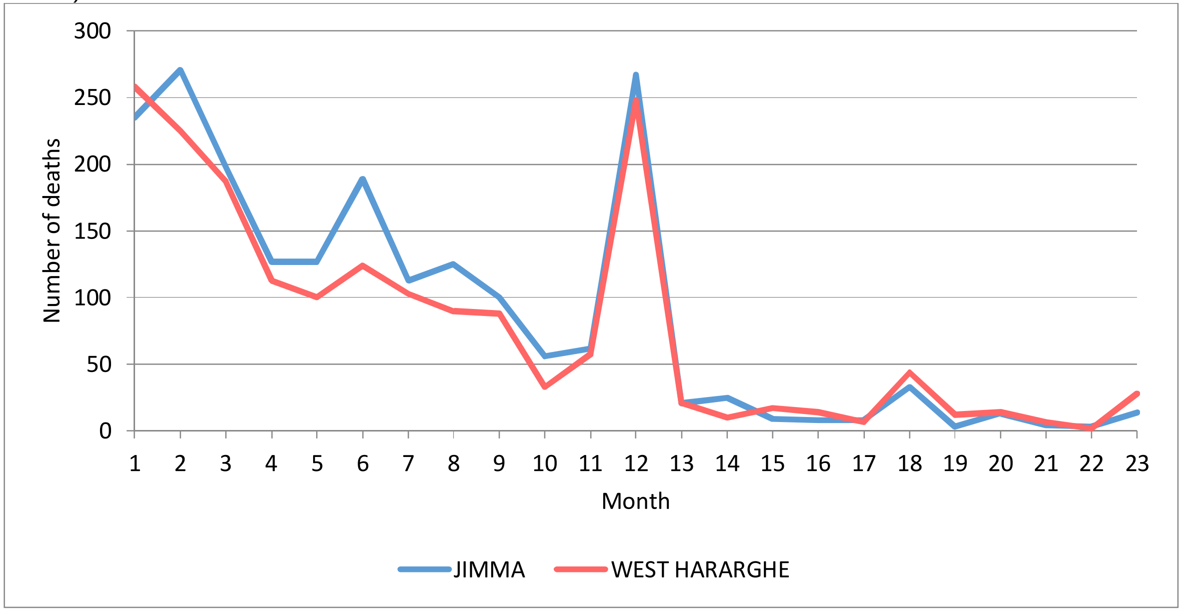

Table 2.12. Births in five years preceding past five years (2008-2012: Gregorian calendar): age at death 0-30 days

0-30 Days	Jimma	West Hararghe	Total
0	99	50	149
1	116	105	221
2	17	32	49
3	16	26	42
4	6	6	12
5	21	7	28
6	8	6	14

7	14	28	42
8	5	3	8
9	3	1	4
10	4	4	8
11	0	1	1
12	3	2	5
13	3	1	4
14	35	6	41
15	16	22	38
16	1	1	2
17	0	2	2
18	3	1	4
19	0	1	1
20	7	5	12
21	1	11	12
22	0	3	3
23	1	2	3
24	0	0	0
25	2	1	3
26	1	1	2
27	0	1	1
28	1	3	4
29	1	2	3
30	1	3	4
Total	385	337	722

Table 2.13. Births in five years preceding past five years (2008-2012: Gregorian calendar): age at death 0-23 months

**0-23 months**	**Jimma**	**West Hararghe**	**Total**
0	385	337	722
1	31	37	68
2	38	42	80
3	31	26	57
4	28	25	53
5	23	20	43
6	21	19	40
7	17	16	33
8	20	12	32
9	11	10	21
10	6	9	15
11	9	7	16
12	38	39	77
13	1	2	3
14	1	3	4
15	2	1	3
16	1	2	3
17	2	0	2
18	4	5	9
19	0	0	0
20	1	2	3
21	1	0	1
22	1	0	1
23	3	1	4
			0
Total	675	615	1290

Table 2.14. Births in five years preceding past five years (2003-2007: Gregorian calendar): age at death 0-30 days

0 – 30 days	Jimma	West Hararghe	Total
0	130	56	186
1	137	132	269
2	38	34	72
3	23	29	52
4	12	5	17
5	39	16	55
6	5	5	10
7	21	34	55
8	7	4	11
9	4	4	8
10	16	11	27
11	0	1	1
12	3	4	7
13	1	0	1
14	26	6	32
15	13	32	45
16	2	1	3
17	0	1	1
18	1	1	2
19	1	2	3
20	19	10	29
21	1	8	9
22	0	0	0
23	0	0	0
24	1	2	3
25	1	3	4
26	1	0	1
27	0	1	1
28	1	2	3
29	0	1	1
30	4	1	5
			0
Total	507	406	913

Table 2.15. Births in five years preceding past five years (2003-2007: Gregorian calendar): age at death 0-23 months

0-23 months	Jimma	West Hararghe	Total
0	913	507	1420
1	142	69	211
2	119	60	179
3	95	47	142
4	60	34	94
5	57	26	83
6	81	47	128
7	56	27	83
8	56	28	84
9	42	17	59
10	14	7	21
11	31	16	47
12	130	70	200
13	13	7	20
14	7	5	12

15	7	2	9
16	6	2	8
17	8	5	13
18	12	6	18
19	4	1	5
20	8	7	15
21	4	2	6
22	1	0	1
23	8	3	11
Total	1874	995	2869

Age patterns of deaths: ratio of early neonatal to neonatal deaths, neonatal deaths to infant deaths, and infant to under-five deaths. Table 2.16 presents the ratios of early neonatal to neonatal deaths, the ratio of neonatal to infant deaths, and the ratio of infant to under-five deaths from our validation survey and those from the 2011 DHS surveys for the Oromia region. The ratio of early neonatal to neonatal deaths is generally within the range of 0.70 to 0.75 for the 5-year period immediately preceding the survey and the earlier 5-year period 2003-2007.

The ratio of neonatal to infant deaths is approximately 0.6 for both zones for the five-years preceding the survey, and ranges from 0.3 to 0.46 for the period 2003-2007. This notable differential between the two recall periods suggests that when respondents retrospectively report neonatal deaths, their reports that refer to earlier years may be subject to relative under-reporting as a result of recall bias.

Table 2.16. Comparison of the ratio of early neonatal to neonatal deaths, neonatal to infant deaths, and infant to under-five deaths from the endline survey in Jimma and West Hararghe, January 2012 – March 2013

Ratio	Year						All deaths		
	2008-2012			2003-2007					
	Jimma	West Hararghe	Total	Jimma	West Hararghe	Total	Jimma	West Hararghe	Total
Early neonatal to neonatal deaths	0.7389	0.6988	0.7203	0.7535	0.6733	0.7178	0.7537	0.6762	0.7198
Neonatal to infant deaths	0.6177	0.5929	0.6059	0.3019	0.4565	0.3555	0.5393	0.5138	0.5278
Infant to Under-five deaths	.7955	.7971	.7962	.6960	.7338	.7133	.7346	.7589	.7157

Appendix 2.3: RMM community-based validation

Table 3.1. Number of HEWs reporting by month and zone

Month	Jimma	West Hararghe
Jan_2012	77	76
Feb_2012	77	76
Mar_2012	76	77
Apr_2012	75	78
May_2012	76	78
Jun_2012	72	75
Jul_2012	71	78
Aug_2012	74	78
Sept_2012	76	80
Oct_2012	77	80
Nov_2012	75	80
Dec_2012	66	80
Jan_2013	77	80
Feb_2013	78	80
Mar_2013	74	80

Appendix 2.4: RMM Ethiopia questionnaires and forms

Form 4.1. Family Folder

FMOH Logo

Federal Ministry of Health
Family Folder for the Health Extension Program

Region: ______________________ Woreda: ____________________ Kebele: ____________ Gote: ______________

Family number/House number __________________ Date of first registration ____/____/_________

Name of head of the family ________________________ Father _______________________Grand father __________________________ TIN____________

Household members' description

Individual ID	Name	Date of Birth	Place of Birth	Sex M/F	Occupation	**Marital status** S/M/D/W	Date died
		__/__/______					__/__/______
		__/__/______					__/__/______
		__/__/______					__/__/______
		__/__/______					__/__/______
		__/__/______					__/__/______
		__/__/______					__/__/______
		__/__/______					__/__/______
		__/__/______					__/__/______
		__/__/______					__/__/______
		__/__/______					__/__/______
		__/__/______					__/__/______

Household characteristics

Latrine

Latrine N / Y	Type	Date having latrine
		____/____/__________
		____/____/__________

Hand washing facility

Hand washing facility N / Y	Type	Date securing hand washing facility with soap/ash/sand
		____/____/__________
		____/____/__________

Waste disposal system

	Type	Date having waste disposal system
Solid waste disposal system available N / Y		____/____/__________
		____/____/__________
Liquid waste disposal system available N / Y		____/____/__________
		____/____/__________

Drinking water source

Drinking water source (type)

LLITN issued

ITN Issued Date	Number issued	Currently available enter date			
		Y/N	____/____/__________	Y/N	____/____/__________
		Y/N	____/____/__________	Y/N	____/____/__________

FMOH Ver 1/01

Region: ___________________________ Woreda: ______________________ Kebele: ______________ Gote: _________________

FMOH
Logo

Family number/House number _____________________ Date of first registration ____/____/__________

Name of head of the family ______________Father_____________________Grand father ___________________ TIN______________

HEP Packages training status

HEP packages	Training date started	Training date completed	Remark
Hygiene and Environmental Sanitation			
Excreta disposal	____/____/__________	____/____/__________	
Solid and liquid waste disposal	____/____/__________	____/____/__________	
Water supply and safety measures	____/____/__________	____/____/__________	
Food hygiene and safety measures	____/____/__________	____/____/__________	
Healthy home environment	____/____/__________	____/____/__________	
Control of insects and rodents	____/____/__________	____/____/__________	
Personal hygiene	____/____/__________	____/____/__________	
Family Health			
Maternal and Child health	____/____/__________	____/____/__________	
Family planning	____/____/__________	____/____/__________	
Immunization	____/____/__________	____/____/__________	
Nutrition	____/____/__________	____/____/__________	
Adolescent Reproductive health	____/____/__________	____/____/__________	
Disease Prevention and control			
HIV/AIDS, STI and TB prevention and control	____/____/__________	____/____/__________	
Malaria Prevention and control	____/____/__________	____/____/__________	
First Aid Emergency measures	____/____/__________	____/____/__________	
Health Education and Communication	____/____/__________	____/____/__________	

Household implementation status on the HEW packages

**Household activities**	Date	Remark
Registration	____/____/__________	
Training	____/____/__________	
Graduation	____/____/__________	
Advance Training	____/____/__________	

Notes

FMOH Logo

Federal Ministry of Health
Integrated Antenatal, Delivery, Postnatal and Newborn Care Card

**Pre Pregnancy status**		
**Individual ID Woman** ____________	Gravidity	
	Parity	
	LMP	____/___/_________
**New Born ID** ____________	EDD	____/___/_________
	Referred for STI testing (□)	
	Referred for HIV testing (□)	HIV test result R / NR / I

**Obstetric history**	**Remarks**
1. Previous stillbirth or neonatal loss? N/Y	
2. History of 3 or more consecutive spontaneous abortions? N/Y	
3. Birth weight of last baby < 2500g N/Y	
4. Birth weight of last baby > 4000g N/Y	
5. Hospitalization for hypertension or pre-eclampsia/eclampsia? N?Y	
6. Previous C/S N/Y	
**Current pregnancy**	
7. Age less than 16 years? N/Y	
8. Age more than 40 years? N/Y	
9. Height less than 150cm? N/Y	
10. Vaginal bleeding? N/Y	
11. Diastolic blood pressure 90mm Hg or more at booking? N/Y	
**General medical history**	
12. Diabetes mellitus? N/Y	
13. Renal disease? N/Y	
14. Cardiac disease? N/Y	
15. Known substance abuse? N/Y	
16. Any other severe medical disease or condition like malaria, TB, HIV: N/Y	

**Delivery**	
BP	
FHB	
Membrane (Intact /Ruptured)	
Delivery date and time	___/___/_______, ___:___
**Delivery Out come**	**Remarks**
Normal N/Y	
Complicated and referred N/Y	
Maternal death N/Y	
Birth Attendant	
Health Worker ☐	
HEW ☐	
TBA ☐	

**Newborn Outcome**	**Newborn care/ Remarks**
Live birth N/Y	
Sex (M/F)	
Weight in gram	
Still birth N/Y	
Neonatal death N/Y	
Dead (age in days)	

**Pregnancy follow up**				
Visits	1st	2nd	3rd	4th
Date of Visit	__/__/___	__/__/___	__/__/___	__/__/___
GA				
BP				
FHB				
Anemia/ Edema				
Sign/symptom of illness				
Action taken				
Folic acid				
Mebendazol				
**Remarks**				

**Postnatal care**			
Visits	1st	2nd	3rd
Date of visit	__/__/___	__/__/___	__/__/___
Sign/symptom of illness			
Action taken			
Referred			
Vitamin A for the mother			
Counseling on FP			
Counseling on exclusive breastfeeding			
Counseling on Breast feeding			
**Infant assessment**			
Weight			
Sign/symptom of illness			
Action taken			
**Remarks**			

FMOH Ver 1/01

FMOH
Logo

Name: __________________ **DOB:** ______________ **Individual ID:** _________________

Immunization						
Protected at birth against Tetanus (√)	Vaccine	**Date**	Vaccine	**Date**	Vaccine	**Date**
	BCG	___/___/______	OPV-2	___/___/______	Measles	
	OPV-0	___/___/______	Pentavalent-2	___/___/______		___/___/______
	OPV-1	___/___/______	OPV-3	___/___/______		
	Pentavalent-1	___/___/______	Pentavalent-3	___/___/______	Fully immunized by first birthday(√)	

Growth Monitoring					
Vitamin A(√)	Visits	1st	2nd	3rd	4th
	Date	___/___/______	___/___/______	___/___/______	___/___/______
	Weight in Kg				
	Height in cm				
	MUAC in cm				
	Action taken				

FMOH Ver 1/01

FMOH
Logo

Weight-for-age GIRLS

Birth to 5 years (z-scores)

World Health Organization

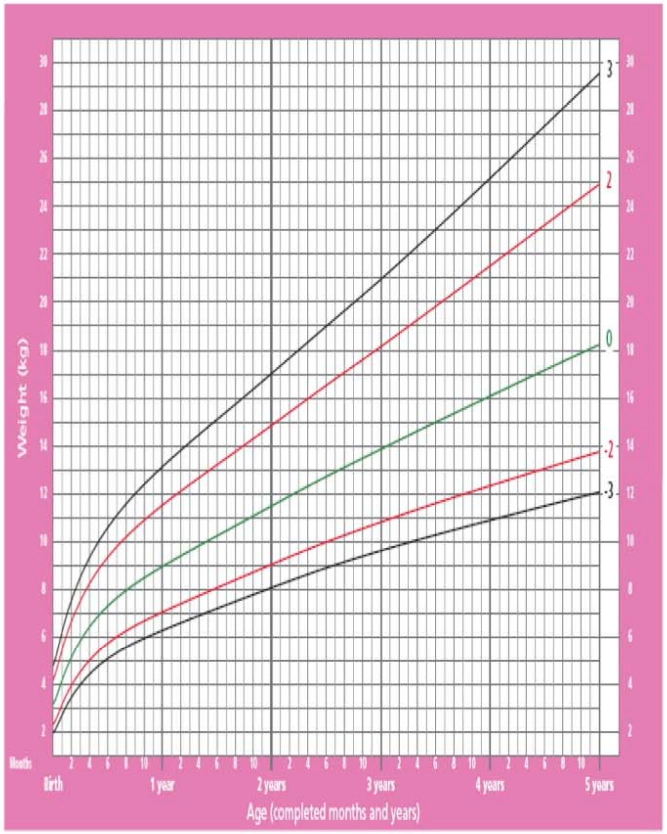

WHO Child Growth Standards

Length/height-for-age GIRLS

Birth to 5 years (z-scores)

World Health Organization

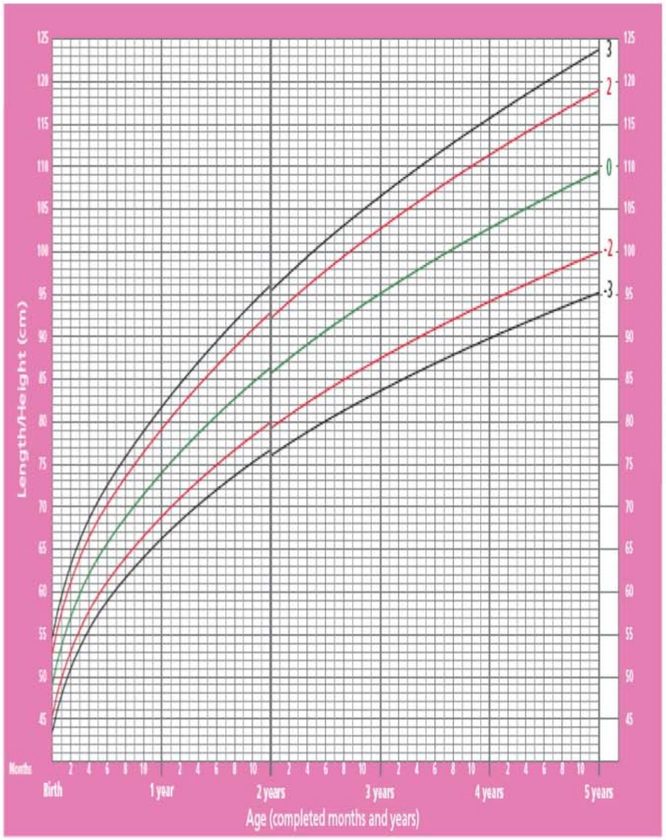

WHO Child Growth Standards

FMOH
Logo

Weight-for-age BOYS

Birth to 5 years (z-scores)

World Health Organization

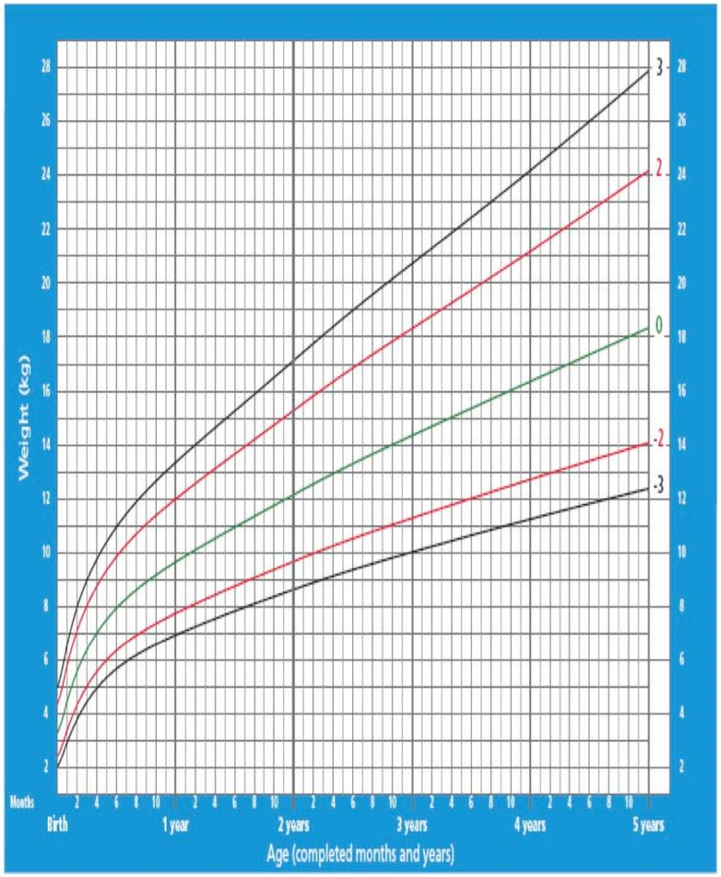

WHO Child Growth Standards

Length/height-for-age BOYS

Birth to 5 years (z-scores)

World Health Organization

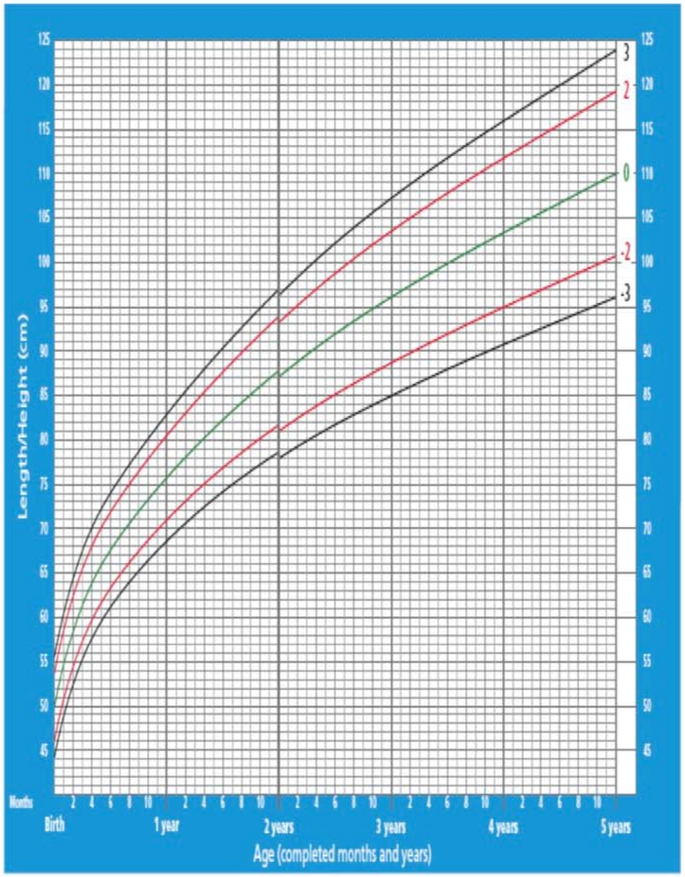

WHO Child Growth Standards

Health Card Page 1

Name: ____________________ DOB: _______________ Individual ID: ___________________

FMOH
Logo

Earlier health history:

**Disease information** (Children <5 yrs are listed with living mother)										
Individual ID	Date	Age	Sex	Diagnosis	Type of visit		Action taken	Setting (HP/ Home)	Referred (√)	Remark
					New	Repeat				
	___/___/_____									
	___/___/_____									
	___/___/_____									
	___/___/_____									
	___/___/_____									
	___/___/_____									
	___/___/_____									
	___/___/_____									
	___/___/_____									
	___/___/_____									
	___/___/_____									
	___/___/_____									

**Referral information**					
Individual ID	Reason for referral	Date of referral	Feed back	Date feed back received	Remark
		___/___/_____		___/___/_____	
		___/___/_____		___/___/_____	
		___/___/_____		___/___/_____	
		___/___/_____		___/___/_____	
		___/___/_____		___/___/_____	
		___/___/_____		___/___/_____	
		___/___/_____		___/___/_____	
		___/___/_____		___/___/_____	

N.B. Use the card independently for >= 5yrs and for < 5yrs who lost their mother

FMOH Ver 1/01

Health Card Page 2

Disease information (Children <5 yrs are listed with living mother)										
Individual ID	Date	Age	Sex	Diagnosis	Type of visit		Action taken	Setting (HP/ Home)	Referred (√)	Remark
					New	Repeat				
	__/__/____									
	__/__/____									
	__/__/____									
	__/__/____									
	__/__/____									
	__/__/____									
	__/__/____									
	__/__/____									
	__/__/____									
	__/__/____									
	__/__/____									
	__/__/____									

Referral information					
Individual ID	Reason for referral	Date of referral	Feed back	Date feed back received	Remark
		__/__/____		__/__/____	
		__/__/____		__/__/____	
		__/__/____		__/__/____	
		__/__/____		__/__/____	
		__/__/____		__/__/____	
		__/__/____		__/__/____	
		__/__/____		__/__/____	
		__/__/____		__/__/____	

N.B. Use the card independently for >= 5yrs and for < 5yrs who lost their mother

FMOH Ver 1/01

Health Card Page 3

FMOH
Logo

HIV/AIDS

Individual ID	Date	Currently on medication (ART) (Y/N)	Properly taking ART (Y/N)	Any related health problem identified	Remark
	___/___/______				
	___/___/______				
	___/___/______				
	___/___/______				
	___/___/______				
	___/___/______				
	___/___/______				
	___/___/______				
	___/___/______				
	___/___/______				
	___/___/______				
	___/___/______				
	___/___/______				
	___/___/______				
	___/___/______				
	___/___/______				
	___/___/______				
	___/___/______				
	___/___/______				
	___/___/______				
	___/___/______				
	___/___/______				
	___/___/______				
	___/___/______				

Tuberculosis

Individual ID	Date medication Started	Date	Any related health problem identified	Action taken	Date medication completed	Remark
		___/___/______				
		___/___/______				
		___/___/______				
		___/___/______				
	___/___/______	___/___/______			___/___/______	
		___/___/______				
		___/___/______				
		___/___/______				
		___/___/______				
	___/___/______	___/___/______			___/___/______	
		___/___/______				
		___/___/______				
		___/___/______				
		___/___/______				
	___/___/______	___/___/______			___/___/______	
		___/___/______				
		___/___/______				
		___/___/______				
		___/___/______				
	___/___/______	___/___/______			___/___/______	

FMOH Ver 1/01

Health card Page 4

Name: ________________ DOB: ______________ Individual ID: ________________

FMOH Logo

Family planning

	Description	Visit - 1	Visit – 2	Visit – 3	Visit – 4	Visit – 5	Visit – 6
1	New/ Repeat						
2	Visit Date	__/__/__	__/__/__	__/__/__	__/__/__	__/__/__	__/__/__
3	LMP	__/__/__	__/__/__	__/__/__	__/__/__	__/__/__	__/__/__
4	BP						
5	Weight						
6	Method						
7	Amount given						
8	Reason for method switch						
9	Reason for referral						
10	Next visit	__/__/__	__/__/__	__/__/__	__/__/__	__/__/__	__/__/__

	Description	Visit – 7	Visit – 8	Visit – 9	Visit – 10	Visit – 11	Visit – 12
1	New/Repeat						
2	Visit Date	__/__/__	__/__/__	__/__/__	__/__/__	__/__/__	__/__/__
3	LMP	__/__/__	__/__/__	__/__/__	__/__/__	__/__/__	__/__/__
4	BP						
5	Weight						
6	Method						
7	Amount given						
8	Reason for method switch						
9	Reason for referral						
10	Next visit	__/__/__	__/__/__	__/__/__	__/__/__	__/__/__	__/__/__

History of immunization

Individual ID	Antigen No	BCG (Date)	Polio (Date)	Pentavalent (Date)	Measles (Date)	TT (Date)		Remarks
	0		__/__/__			1	__/__/__	
	I		__/__/__	__/__/__		2	__/__/__	
	II		__/__/__	__/__/__		3	__/__/__	
	III		__/__/__	__/__/__		4	__/__/__	
	Single dose	__/__/__			__/__/__	5	__/__/__	

Individual ID	Antigen No	BCG (Date)	Polio (Date)	Pentavalent (Date)	Measles (Date)	TT (Date)		Remarks
	0		__/__/__			1	__/__/__	
	I		__/__/__	__/__/__		2	__/__/__	
	II		__/__/__	__/__/__		3	__/__/__	
	III		__/__/__	__/__/__		4	__/__/__	
	Single dose	__/__/__			__/__/__	5	__/__/__	

Height and weight

Individual ID	Date	Weight	Height	Remarks	Individual ID	Date	Weight	Height	Remarks

Orphan [When orphan is identified]

Individual ID	Age	Supports	Support received*						Remarks
			1st contact		Date	2nd contact		Date	
			Yes	No		Yes	No		
		Educational support			__/__/__			__/__/__	
		Food support			__/__/__			__/__/__	
		Shelter support			__/__/__			__/__/__	
		IGA support			__/__/__			__/__/__	
		Educational support			__/__/__			__/__/__	
		Food support			__/__/__			__/__/__	
		Shelter support			__/__/__			__/__/__	
		IGA support			__/__/__			__/__/__	

***** Document "Yes" or "No" based on the support received and put the date of support for orphans <15yrs.

FMOH Ver 1/01

Form 4.2. HEW Data Extraction Forms

RAPID MORTALITY MONITORING
HOUSEHOLD LISTING EXTRACTION FORM
JOHNS HOPKINS UNIVERSITY – MIZ HASAB RESERCH CENTER

**Woreda**: ………………………………………………………………………	**Kebele name**: ………………………………………………………………
**HEALTH FACILITY NAME**	**HEALTH FACILITY CODE**: ………………………………………………….
**NAME OF HEW 1**: ………………………………………………………………	**MONTH & YEAR**: ……………………………………………………………
**NAME OF HEW 2**: ……………………………………………………………	**Household ID number:** ………………………………………………….

SCHEDULE A: HOUSEHOLD LISTING

LINE NO	ID NO OF PERSON FROM FAMILY FOLDER	SEX	DATE OF BIRTH<br>DD/MM/YYYY	AGE	RELATIONSHIP WITH HEAD
(A01)	(A02)	(A03)	(A04)	(A05)	(A06)
		M F			
		M F			
		M F			
		M F			
		M F			
		M F			
		M F			
		M F			
9		M F			
10		M F			

CODES FOR RELATIONSHIP WITH HEAD

HEAD. . . . . ………………………….1
WIFE/HUSBAND . . . ……. …. . …………2
CHILD/ADOPTED CHILD ….. …………..3
GRANDCHILD. . . . . . ……….. …………4
NIECE/NEPHEW. . . ………. . …………5
FATHER/MOTHER. . ……… . . …………6
SISTER/BROTHER . . . …….. . ………….7
SON/DAUGHTER-IN-LAW … …………8
BROTHER/SISTER-IN-LAW……………..9
GRANDFATHER/MOTHER. . . ……….10
FATHER/MOTHER-IN-LAW. ………..11
OTHER RELATIVE. . . ……… ………….12
SERVANT OR SERVANT'S
RELATIVE . . . . . …………… ……………13
LODGER/LODGER'S
RELATIVE . . . . . ……………. …………..14
OTHER NON-RELATIVE. …. . …………15
OTHER (SPECIFY) . . ………. …………. 16

RAPID MORTALITY MONITORING
MONTHLY HEWs RECORDING OF VITAL EVENTS EXTRACTION FORM
JOHNS HOPKINS UNIVERSITY – MIZ HASAB RESERCH CENTER

**Zone:…………………Woreda**…………………………………………………………	**Kebele name**: …………………………………………………………
**HEALTH FACILITY NAME:** ………………………………………..	**HEALTH FACILITY CODE**: ………………………………………
**NAME OF HEW 1**: ………………………………………………	**MONTH & YEAR**: ……………………………………………………
**NAME OF HEW 2:** ………………………………………………	

SCHEDULE B: PREGNANCIES

LINE NO (B01)	HH ID NO OF WOMAN FROM FAMILY FOLDER (B02)	ID NO OF WOMAN FROM FAMILY FOLDER (B03)	AGE (B04)	LMP (month & year) (B05)	GESTATION IN MONTHS (B06)	*Pregnancy Outcome (B07)	COMENT IF NECESSARY (B08)
1				_ _ /_ _ _ _			
2				_ _ /_ _ _ _			
3				_ _ /_ _ _ _			
4				_ _ /_ _ _ _			
5				_ _ /_ _ _ _			
6				_ _ /_ _ _ _			
7				_ _ /_ _ _ _			
8				_ _ /_ _ _ _			
9				_ _ /_ _ _ _			
10				_ _ /_ _ _ _			
11				_ _ /_ _ _ _			
12				_ _ /_ _ _ _			
13				_ _ /_ _ _ _			
14				_ _ /_ _ _ _			
15				_ _ /_ _ _ _			

***Please indicate if pregnancy outcome is 1. Abortion 2. Miscarriage and 3. Stillbirth**

SCHEDULE C: BIRTHS, *Record only live births*

LINE NO (C01)	HH ID NO OF CHILD MOTHER FROM FAMILY FOLDER (C02)	ID NO OF CHILD MOTHER FROM FAMILY FOLDER (C03)	SEX OF CHILD (C04)	DATE OF BIRTH DD/MM/YYYY (C05)	PLACE OF BIRTH<br>Home With TBA=1<br>Home w/out TBA=2<br>H/C=3<br>Hospital=4<br>Other=5__<br>(C06)	COMMENT IF NECESSARY (C07)
1			M F	_ _ / _ _ /_ _ _ _		
2			M F	_ _ / _ _ /_ _ _ _		
3			M F	_ _ / _ _ /_ _ _ _		
4			M F	_ _ / _ _ /_ _ _ _		
5			M F	_ _ / _ _ /_ _ _ _		
6			M F	_ _ / _ _ /_ _ _ _		
7			M F	__ / __ / _ _ _ _		
8			M F	_ _ / _ _ / _ _ _ _		
9			M F	_ _ /_ _ / _ _ _ _		
10			M F	_ _/ _ _/ _ _ _ _		
11			M F	_ _/_ _ / _ _ _ _		

SCHEDULE D: DEATHS

LINE NO	HH ID NO OF PERSON FROM FAMILY FOLDER	*ID NO OF PERSON FROM FAMILY FOLDER	DATE OF BIRTH<br>DD/MM/YYYY	SEX	DATE OF DEATH<br>DD/MM/YYYY	AGE**	AGE GROUP<br>< 1yr = 1<br>1-4yrs = 2<br>≥ 5yrs = 3	PLACE OF DEATH<br>*(If health facility, write name)*	REPORTED CAUSE OF DEATH	***NOTE: ASK THE FOLLOWING FOR DEATHS OF WOMEN AGED 12 YEARS + TO PROBE IF MATERNAL DEATH***		
										Was (PERSON) Pregnant when she died?<br>Yes=1<br>No=2	Did (PERSON) die during Childbirth?<br>Yes=1<br>No=2	Did (PERSON) die within 2 months after delivery?<br>Yes=1<br>No=2
(D01)	(D02)	(D03)	(D04)	(D05)	(D06)	(D07)	(D08)	(D08)	(D09)	(D10)	(D11)	(D12))
1			_ _ /_ _ /_ _ _ _	M<br>F	_ _ /_ _ /_ _ _ _							
2			_ _ /_ _ /_ _ _ _	M<br>F	_ _ /_ _ /_ _ _ _							
3			_ _ /_ _ / _ _ _ _	M<br>F	_ _ /_ _ /_ _ _ _							
4			_ _ /_ _ / _ _ _ _	M<br>F	_ _ /_ _ /_ _ _ _							
**5**			_ _ /_ _ / _ _ _ _	M<br>F	_ _ /_ _ /_ _ _ _							

*** Include age at death in days if less than one month, in months if less than two years and in years if more than two years. In case age at death is in days or in months, please write days or months in brackets.*

**If NND and no ID number is given to child, please use mothers ID*

Real Time MORTALITY MONITORING
HEW RECORDING OF VITAL EVENTS DATA COLLECTION FORM
JOHNS HOPKINS UNIVERSITY – MIZ HASAB RESERCH CENTER

Data Collection Form

**Zone**: ………………………….Woreda:………………………………………..	**Kebele name**: …………………………………………………
**HEALTH FACILITY NAME:** …………………………………………..	**HEALTH FACILITY CODE**: ……………………………………
**NAME OF HEW 1**: …………………………………………………<br>2: …………………………………………………	**MONTH & YEAR**: ……………………………………………….

SCHEDULE B: BIRTHS, ***Record only live births***

LINE NO	HH ID NO OF CHILD MOTHER FROM FAMILY FOLDER	ID NO OF CHILD MOTHER FROM FAMILY FOLDER	SEX OF CHILD	DATE OF BIRTH<br><br>DD/MM/YYYY	PLACE OF BIRTH<br>Home TBA=1<br>Home no TBA=2<br>H/C=3<br>Hospital=4<br>Other=5	COMMENT IF NECESSARY
(B01)	(B02)	(B03)	(B04)	(B05)	(B06)	(B07)
			M F	_ _ / _ _ /_ _ _ _		
			M F	_ _ / _ _ /_ _ _ _		

Real Time MORTALITY MONITORING
MONTHLY HEW RECORDING OF VITAL EVENTS EXTRACTION FORM
JOHNS HOPKINS UNIVERSITY – MIZ HASAB RESERCH CENTER

Extraction Form

**Woreda**: …………………………………………………………………………..	**Kebele name**: …………………………………………………………………
**HEALTH FACILITY NAME:** …………………………………………..	**HEALTH FACILITY CODE**: ……………………………………………
**NAME OF HEW 1**: …………………………………………………<br>2: …………………………………………………	**MONTH & YEAR**: …………………………………………………….

SCHEDULE B: BIRTHS, ***Record only live births***

**LINE NO**	**HH ID NO OF CHILD MOTHER FROM FAMILY FOLDER**	**ID NO OF CHILD MOTHER FROM FAMILY FOLDER**	**SEX OF CHILD**	**DATE OF BIRTH**<br><br>**DD/MM/YYYY**	**PLACE OF BIRTH**<br>**Home TBA=1**<br>**Home no TBA=2**<br>**H/C=3**<br>**Hospital=4**<br>**Other=5**	**COMMENT IF NECESSARY**
**(B01)**	**(B02)**	**(B03)**	**(B04)**	**(B05)**	**(B06)**	**(B07)**
01			M F	_ _ / _ _ /_ _ _ _		
02			M F	_ _ / _ _ /_ _ _ _		
03			M F	_ _ / _ _ /_ _ _ _		
04			M F	_ _ / _ _ /_ _ _ _		
05			M F	_ _ / _ _ /_ _ _ _		
06			M F	_ _ / _ _ /_ _ _ _		

Real Time MORTALITY MONITORING
HEW RECORDING OF VITAL EVENTS DATA COLLECTION FORM
JOHNS HOPKINS UNIVERSITY – MIZ HASAB RESERCH CENTER

Data Collection Form

**Woreda**: ……………………………………………………………………….	**Kebele name**: …………………………………………………………
**HEALTH FACILITY NAME:** …………………………………………….	**HEALTH FACILITY CODE**: …………………………………………….
**NAME OF HEW 1**: ……………………………………………………<br>2: ……………………………………………………	**MONTH & YEAR**: ………………………………………………………

SCHEDULE B: PREGNANCIES

LINE NO	HH ID NO. OF WOMAN FROM FAMILY FOLDER	ID NO OF WOMAN FROM FAMILY FOLDER	AGE	LMP (month & year)	GESTATION IN MONTHS	PREGNANCY OUTCOME *	COMENT IF NECESSARY
(A 01)	(A02)	(A 03)	(A 04)	(A 05)	(A 06)	(A07)	(A 07)
				_ _ /_ _ _ _			
				_ _ /_ _ _ _			

*Please indicate if pregnancy outcome is 1. Abortion 2. Miscarriage and 3. Stillbirth

Real Time MORTALITY MONITORING
MONTHLY HEW RECORDING OF VITAL EVENTS EXTRACTION FORM
JOHNS HOPKINS UNIVERSITY – MIZ HASAB RESERCH CENTER

Extraction Form

**Zone**: ……………………………Woreda:……………………………………..	**Kebele name**: …………………………………………………………
**HEALTH FACILITY NAME:** ………………………………………..	**HEALTH FACILITY CODE**: …………………………………………
**NAME OF HEW 1**: …………………………………………………<br>2: …………………………………………………	**MONTH & YEAR**: ……………………………………………………

SCHEDULE B: PREGNANCIES

LINE NO	HH ID NO OF WOMAN FROM FAMILY FOLDER	ID NO OF WOMAN FROM FAMILY FOLDER	AGE	LMP (month & year)	GESTATION IN MONTHS	PREGNANCY OUTCOME *	COMENT IF NECESSARY
(A 01)	(A 02)	(A 03)	(A 04)	(A 05)	(A 06)	(A07)	(A 08)
				_ _ /_ _ _ _			
				_ _ /_ _ _ _			
				_ _ /_ _ _ _			
				_ _ /_ _ _ _			
				_ _ /_ _ _ _			
				_ _ /_ _ _ _			
				_ _ /_ _ _ _			
				_ _ /_ _ _ _			
				_ _ /_ _ _ _			
				_ _ /_ _ _ _			
				_ _ /_ _ _ _			
				_ _ /_ _ _ _			
				_ _ /_ _ _ _			

***Please indicate if pregnancy outcome is 1. Abortion 2. Miscarriage and 3. Stillbirth**

Real Time MORTALITY MONITORING
HEW RECORDING OF VITAL EVENTS DATA COLLECTION FORM
JOHNS HOPKINS UNIVERSITY – MIZ HASAB RESERCH CENTER

Data Collection Form

**Zone**: ……………………………Woreda:………………………………………	**Kebele name**: ……………………………………………………………
**HEALTH FACILITY NAME:** …………………………………………	**HEALTH FACILITY CODE**: …………………………………………
**NAME OF HEW 1**: …………………………………………………<br>2: …………………………………………………	**MONTH & YEAR**: ………………………………………………………

SCHEDULE C: DEATHS

LINE NO	HH ID NO OF PERSON FROM FAMILY FOLDER	ID NO OF PERSON FROM FAMILY FOLDER**	DATE OF BIRTH<br>DD/MM/YYYY	SEX	DATE OF DEATH<br>DD/MM/YYYY	AGE*	PLACE OF DEATH<br>*(If health facility, write name)*	REPORTED CAUSE OF DEATH	*NOTE: ASK THE FOLLOWING FOR DEATHS OF WOMEN AGED 12 YEARS + TO PROBE IF MATERNAL DEATH*		
									Was (PERSON) Pregnant when she died?<br>Yes=1<br>No=2	Did (PERSON) die during Childbirth?<br>Yes=1<br>No=2	Did (PERSON) die within 2 months after delivery?<br>Yes=1<br>No=2
(C01)	(C 02)	(C 03)	(C 04)	(C 05)	(C 06)	(C07)	(C 08)	(C 09)	(C 10)	(C 11)	(c 12)
			__/__/____	M F	__/__/____						
			__/__/____	M F	__/__/____						

**Include age at death in days if less than one month, in months if less than two years and in years if more than two years. In case age at death is in days or in months, please write days or months in brackets.*

***If NND and no ID number is given to child, please use mothers ID*

MONTHLY HEW RECORDING OF VITAL EVENTS EXTRACTION FORM

JOHNS HOPKINS UNIVERSITY – MIZ HASAB RESERCH CENTER

Extraction Form

Woreda: …………………………………………………………………………	Kebele name: ……………………………………………………………
HEALTH FACILITY NAME: …………………………………………	HEALTH FACILITY CODE: …………………………………………
NAME OF HEW 1: …………………………………………………<br>2: …………………………………………………	MONTH & YEAR: ………………………………………………………
	Household ID number: ………………………………………………

SCHEDULE C: DEATHS

LINE NO	HH ID NO OF PERSON FROM FAMILY FOLDER	ID NO OF PERSON FROM FAMILY FOLDER**	DATE OF BIRTH<br>DD/MM/YYYY	SEX	DATE OF DEATH<br>DD/MM/YYYY	AGE*	PLACE OF DEATH<br>*(If health facility, write name)*	REPORTED CAUSE OF DEATH	*NOTE: ASK THE FOLLOWING FOR DEATHS OF WOMEN AGED 12 YEARS + TO PROBE IF MATERNAL DEATH*		
									Was (PERSON) Pregnant when she died?<br>Yes=1<br>No=2	Did (PERSON) die during Childbirth?<br>Yes=1<br>No=2	Did (PERSON) die within 2 months after delivery?<br>Yes=1<br>No=2
(C01)	(C 02)	(C 03)	(C 04)	(C 05)	(C 06)	(C07)	(C 08)	(C 09)	(C 10)	(C 11)	(c 12)
01			__/__/____	M F	__/__/____						
02			__/__/____	M F	__/__/____						
03			__/__/____	M F	__/__/____						
04			__/__/____	M F	__/__/____						
05			__/__/____	M F	__/__/____						

Include age at death in days if less than one month, in months if less than two years and in years if more than two years. In case age at death is in days or in months, please write days or months in brackets. If NND and no ID number is given to child, please use mothers ID*

**CHECKED BY:: ……………………………………………… SIGNATURE ………………………………………………… DATE …………………………………………………………**

Form 4.4. HEW Data Extraction Forms: Amharic

Real Time MORTALITY MONITORING
MONTHLY HEW RECORDING OF VITAL EVENTS DATA COLLECTION FORM

JOHNS HOPKINS UNIVERSITY – MIZ HASAB RESERCH CENTER

መረጃ መሰብሰቢያ ቅጽ
Guca odeeffannoon ittiin sasaabamu

ዞን .... ወረዳ ....<br>Godina Aanaa	የቀበሌዉ ስም..................................<br>Maqaa gandaa
የጤና ተቋሙ ስም..<br>Maqaa dhaabata fayyaa	የጤና ተቋሙ መለያ ቁጥር ....<br>Koodii dhaabbata fayyaa
የጤና ኤክስቴንሽን ስም 1. ....<br>Maqaa hojjettu 2. ....<br>ekisteenshinii fayyaa	ወር እና ዓ.ም. ....<br>Ji'aa fii bara

ክፍል ሀ፡ እርግዝና
Kutaa A: Ulfa

ተ.ቁ<br>Lakk	የነፍሰ ጡሯ የቤተሰብ መለያ ቁ. ከቤተሰብ ማህደር<br>Koodii maatii dubartii ulfaa galmmee maatii keessaa	የነፍሰ ጡሯ መለያ ቁ. ከቤተሰብ ማህደር<br>Koodii dubartii ulfaa galmmee matii keessaa	ዕድሜ<br>Umurii	የመጨረሻ የወር አበባ የታየበት ወር እና ዓ.ም.<br>Guyyaa dhumaa adafiin itti mul'ate yoomi?	አሁን የስንት ወር ነፍሰ ጡር ነሽ<br>Amma ulfa ji'a meeqati	*□እርግዝና ዉጤት<br>* Bu'aa ulfichaa	አስተያየት ካለ<br>Yaada (yoojiraate)
(ሀ 01)	(ሀ 02)	(ሀ 03)	(ሀ 04)	(ሀ 05)	(ሀ 06)	(ሀ 07)	(ሀ 08)
01				_ _ /_ _ _ _			

* □እርግዝናዉ ዉጤት 1. እርግዝናዉ የተቋረጠ(Abortion) 2. የጨነገፈ (Miscarriage) 3. ሞተዉ የተወለዱ (Stillbirth) ከሆነ ይገለጽ።

* Bu'aa ulfichaa 1. Ulfichi kan addaan cite 2. Kan irraa ba'e 3. Du'anii kan dhalatan yoota'e ibsi.

Real Time MORTALITY MONITORING
MONTHLY HSA RECORDING OF VITAL EVENTS EXTRACTION FORM

JOHNS HOPKINS UNIVERSITY – MIZ HASAB RESERCH CENTER

መረጃዎች ሁሉ መሰብሰቢያ ቅጽ

Guca odeeffannoon ittiin sasaabamu

ዞን ……………………… ወረዳ ………………………<br>Godina Aanaa	የቀበሌዉ ስም………………………<br>Maqaa gandaa
የጤና ተቋሙ ስም………………………<br>Maqaa dhaabata fayyaa	የጤና ተቋሙ መለያ ቁጥር<br>………………………<br>Koodii dhaabbata fayyaa
የጤና ኤክስቴንሽን ስም 1. ………………………<br>Maqaa hojjettu ekisteenshinii fayyaa 2. ………………………	ወር እና ዓ.ም. ………………………<br>Ji'aa fii bara

ክፍል ሀ: እርግዝናዎች

Kutaa A: Ulfa

ተ.ቁ Lakk	የነፍሰ ጡሯ የቤተሰብ መለያ ቁ. ከቤተሰብ ማህደር Koodii maatii dubartii ulfaa galmmee maatii keessaa	የነፍሰ ጡሯ መለያ ቁ. ከቤተሰብ ማህደር Koodii dubartii ulfaa galmmee matii keessaa	ዕድሜ Umurii	የመጨረሻ የወር አበባ የታየበት ወር እና ዓ.ም. Guyyaa dhumaa adafiin itti mul'ate yoomi?	አሁን የስንት ወር ነፍሰ ጡር ነሽ Amma ulfa ji'a meeqati	*እርግዝናዉ ዉጤት * Bu'aa ulfichaa	አስተያየት ካለ Yaada (yoojiraate)
(ሀ 01)	(ሀ 02)	(ሀ 03)	(ሀ 04)	(ሀ 05)	(ሀ 06)	(ሀ 07)	(ሀ 08)
01				_ _ /_ _ _ _			
02				_ _ /_ _ _ _			
03				_ _ /_ _ _ _			
04				_ _ /_ _ _ _			
05				_ _ /_ _ _ _			
06				_ _ /_ _ _ _			
07				_ _ /_ _ _ _			
08				_ _ /_ _ _ _			
09				_ _ /_ _ _ _			
10				_ _ /_ _ _ _			
11				_ _ /_ _ _ _			
12				_ _ /_ _ _ _			
13				_ _ /_ _ _ _			

* እርግዝናዉ ዉጤት 1. እርግዝናዉ የተቋረጠ(Abortion) 2. የጨነገፈ (Miscarriage) 3. ሞተዉ የተወለዱ (Stillbirth) ከሆነ ይገለጽ::

*** Bu'aa ulfichaa 1. Ulfichi kan addaan cite 2. Kan irraa ba'e 3. Du'anii kan dhalatan yoota'e ibsi.**

Real Time MORTALITY MONITORING
MONTHLY HEW RECORDING OF VITAL EVENTS DATA COLLECTION FORM
JOHNS HOPKINS UNIVERSITY – MIZ HASAB RESERCH CENTER

መረጃ መሰብሰቢያ ቅጽ
Guca odeeffannoon ittiin sasaabamu

ዞን .... ወረዳ .... Godina Aanaa	የቀበሌዉ ስም................................... Maqaa gandaa
የጤና ተቋሙ ስም... Maqaa dhaabata fayyaa	የጤና ተቋሙ መለያ ቁጥር .... Koodii dhaabbata fayyaa
የጤና ኤክስቴንሽን ሠ. ስም 1. .... Maqaa hojjettu 2. .... ekisteenshinii fayyaa	ወር እና ዓ.ም. .... Ji'aa fii bara

ክፍል ለ: ስለ ዉልደቶች (በህይወት የተወለዱትን ብቻ ይመዝገብ)
Kutaa B: Waa'ee dhalootaa (Lubbuu dhaan kan dhalatan qofa galmeessi)

ተ.ቁ Lakk	የህጻኑ እናት የቤተሰብ መለያ ቁ. ከቤተሰብ ማህደር Galmee maatii irraa Lakk. Haadha mucaa	የህጻኑ እናት መለያ ቁጥር ከቤተሰብ ማህደር Lakk. Haadha mucaa galmee maatii irraa	የህጻኑ ጾታ Saala mucaa	የተወለደበት ቀን ወር እና ዓ.ም. Guyyaa fii ji'a dhalate	የተወለደበት ቦታ ቤት/ያለልምድ አዋላጅ = 1 ቤት/በልምድ አዋላጅ =2 ጤና ጣቢያ/ኬላ = 3 ሆስፒታል =4 ሌላ ይገለጽ Bakka dhalootaa -Mana/Deesiftuu addaa malee =1 -Mana/ dessifituu aadaa tiin = 2 - Bu. Fayyaa/Kella =3 -Hoospitaala = 4 -Kan birraa ibsi ________	አስተያየት ካለ Yaada (yoo jiraate)
(ለ 01)	(ለ 02)	(ለ 03)	(ለ 04)	(ለ 05)	(ለ 06)	(ለ 07)
01			ወ Dhi. ሴ Dha.	_ _ / _ _ /_ _ _ _		

Real Time MORTALITY MONITORING
MONTHLY HEW RECORDING OF VITAL EVENTS EXTRACTION FORM
JOHNS HOPKINS UNIVERSITY – MIZ HASAB RESERCH CENTER

☐መረጃዎች ሁሉ መሰብሰቢያ ቅጽ
Guca odeeffannoon ittiin sasaabamu

ዞን .... ወረዳ ....<br>Godina Aanaa	የቀበሌዉ ስም..................................<br>Maqaa gandaa
የጤና ተቋሙ<br>ስም...<br>Maqaa dhaabata fayyaa	የጤና ተቋሙ መለያ ቁጥር<br>..................................<br>Koodii dhaabbata fayyaa
የጤና ኤክስቴንሽን ሠ. ስም 1. ....<br>Maqaa hojjettu 2. ....<br>ekisteenshinii fayyaa	ወር እና ዓ.ም. ....<br>Ji'aa fii bara

ክፍል ለ: ስለ ዉልደት (በህይወት የተወለዱትን ብቻ ይመዝገብ)
Kutaa B: Waa'ee dhalootaa (Lubbuu dhaan kan dhalatan qofa galmeessi)

ተ.ቁ<br>Lakk	የህጻኑ እናት የቤተሰብ መለያ ቁ. ከቤተሰብ ማህደር<br>Galmee maatii irraa Lakk. Haadha mucaa	የህጻኑ እናት መለያ ቁጥር ከቤተሰብ ማህደር<br>Lakk. Haadha mucaa galmee maatii irraa	የህጻኑ ጾታ<br>Saala mucaa	የተወለደበት ቀን ወር እና ዓ.ም.<br>Guyyaa fii ji'a dhalate	የተወለደበት ቦታ<br>ቤት/ያለልምድ አዋላጅ = 1<br>ቤት/በልምድ አዋላጅ =2<br>ጤና ጣቢያ/ኬላ = 3<br>ሆስፒታል =4<br>ሌላ ይገለጽ<br>Bakka dhalootaa<br>-Mana/Deesiftuu addaa malee =1<br>-Mana/ dessifituu aadaa tiin = 2<br>- Bu. Fayyaa/Kella =3<br>-Hoospitaala = 4<br>-Kan birraa ibsi<br>________	አስተያየት ካለ<br><br>Yaada (yoo jiraate)
(ለ 01)	(ለ 02)	(ለ 03)	(ለ 04)	(ለ 05)	(ለ 06)	(ለ 07)
01			ወ Dhi.<br>ሴ Dha.	_ _ / _ _ /_ _ _ _		
02			ወ Dhi.<br>ሴ Dha.	_ _ / _ _ /_ _ _ _		
03			ወ Dhi.<br>ሴ Dha.	_ _ / _ _ /_ _ _ _		
04			ወ Dhi.<br>ሴ Dha.	_ _ / _ _ /_ _ _ _		
05			ወ Dhi.<br>ሴ Dha.	_ _ / _ _ /_ _ _ _		

Real Time MORTALITY MONITORING
MONTHLY HEW RECORDING OF VITAL EVENTS DATA COLLECTION FORM

JOHNS HOPKINS UNIVERSITY – MIZ HASAB RESERCH CENTER

መረጃ መሰብሰቢያ ቅጽ

Guca odeeffannoon ittiin sasaabamu

**ዞን .... ወረዳ ....**<br>**Godina Aanaa**	**የቀበሌዉ ስም...**<br>**Maqaa gandaa**
**የጤና ተቋሙ ስም...**<br>**Maqaa dhaabata fayyaa**	**የጤና ተቋሙ መለያ ቁጥር ....**<br>**Koodii dhaabbata fayyaa**
**የጤና ኤክስቴንሽን ሠ. ስም 1. ....**<br>**Maqaa hojjettu 2. ....**<br>**ekisteenshinii fayyaa**	**ወር እና ዓ.ም. ....**<br>**Ji'aa fii bara**

ክፍል ሐ ፡ ህልፈት (ሞት) Kutaa C: Ergarama (Du'a)

**ተ.ቁ**<br>lakk	**የሞተው ሰው የቤተሰብ መለያ ቁጥር ከቤተሰብ ማህደር Koodii maatii nama dwee galmmee maatii keessaa**	***የሞተው ሰው መለያ ቁጥር ከቤተሰብ ማህደር *Koodii nama du'ee galmee maatii keessaa**	**የተወለደበት ቀን ቀን/ወር/ዓ.ም Guyyaa dhalota guyyaa/Ji'a. Bara**	**ፆታ**<br>**Saala**	**የሞተበት ቀን**<br>**ቀን/ወር/ዓ.ም Guyyaa du'e Guyyaa/Ji'a/Bara**	****ዕድሜ Umurii**	**የሞተበት ቦታ (ጤና ተቋም ውስጥ ከሆነ ስሙ ይጠቀስ)** Bakka itti du'e (dhaa bbata fayyaa yoo ta'e maqaan haa ibsamu)	**የተመዘገበው የአሟሟቱ ምክንያት** Du'aaf sababa kan ta'ee galmaa'ee	***ማስታዎሻ:-የሞተው እድሜው 12 እና ከዚያ በላይ የሆናት ሴት ከሆነች የሚከተለውን ጠይቅ Yaadaanoo: kan du;e umuriin issa 12 fii isaa ol dubartii taate yoo ta'e kanatti fufee kan jiru gaafadhu***		
									**ስትሞት እርጉዝ ነበረች?** Yeroo duutu ulfa turtee? **እዎ 1 EEyee የለም 2** Lakki	**የሞተችው በወሊድ ምክንያት ነው? Kan duute Sababa ulfaatiinii? እዎ 1 EEyee የለም 2** Lakki	**የሞተችው በወለደች በ 2 ወር ዉስጥ ነው? Kanduute eega deessee jj'a 2 keessat dhaa? እዎ 1 EEyee የለም 2** Lakki
(ሐ 01)	(ሐ 02)	(ሐ 03)	(ሐ 04)	(ሐ 05)	(ሐ 06)	(ሐ 07)	(ሐ 08)	(ሐ 09)	(ሐ 10)	(ሐ 11)	(ሐ 12)
**01**			__ /__ /_ _ _ _	ወ Dhi.<br>ሴ Dha.	__ /__ /_ _ _ _						

***** የሞተው ሰው እድሜው ከ 1 ወር በታች ከሆነ ዕድሜው በቀን ይገለፅ፤ዕድሜው ከ 2 ዓመት በታች ከሆነ በወራት ይገለፅ፤እባክዎን በቁጥሩ ጎን ቀን ወይም ወር ብለው መፃፍዎን አይዘንጉ::ቲ***

***** Umuriin Isa du'ee ji'a 1 gad yoo ta'e, guyyaadhaan haa ibsamu; ji'a 2 gad yoo ta'e ji'aan haa ibsamu; Adaraa keessan lakkofsicha bukkeet guyyaa Ykn ji'a jettanii barreessuu hin dagatiinaa***

*** ተወልደዉ ሳይቆዩ ለሞቱ ህጻናት የ እናትየዋን መለያ ቁጥር ተጠቀሙ::**

*** Daa;imman dhalatanii osoo hin turiin du'aniif koodii haadhaatti fayyadamaa.**

Real Time MORTALITY MONITORING MONTHLY HEW RECORDING OF VITAL EVENTS EXTRACTION FORM JOHNS HOPKINS UNIVERSITY – MIZ HASAB RESERCH CENTER

□መረጃዎች ሁሉ መሰብሰቢያ ቅጽ Guca odeeffannoon ittiin sasaabamu

ዞን ………………………… ወረዳ ………………… Godina Aanaa	የቀበሌዉ ስም………………………… Maqaa gandaa
የጤና ተቋሙ ስም……………………………………… Maqaa dhaabata fayyaa	የጤና ተቋሙ መለያ ቁጥር ………………… Koodii dhaabbata fayyaa
የጤና ኤክስቴንሽን ሠ. ስም 1. ………… 2. ………… Maqaa hojjettu ekisteenshinii fayyaa	ወር እና ዓ.ም. ……………………… Ji'aa fii bara

ክፍል ሐ፡ ህልፈቶች (ሞት) Kutaa C: Ergarama (Du'a)

ተ.ቁ lakk	የሞተው ሰው የቤተሰብ መለያ ቁጥር ከቤተሰብ ማህደር Koodii maatii nama dwee galmmee maatii keessaa	*የሞተው ሰው መለያ ቁጥር ከቤተሰብ ማህደር *Koodii nama du'ee galmee maatii keessaa	የተወለደበት ቀን ቀን/ወር/ዓ.ም Guyyaa dhalota guyyaa/Ji'a. Bara	ፆታ Saala	የሞተበት ቀን ቀን/ወር/ዓ.ም Guyyaa du'e Guyyaa/Ji'a/Bara	**ዕድሜ Umurii	የሞተበት ቦታ (ጤና ተቋም ውስጥ ከሆነ ስሙ ይጠቀስ) Bakka itti du'e (dhaa bbata fayyaa yoo ta'e maqaan haa ibsamu)	የአሟሟቱ ምክንያት Du'aaf sababa kan ta'ee	*ማስታዎሻ:-የሞተው እድሜው 12 እና ከዚያ በላይ የሆናት ሴት ከሆነች የሚከተለውን ጠይቅ Yaadaanoo: kan du;e umuriin issa 12 fii isaa ol dubartii taate yoo ta'e kanatti fufee kan jiru gaafadhu*		
									ስትሞት እርጉዝ ነበረች? Yeroo duutu ulfa turtee? አዎ 1 EEyee የለም 2 Lakki	የሞተችው በወሊድ ምክንያት ነው? Kan duute Sababa ulfaatiinii? አዎ 1 EEyee የለም 2 Lakki	የሞተችው በወለደች በ 2 ወር ዉስጥ ነው? Kanduute eega deessee jj'a 2 keessat dhaa? አዎ 1 EEyee የለም 2 Lakki
(ሐ 01)	(ሐ 02)	(ሐ 03)	(ሐ 04)	(ሐ 05)	(ሐ 06)	(ሐ 07)	(ሐ 08)	(ሐ 09)	(ሐ 10)	(ሐ 11)	(ሐ 12)
01			__ /__ /_ _ _ _	ወ Dhi. ሴ Dha.	__ /__ /_ _ _ _						
02			__ /__ /_ _ _ _	ወ Dhi. ሴ Dha	__ /__ /_ _ _ _						
04			__ /__ /_ _ _ _	ወ Dhi. ሴ Dha	__ /__ /_ _ _ _						
05			__ /__ /_ _ _ _	ወ Dhi. ሴ Dha	__ /__ /_ _ _ _						
06			__ /__ /_ _ _ _	ወ Dhi. ሴ Dha	__ /__ /_ _ _ _						
07			__ /__ /_ _ _ _	ወ Dhi. ሴ Dha	__ /__ /_ _ _ _						

*** የሞተው ሰው እድሜው ከ 1 ወር በታች ከሆነ ዕድሜው በቀን ይገለፅ፤ዕድሜው ከ 2 ዓመት በታች ከሆነ በወራት ይገለፅ፤እባክዎን በቁጥሩ ጎን ቀን ወይም ወር ብለው መፃፍዎን አይዘንጉ::ቲ*

** Umuriin Isa du'ee ji'a 1 gad yoo ta'e, guyyaadhaan haa ibsamu; ji'a 2 gad yoo ta'e ji'aan haa ibsamu; Adaraa keessan lakkofsicha bukkeet guyyaa Ykn ji'a jettanii barreessuu hin dagatiinaa.*

*** ተወልደዉ ሳይቆዩ ለሞቱ ህጻናት የ እናትየዋን መለያ ቁጥር ተጠቀሙ::**

*** Daa;imman dhalatanii osoo hin turiin du'aniif koodii haadhaatti fayyadamaa.**

ተረጋግጧል: የሱፐርቫይዘሩ ስም: ……………………… ፊርማ ……………………… ቀን ………………………

Mirkaneefameera: Maqaa suparvayizarichaa: ____________ Mallattoo____________ Guyyaa____________
